# Illuminating proinflammatory myeloid cells with PET tracers targeting GPR84

**DOI:** 10.1073/pnas.2536372123

**Published:** 2026-05-21

**Authors:** Mausam Kalita, Renesmee C. Kuo, Valentina Straniero, Samantha T. Reyes, Mallesh Pandrala, Alessia Lanzini, Sara Marsango, Desiree D’Moore, Piper Mahn, Andrew Setiadi, Mira Sundar, Spencer Mak, Sydney Nagy, Israt S. Alam, Poorva Jain, Grace Inay, Rim Malek, Allen F. Brooks, Corinne Beinat, Ermanno Valoti, Peter J. H. Scott, Graeme Milligan, Michelle L. James

**Affiliations:** ^a^ Department of Radiology, Stanford University, Stanford, CA 94305; ^b^ Department of Electrical Engineering, Stanford University, Stanford, CA 94305; ^c^ Department of Pharmaceutical Sciences, University of Milan, Milano, Italy 20133; ^d^ Centre for Translational Pharmacology, School of Molecular Biosciences, College of Medical, Veterinary and Life Sciences, University of Glasgow, Glasgow, Scotland, United Kingdom G12 8QQ; ^e^Division of Nuclear Medicine, Department of Radiology, University of Michigan Medical School, Ann Arbor, MI 48109; ^f^ Department of Neurology and Neurological Sciences, Stanford University, Stanford, CA 94305

**Keywords:** positron emission tomography (PET), G protein–coupled receptor 84 (GPR84), innate immune activation, Alzheimer’s disease, neuroinflammation

## Abstract

Innate immunity mediated by myeloid cells defends against infection and injury, but when chronically activated, it drives tissue damage and neurodegeneration. Molecular imaging with positron emission tomography (PET) enables noninvasive, real-time monitoring of such processes in vivo. However, most current neuroinflammation PET tracers lack specificity for activated myeloid cells. G protein–coupled receptor 84 (GPR84) is a promising biomarker that is selectively upregulated on activated microglia and macrophages. Here, we report the development and validation of two fluorine-18-labeled GPR84 tracers, [^18^F]MGX-110S and [^18^F]MGX-111S. Both exhibit specific binding to human GPR84-expressing cells, with [^18^F]MGX-110S demonstrating superior affinity, selectivity, and signal-to-background ratio. [^18^F]MGX-110S enables sensitive detection of systemic- and neuro-inflammation in LPS-treated mice and outperforms PET images obtained using a radiotracer specific for translocator protein 18 kDa in 5xFAD mice—revealing pathology-correlated activation across cortical, hippocampal, and thalamic regions. Taken together, our data indicate that [^18^F]MGX-110S is a highly sensitive and specific tool for visualizing maladaptive myeloid cell activation; its clinical translation could enable more precise detection and staging of inflammation in addition to improved therapeutic monitoring in neurodegenerative disorders and more broadly in inflammatory diseases.

## Significance

Neuroinflammation plays a central role in many neurological diseases including Alzheimer’s disease, yet current imaging tools cannot specifically detect the maladaptive myeloid responses that play a central role in disease progression. Herein, we identify GPR84 as a highly inducible, myeloid-specific biomarker of innate immune activation and develop fluorine-18-labeled PET tracers to image this target in vivo. Our lead tracer, [^18^F]MGX-110S, crosses the intact blood-brain barrier, detects early microglial activation in 5xFAD mice with higher sensitivity than translocator protein 18 kDa-positron emission tomography, and correlates with CD68^+^ myeloid cells. This work establishes GPR84-PET as a promising next-generation imaging approach for earlier detection, more precise staging, and therapeutic monitoring across neuroinflammatory and neurodegenerative diseases.

Neurodegenerative and neuroinflammatory diseases pose a major public health challenge, contributing substantially to global morbidity, disability, and mortality. Mounting evidence implicates dysregulated neuroimmune signaling as a key driver of neuronal injury and disease progression across multiple disorders, which in turn has spurred the development of therapeutic strategies aimed at modulating these pathways. This shift underscores the need for biomarkers that can directly track and quantify these immune processes in vivo (1). Available neuroimaging techniques such as magnetic resonance imaging (MRI) provide valuable information on structural changes but fail to capture specific information on the dynamic immune landscape of the central nervous system (CNS) (2, 3). Similarly, blood- and fluid-based biomarkers are widely accessible and cost-effective, but lack specificity for immune alterations in the CNS and cannot resolve the spatiotemporal patterns of inflammation (4). Thus, there is a critical unmet need for noninvasive imaging strategies that can quantify real-time maladaptive neuroinflammatory responses, complement current clinical imaging standards, and enable earlier detection, more accurate disease staging, and longitudinal monitoring of therapeutic efficacy across neuroinflammatory diseases.

Myeloid-lineage cells, including microglia, macrophages, monocytes, and neutrophils, are highly dynamic regulators of innate immune responses and key drivers of both chronic and acute inflammation in the CNS. Depending on their activation state, these cells can either promote tissue repair and resolution or drive neurotoxicity and degeneration (5, 6). In chronic neurodegenerative conditions such as Alzheimer’s disease (AD), sustained activation of microglia around Aβ plaques leads to the release of proinflammatory cytokines, chemokines, and reactive oxygen species that exacerbate synaptic loss and neuronal injury (7–9). Experimental models using systemic lipopolysaccharide (LPS) administration provide a well-established framework for studying acute neuroinflammatory responses, allowing researchers to dissect the molecular and cellular mechanisms underlying innate immune activation and its contribution to neuronal dysfunction (10).

Positron emission tomography (PET) imaging is a promising approach for longitudinal visualization and quantification of immune responses in vivo. The translocator protein 18 kDa (TSPO) is the most widely explored PET biomarker of neuroinflammation and has been instrumental in establishing proof of principle for imaging neuroimmune dynamics (11–13). However, TSPO’s functional role in AD and related disorders remains poorly understood, and its narrow dynamic range between healthy and diseased brains (14, 15), along with its lack of microglial specificity (16, 17), further complicates clinical interpretation. These limitations hinder the sensitivity of TSPO-PET for detecting neuroinflammation. Alternative targets such as colony-stimulating factor 1 receptor (CSF1R) and purinergic P2X7 receptor (P2X7R) have been explored as more selective biomarkers of myeloid activation, but like TSPO, they are not truly specific for microglia and/or cannot reliably distinguish between beneficial and maladaptive innate immune responses (18, 19). This lack of functional specificity limits the clinical utility of imaging such biomarkers for identifying disease-relevant immune responses and guiding the development of targeted immunomodulatory therapies.

To overcome these limitations, we identified G protein-coupled receptor 84 (GPR84) as a promising biomarker for imaging innate immune activation. GPR84 is a seven-transmembrane domain orphan G protein-coupled receptor (GPCR) selectively expressed on activated microglia and other myeloid-lineage cells (20–22), where it amplifies proinflammatory signaling cascades. It is activated by endogenous medium-chain fatty acids (MCFA), and its expression is robustly upregulated in murine macrophages and human monocyte-derived macrophages (hMDMs) following stimulation with proinflammatory agents such as LPS and tumor necrosis factor (TNF), but not by anti-inflammatory cytokines such as interleukin-4 (IL-4) (23, 24). Elevated GPR84 expression has been implicated in a wide range of systemic and neurological diseases involving maladaptive innate immune responses, including inflammatory bowel disease, neuropathic pain, stroke, and AD. In the CNS, GPR84 is predominantly localized to activated microglia and infiltrating myeloid cells and plays a critical role in microglial recruitment to Aβ plaques, with significant upregulation in murine models of AD (25, 26). These features position GPR84 as a functionally relevant and highly specific biomarker of pathogenic innate immunity, offering an avenue for sensitive and mechanistically informed neuroinflammation imaging.

Here, we leverage recent advances in copper-mediated radiofluorination to develop ^18^F-labeled GPR84-PET tracers. Using rational CNS molecule design principles and CNS multiparameter optimization (MPO) filters (*SI Appendix*, Fig. S1) (27–29), we identified two promising candidates, [^18^F]MGX-110S and [^18^F]MGX-111S. Subsequently, we developed, characterized, and validated both tracers, and evaluated our lead candidate, [^18^F]MGX-110S, for PET imaging of GPR84 in an AD mouse model compared to TSPO-PET. This study lays the foundation for quantitative visualization of innate immune activation in the brain and whole body in future clinical studies.

## Results

### Development of a Streamlined Synthetic Platform for Generation of GPR84-PET Tracers.

**Scheme 1.**
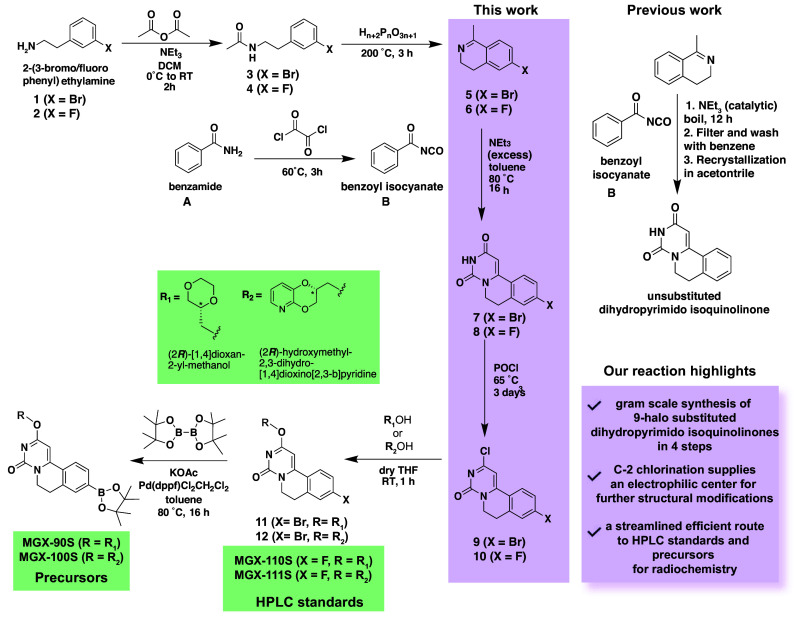
Synthetic routes to HPLC standards (MGX-110S and MGX-111S) and BPin precursors (MGX-90 and MGX-100) used for the synthesis of [^18^F]MGX-110S and [^18^F]MGX-111S radiotracers.

To prepare [^18^F]MGX-110S and [^18^F]MGX-111S, we utilized copper-mediated radiofluorination of the corresponding boronic pinacol ester (BPin) esters, which has recently emerged as a powerful method for radiolabeling diverse aromatic radiopharmaceuticals inaccessible by more traditional methods such as nucleophilic aromatic substitutions (30, 31). Our initial attempt to synthesize precursors using a modified 10-step route (32) was inefficient, requiring multiple protection-deprotection steps, affording the BPin precursor in only 7.8% overall yield (MGX-90S, *SI Appendix*, Scheme S1), and failing to produce the ^19^F reference standards. These challenges prompted us to design a concise synthetic platform centered on 9-bromo- and 9-fluoro-substituted dihydropyrimido-isoquinolinone intermediates—versatile building blocks for both radiochemistry precursors and reference standards (Scheme 1).

Translating this synthetic strategy into practice required overcoming difficulties during cyclization, as early attempts were hindered by the electron-withdrawing bromo substituent (*SI Appendix*, Table S1 and Scheme S2). Through systemic optimization of a benzoyl isocyanate-mediated ring-closure approach, we established a robust, high-yielding six-step synthesis of 9-halo-dihydropyrimido-isoquinolinone scaffolds (overall chemical yields of ~14.8% for 9-fluoro reference standards and ~20.8% for 9-bromo compounds), enabling efficient access to both target GPR84-PET tracers and their corresponding ^19^F standards. This route begins with cyclization of N-(3-halo-phenethyl)acetamides (**3** or **4**, Scheme 1) in polyphosphoric acid at 200 °C for 3 h to yield 6-halo-1-methyl-3,4-dihydroisoquinolines (**5**, **6**). Subsequent reaction of **5** or **6** with in situ-generated benzoyl isocyanate afforded the 9-halo-6,7-dihydropyrimido[6,1-a]isoquinoline-2,4-diones (**7**, **8**). Using excess triethylamine (TEA, 4 equivalents), we accelerated reaction kinetics and suppressed byproduct formation due to the instability of benzoyl isocyanate. Since **7** and **8** are insoluble in common organic solvents, they were purified via sequential water/methanol slurry and filtration. Final C-2 chlorination with phosphorus oxychloride (POCl_3_) produced intermediates **9** and **10**, which were then converted into the ^19^F reference standards (MGX-110S, MGX-111S) and BPin precursors (MGX-90S, MGX-100S) for ^18^F radiochemistry (Scheme 1).

### Optimization of Manual and Automated ^18^F Radiosyntheses for GPR84 Tracers.

**Scheme 2.**
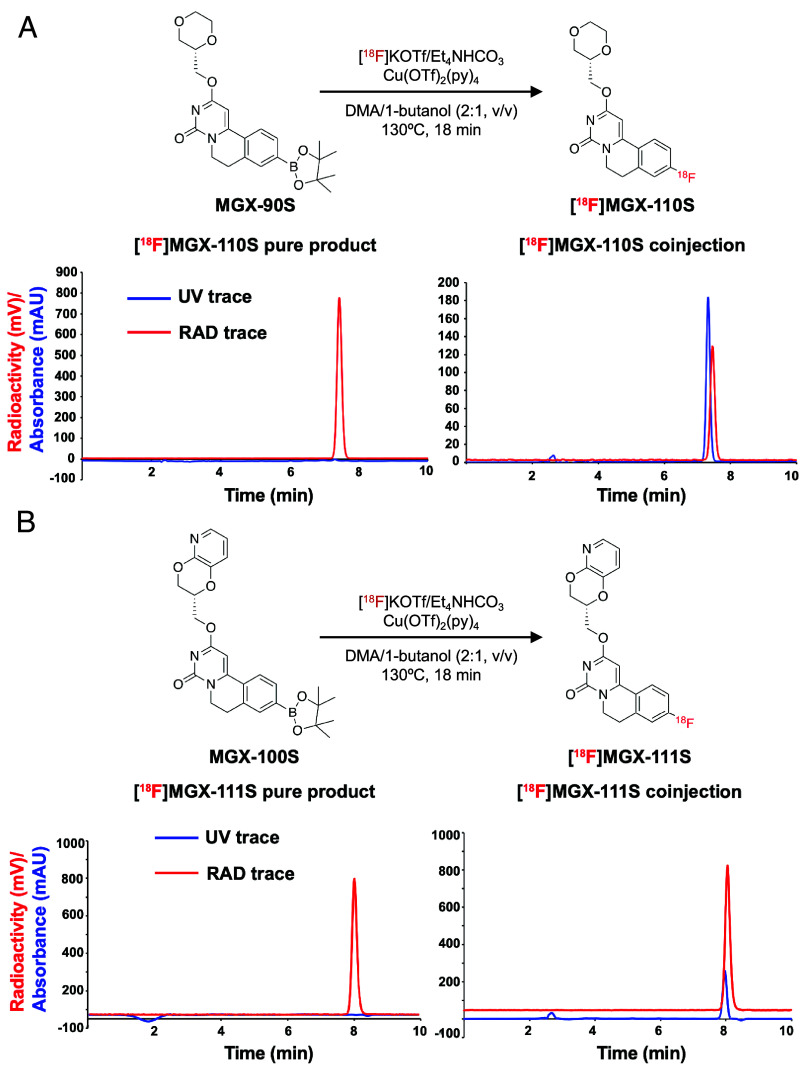
Radiosynthesis and analytical HPLC characterization of final pure (*A*) [^18^F]MGX-110S and (*B*) [^18^F]MGX-111S tracers.

Manual radiosynthesis of the candidate GPR84 tracers involved three main steps: i) ^18^F elution and azeotropic drying, ii) copper(II)-mediated radiofluorination, and iii) semipreparative HPLC purification and formulation. Both the potassium triflate (KOTf/Et_4_NH_4_HCO_3_) and kryptofix methods achieved >90% ^18^F elution efficiency, but only the potassium triflate method enabled successful ^18^F labeling of both tracers (*SI Appendix*, Table S2) (33, 34). Optimization of elution and reaction solvents, temperature, and copper(II) catalyst equivalents identified the most efficient conditions (*SI Appendix*, Tables S3 and S4): azeotropically dried ^18^F in dimethylacetamide (DMA)/n-butanol (2/1, v/v), reacted with Cu(OTf)_2_(py)_4_ (1 eq) and precursor (2 to 3 mg) at 130 °C for 20 min. Radio-TLC confirmed radiochemical conversion and purity of the final products, yielding modest radiochemical yields (RCY) and molar activities ([^18^F]MGX-110S: 10.0 ± 0.03%, 132.5 ± 15.9 GBq/µmol; [^18^F]MGX-111S: 12.4 ± 0.7%, 75.9 ± 20.0 GBq/µmol; *SI Appendix,* Figs. S2 and S3), and HPLC coinjection with ^19^F reference standards verified tracer identity (MGX-110S: 7.32 vs. 7.47 min [standard vs. tracer], MGX-111S: 7.97 vs. 8.05 min; Scheme 2).

As a step toward clinical translation, we next optimized fully automated radiosynthesis methods for [^18^F]MGX-110S and [^18^F]MGX-111S using a TRACERLab FX_FN_ module (Scheme 2 and *SI Appendix*, Fig. S4). Prior automated methods using BPin precursors suffered from low RCY and molar activity, as well as protodeboronation of the carbon-boron bond (35). These issues often require complex workarounds such as predissolution of ^18^F in reaction solvents, altered reagent addition sequences, air exposure, or specialized perfluorophenyl-capped columns to separate the radiotracer from nonradioactive, protodeboronated side-products (36, 37). To overcome these challenges, we systematically screened elution solvents, copper mediators, and reaction conditions (*SI Appendix*, Table S5). Optimal performance was achieved using Cu(OTf)_2_ (5 eq), BPin precursor (1 eq), and neat pyridine (125 eq), with reagents added immediately prior to [^18^F]fluoride delivery in [^18^O]H_2_O. Notably, conducting the automated synthesis under an inert helium atmosphere, relative to manual radiochemistry—doubled RCY and molar activity—while fully suppressing protodeboronation side-product formation (*SI Appendix*, Table S6). Following semipreparative HPLC purification and reformulation in ethanol/saline (5:95, v/v; *SI Appendix*, Fig. S5), the optimized workflow reproducibly yielded [^18^F]MGX-110S and [^18^F]MGX-111S with non-decay-corrected RCY of 21.9 ± 0.01% and 15.6 ± 4.3%, and molar activities of 246.8 ± 7.8 GBq/µmol and 106.6 ± 43.7 GBq/µmol, respectively (n = 5; *SI Appendix*, Table S7). This synthetic platform provides a robust, reproducible automated method for producing many ^18^F-labeled tracers with high radiochemical purity and molar activity, suitable for preclinical evaluation and future clinical translation.

### In Vitro Characterization of [^18^F]MGX-110S and [^18^F]MGX-111S.

**Fig. 1.**
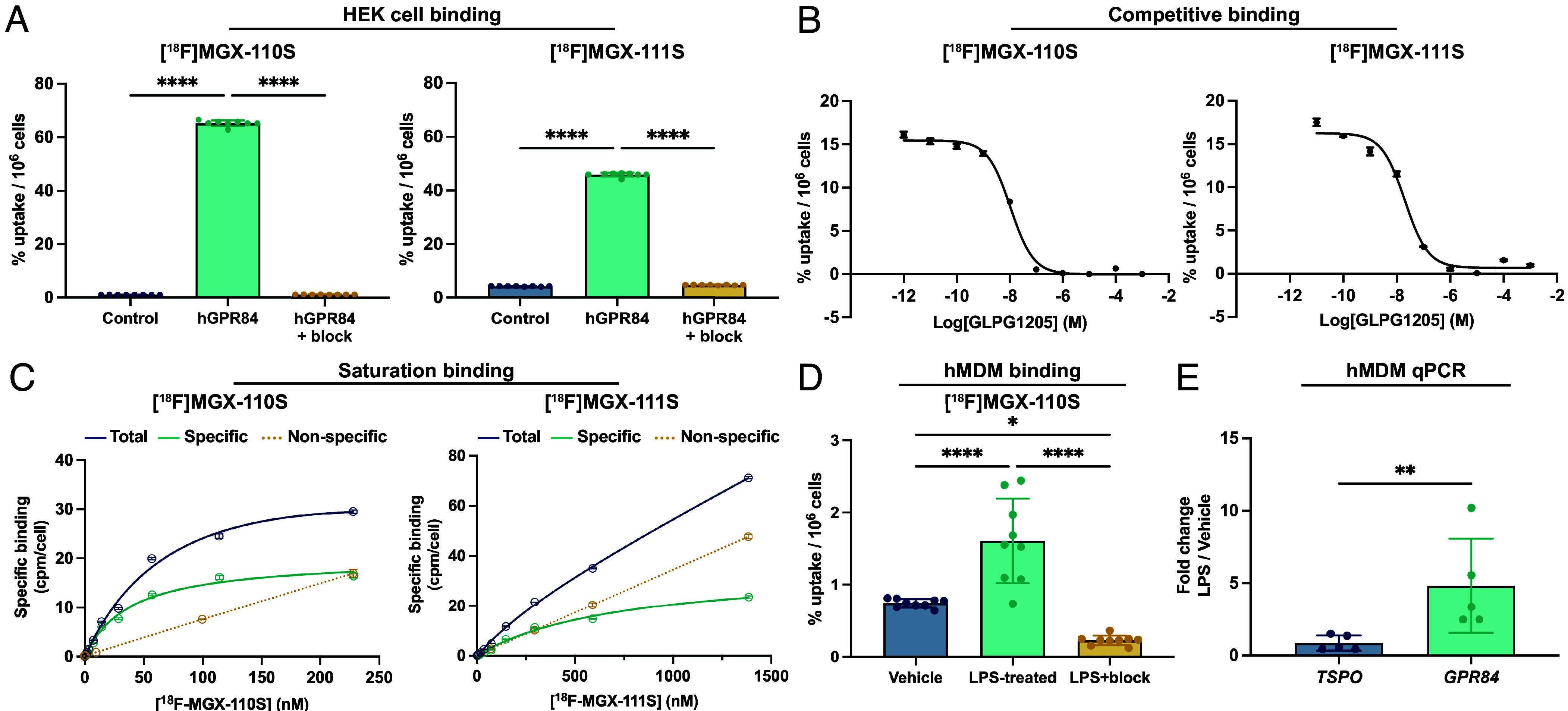
In vitro GPR84-PET tracer validation confirms target-specific binding and identifies [^18^F]MGX-110S as the lead candidate. (*A*) Cell binding assays showed markedly increased uptake of [^18^F]MGX-110S and [^18^F]MGX-111S in HEK293 cells expressing hGPR84 compared to parental controls, with tracer binding blocked by coincubation with the GPR84 antagonist GLPG1205 (35 µM). (*B*) Competitive binding assays confirmed tracer binding specificity, with IC_50_ values of 10.67 nM for [^18^F]MGX-110S and 20.25 nM for [^18^F]MGX-111S. (*C*) Saturation binding studies revealed higher affinity of [^18^F]MGX-110S (K_D_ = 38.1 nM) compared to [^18^F]MGX-111S (K_D_ = 266 nM). (*D*) In hMDMs, LPS (100 ng/mL) stimulation increased [^18^F]MGX-110S uptake 2.2-fold relative to saline-treated vehicle cells. Binding was reduced to baseline following coincubation with GLPG1205. (*E*) qPCR analysis of hMDMs demonstrated approximately fivefold upregulation of *GPR84* mRNA following LPS stimulation, whereas TSPO expression remained unchanged. Statistical analyses were performed using (*A* and *D*) one-way ANOVA with Tukey’s multiple comparisons test and (*E*) Mann–Whitney test. *P < 0.05, **P < 0.01, ****P < 0.0001. Data are shown as mean ± SD.

We next evaluated the binding specificity of [^18^F]MGX-110S and [^18^F]MGX-111S for human GPR84 (hGPR84) using two cell-based assays. In HEK293 cells stably overexpressing hGPR84, [^18^F]MGX-110S showed a 64.5-fold increase in binding compared to parental controls after 40 min of incubation (*P* < 0.0001), which was reduced by 98.1% upon coincubation with the structurally distinct GPR84 antagonist GLPG1205 (35 µM; *P* < 0.0001, n = 8), confirming specificity (Fig. 1*A*). In contrast, [^18^F]MGX-111S demonstrated a more modest 10.7-fold increase in hGPR84^+^ HEK293 binding, which was reduced by 90.5% with GLPG1205 blocking (*P* < 0.0001). Competitive binding assays with GLPG1205 revealed a lower inhibition constant for [^18^F]MGX-110S (IC_50_ = 10.67 nM) compared to [^18^F]MGX-111S (IC_50_ = 20.25 nM; Fig. 1*B*), indicating stronger receptor affinity for [^18^F]MGX-110S. Saturation binding studies further corroborated these findings, showing substantially higher affinity (approximately a sixfold difference) for [^18^F]MGX-110S (*K_D_* = 38.06 nM) than for [^18^F]MGX-111S (*K_D_* = 226 nM; Fig. 1*C*). In hMDMs, stimulation with LPS (100 ng/mL, 24 h) increased [^18^F]MGX-110S binding 2.2-fold compared to vehicle-treated cells with saline, an effect abrogated by coincubation with GLPG1205 (35 µM) (87.6% reduction; Fig. 1*D*; *P* < 0.0001). Consistent with these results, qPCR analysis revealed a fivefold upregulation of *GPR84* mRNA in LPS-stimulated hMDMs, whereas TSPO expression remained unchanged (Fig. 1*E*). Together, these data demonstrate that [^18^F]MGX-110S binds to GPR84 with low nanomolar affinity, shows superior specificity and sensitivity relative to [^18^F]MGX-111S, and reliably detects proinflammatory myeloid activation, supporting its advancement as the lead radiotracer for further in vivo evaluation.

### [^18^F]MGX-110S in Healthy and LPS-Challenged Mice.

**Fig. 2.**
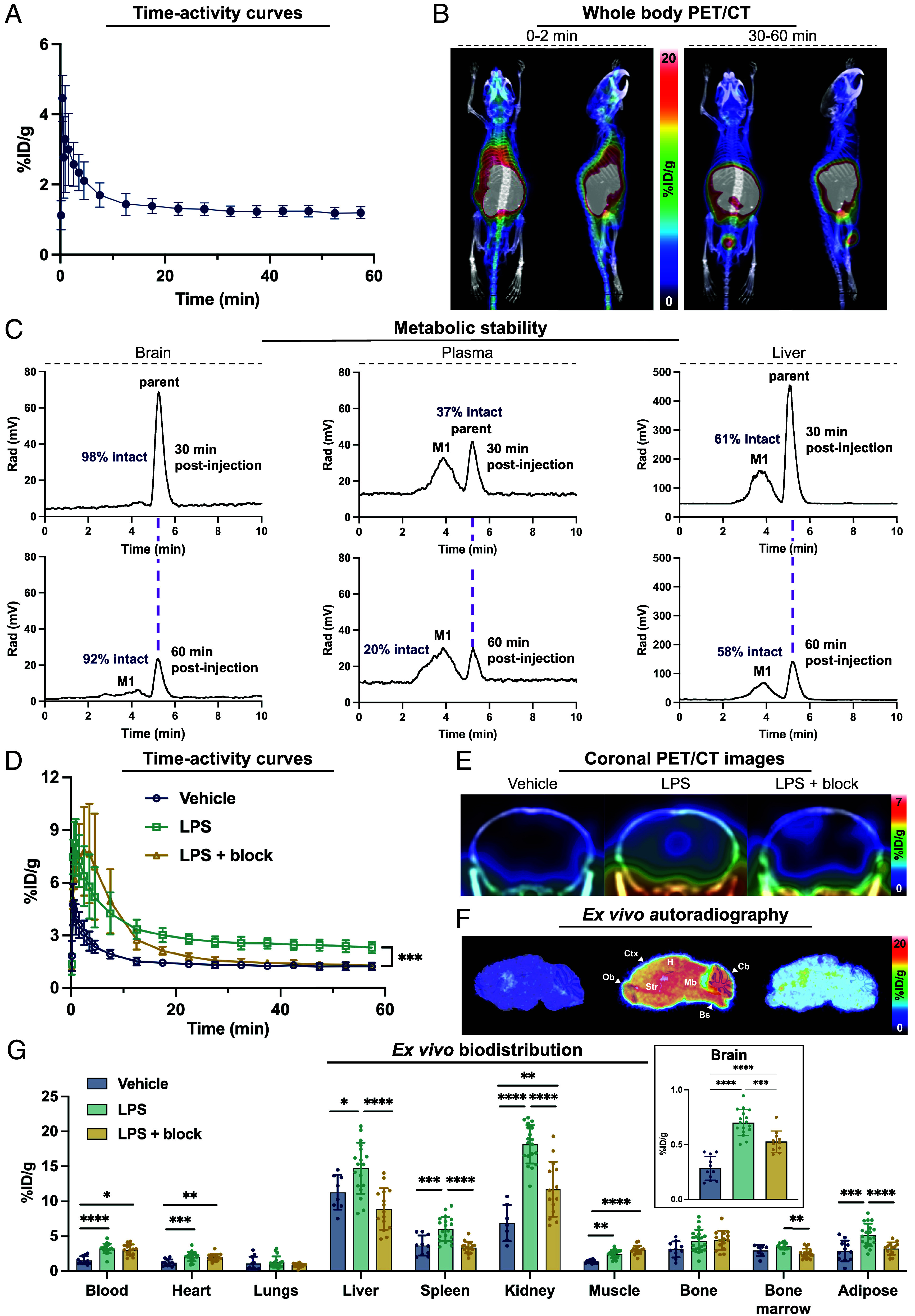
GPR84-PET tracer crosses the healthy murine blood-brain barrier and permits imaging of systemic and neuroinflammation in LPS-treated mice. (*A*) Time-activity curves (TACs) of whole brain over 60 min in healthy female C57BL/J mice. (*B*) Representative whole-body PET/CT maximum intensity projection (MIP) images showing tracer distribution in healthy mice at early (0 to 2 min) and late (30 to 60 min) timepoints. (*C*) Metabolic stability of [^18^F]MGX-110S in the brain, plasma, and liver at 30 and 60 min postinjection. (*D*) Whole-brain TACs from vehicle- and LPS-treated mice; blocking with GPR84 antagonist GLPG1205 confirmed tracer specificity. (*E*) Representative coronal PET/CT images (40 to 50 min summed) from vehicle-, LPS-, and LPS-treated mice with blocking. (*F*) High-resolution ex vivo autoradiography of brain sections confirming increased tracer binding in LPS mice and blocking with GLPG1205. (*G*) Ex vivo gamma counting of brain and peripheral organs 60 min postinjection in vehicle-, LPS-, and LPS-treated with blocking mice. Statistical analyses were performed using the unpaired *t* test (*D*) or one-way ANOVA and Tukey’s multiple comparison test (*G*). *P < 0.05, **P < 0.01, ***P < 0.001, ****P < 0.0001. n = 5 for naïve brain TACs; n = 3 per timepoint for metabolic stability; LPS studies: n = 6 to 11 vehicle-treated, n = 5 to 20 LPS-treated, and n = 4 to 16 LPS-treated with blocking. Cb = cerebellum, Ctx = cortex, Str = striatum, H = hippocampus, Mb = midbrain, Ob = olfactory bulb, and Bs = brainstem. Data are shown as mean ± SD.

After identifying [^18^F]MGX-110S as our lead GPR84-PET tracer, we evaluated its ability to cross the intact blood-brain barrier (BBB) and its metabolic stability in vivo. Dynamic PET/computed tomography (CT) imaging was commenced immediately prior to tracer injection (5.55 MBq) in healthy female C57BL/6 mice. Time-activity curves (TACs) demonstrated rapid BBB penetration, with peak brain uptake reaching 4.59 ± 1.43 %ID/g (percent injected dose per gram) in n = 6 mice within the first few minutes postinjection, followed by gradual washout to 1.25 ± 0.19 %ID/g at 60 min (Fig. 2*A*). Whole-body PET/CT images further confirmed rapid brain entry and favorable elimination kinetics through renal and hepatobiliary pathways (Fig. 2*B*). To validate tracer stability, we quantified the proportion of intact [^18^F]MGX-110S in the brain, plasma, and liver at 30- and 60-min postinjection using analytical radio-HPLC (Fig. 2*C*). Tracer stability was markedly higher in the brain than in plasma: [^18^F]MGX-110S remained 98% intact at 30 min and 92% at 60 min in the brain, whereas only 37% and 20% parent tracer was detected in plasma at the same timepoints. Liver tissue displayed intermediate stability (61% and 58% intact at 30 and 60 min, respectively).

We next assessed the in vivo performance of [^18^F]MGX-110S in a neuroinflammation model induced by systemic LPS administration (5 mg/kg). All animals were imaged 24 h after LPS or vehicle (saline) injection using 60-min dynamic PET imaging. Cohorts included LPS-treated (n = 5), vehicle-treated (n = 5), and LPS-treated mice receiving coinjection of the GPR84 antagonist GLPG1205 (2 mg/kg; n = 4). LPS administration resulted in significantly elevated whole brain uptake of [^18^F]MGX-110S compared with vehicle controls. Peak uptake reached 8.46 ± 1.37%ID/g in LPS-treated mice versus 5.01 ± 0.92 %ID/g in vehicle controls, corresponding to a 1.69-fold increase (Fig. 2*D*). Co-administration of GLPG1205 reduced peak brain uptake from 8.46 to 7.79 %ID/g, and tracer binding was decreased by 64.30% at the end of the 60 min scan, confirming binding specificity (Fig. 2 *D* and *E*). To further investigate tracer distribution at higher resolution, we performed ex vivo autoradiography on 40 µm sagittal brain sections from these same mice after PET imaging and perfusion (~25 h after LPS administration). Autoradiography enabled clear delineation of brain regions with elevated tracer uptake, corroborating in vivo findings and confirming that increased brain signal was not attributable to blood pool or spillover from adjacent tissues. GPR84-PET signal was elevated across the brain parenchyma, from the olfactory bulb to the medulla (Fig. 2*F*). Biodistribution studies provided further confirmation, demonstrating significantly greater tracer accumulation in the brain as well as peripheral organs, including the spleen, liver, kidney, and adipose tissue, in addition to the blood, in LPS-treated mice compared with controls (Fig. 2*G*).

To investigate the relationship between GPR84 levels and activated myeloid cell populations in LPS-treated mice, we performed flow cytometry on dissociated brain tissue from a separate cohort of mice (n = 10 LPS-treated and n = 5 vehicle-treated). LPS-treated mice showed a 9.5-fold increase in CD11b^+^GPR84^+^CD68^+^ myeloid cells compared with vehicle controls (46.54 ± 6.51% vs. 4.92 ± 1.55% of live single cells), confirming that GPR84 is upregulated on activated myeloid populations during neuroinflammation (*SI Appendix*, Fig. S6).

### GPR84-PET Detects Neuroinflammation Early in 5xFAD Mice.

**Fig. 3.**
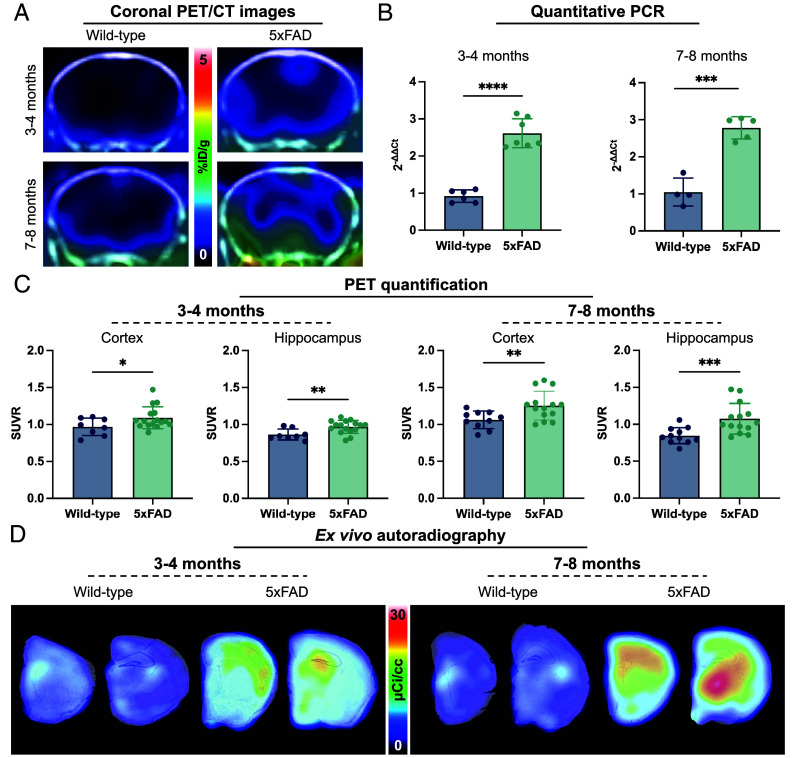
GPR84-PET detects early innate immune activation and enables longitudinal tracking of neuroinflammation in 5xFAD mice. (*A*) Representative coronal PET/CT images showing elevated [^18^F]MGX-110S binding in the cortex and hippocampus of 5xFAD mice compared with age- and sex-matched wild-type controls at both 3 to 4 and 7 to 8 mo of age. (*B*) qPCR analysis of whole brain tissue demonstrating significantly increased *Gpr84* mRNA expression in 5xFAD versus wild-type mice at both time points. (*C*) Quantification of PET images (SUVR, normalized to cerebellum) in the cortex and hippocampus, showing significant tracer binding in 5xFAD compared with wild-type mice at 3 to 4 and 7 to 8 mo. (*D*) Representative ex vivo autoradiography images with Nissl overlay confirming elevated tracer binding in the 5xFAD cortex and hippocampus at 3 to 4 mo, and broader pathology including the cortex and hippocampus at 7 to 8 mo. Statistical analyses were performed using unpaired *t* tests or Mann–Whitney tests. *P < 0.05, **P < 0.01, ***P < 0.001, ****P < 0.0001. For qPCR: n = 4 to 6 wild-types, n = 5 to 7 5xFAD; for in vivo PET quantification: n = 8 to 11 wild-types, n = 14 to 16 5xFAD. Data are shown as mean ± SD.

To investigate the sensitivity of [^18^F]MGX-110S for detecting disease-associated immune activation in the context of AD pathology, we performed PET imaging in the 5xFAD transgenic mouse model. Age- and sex-matched wild-type and 5xFAD mice were imaged at 3 to 4 and 7 to 8 mo of age, representing early and more advanced stages of amyloid pathology and microgliosis (38, 39). Static PET/CT imaging (30 to 40 min postinjection) revealed elevated [^18^F]MGX-110S signal in the cortex and hippocampus of 5xFAD mice compared with wild-type controls (Fig. 3*A*). These imaging results were corroborated by qPCR, which showed increased *Gpr84* mRNA expression in 5xFAD brains (2.84-fold at 3 to 4 mo; 2.66-fold at 7 to 8 mo) relative to controls (Fig. 3*B*).

Tracer binding was quantified as standardized uptake value ratios (SUVRs) using the cerebellum as a reference region, an area which showed neither Aβ pathology nor differential tracer binding between 5xFAD mice and wild-type mice. SUVR normalization also reduced intersubject variability, enabling robust comparisons between groups. At 3 to 4 mo, SUVRs were significantly elevated in the whole brain, cortex, and hippocampus of 5xFAD mice compared with wild-type controls (1.14-fold, 1.13-fold, and 1.12-fold increases, respectively; *P* < 0.05; Fig. 3*C* and *SI Appendix*, Fig. S7*A*), and tracer binding in corresponding regions was significantly reduced by pretreatment with the GPR84 antagonist GLPG1205 (2 mg/kg; *P* ≤ 0.0001; *SI Appendix*, Fig. S7 *B* and *C*). By 7 to 8 mo, tracer accumulation increased further, with cortical SUVRs reaching 1.25 in 5xFAD versus 1.06 in wild-type mice (1.18-fold increase; *P* < 0.01) and hippocampal SUVRs showing a 1.27-fold difference between groups (*P* < 0.001; Fig. 3*C*). In addition, tracer binding was significantly elevated in the thalamus, brainstem, and olfactory bulbs of 7 to 8-mo-old 5xFAD mice (*P* < 0.05; *SI Appendix*, Fig. S7*D*).

To validate in vivo imaging findings, mice were perfused to remove any tracer signal coming from the blood pool, and ex vivo analyses were performed. Ex vivo autoradiography of coronal brain sections confirmed increased [^18^F]MGX-110S binding in the cortex and hippocampus, as well as in the thalamus of 7 to 8-mo-old 5xFAD mice compared with wild-type controls, with blocking confirming binding specificity (Fig. 3*D* and *SI Appendix*, Fig. S7*E*). Collectively, these results demonstrate that GPR84-imaging enables early detection of pathology-associated myeloid cell activation in the cortex and hippocampus and longitudinal tracking of disease progression into subcortical regions as disease advances, establishing this approach as a noninvasive tool for monitoring neuroinflammation in AD progression in vivo.

### GPR84-PET Is More Sensitive Than TSPO-PET in Detecting Early-Stage Neuroinflammation in 5xFAD Mice.

**Fig. 4.**
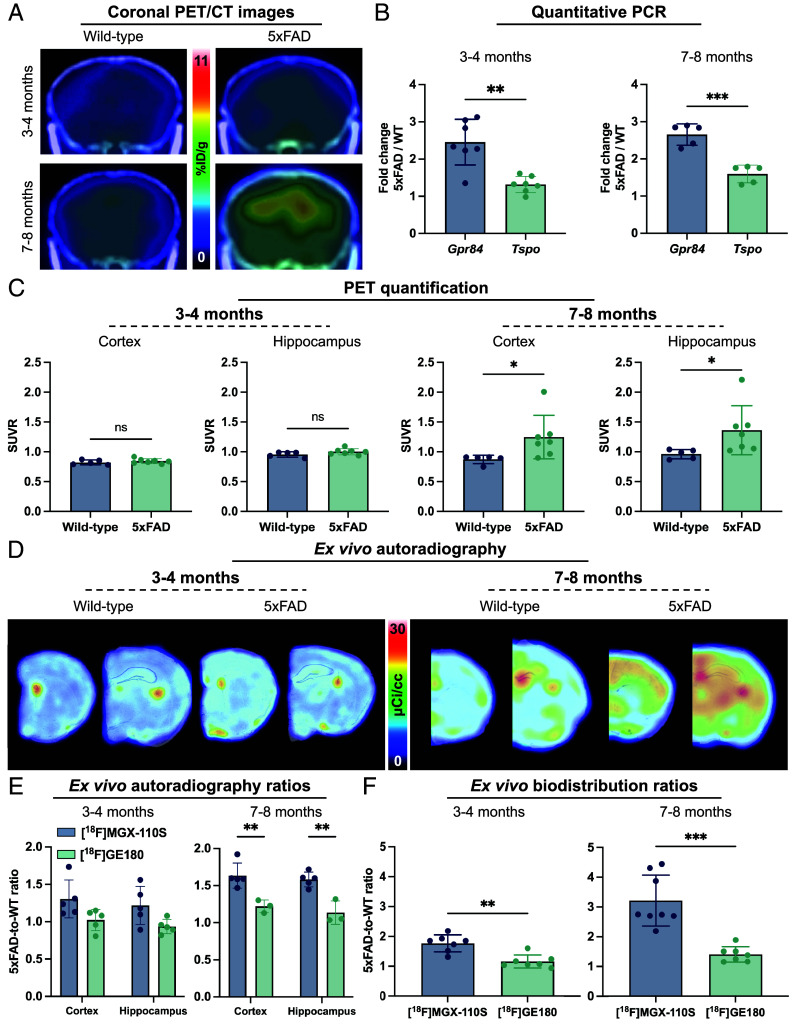
GPR84-PET is more sensitive than TSPO-PET for detecting neuroinflammation in 5xFAD mice. (*A*) Representative coronal [^18^F]GE180 PET/CT images. (*B*) qPCR showing the fold change between 5xFAD and age- and sex-matched wild-type controls at 3 to 4 and 7 to 8 mo comparing *Gpr84* and *Tspo* expression. (*C*) TSPO-PET quantification of the cortex and hippocampus. (*D*) Representative [^18^F]GE180 brain autoradiography images with Nissl overlay. (*E*) 5xFAD-to-wild-type ratios of [^18^F]MGX-110S and [^18^F]-GE180 signal using ex vivo autoradiography and (*F*) gamma counting of whole brain. Statistical analyses were performed using unpaired *t* tests or Mann–Whitney tests. *P < 0.05, **P < 0.01, ***P < 0.001. For qPCR: n = 5 to 7 for *Gpr84*, n = 5 to 7 for *Tspo*; for in vivo PET quantification: n = 5 wild-types, n = 7 5xFAD; for autoradiography ratios in the cortex and hippocampus: n = 3 to 5 wild-types, n = 5 5xFAD; for gamma counting ratios, n = 7 to 8 for [^18^F]MGX-110S, n = 7 for [^18^F]GE180. Data are shown as mean ± SD.

Since TSPO-PET remains the most widely used clinical and preclinical approach for imaging neuroinflammation, we compared the performance of GPR84-PET and TSPO-PET in 5xFAD mice. Age- and sex-matched 5xFAD and wild-type mice were injected with [^18^F]GE-180, a sensitive TSPO tracer widely validated in rodents (12, 40), and PET images were acquired 30 to 40 min after tracer administration. Unlike GPR84-PET, which detected significantly elevated tracer binding in the cortex and hippocampus at 3 to 4 mo, TSPO-PET did not detect significant differences in these pathology-rich regions between 5xFAD and wild-type mice at this early stage (Fig. 4*A*). By 7 to 8 mo, TSPO-PET revealed modest but significant increases in cortical and hippocampal binding, consistent with more advanced pathology. These imaging findings were supported by qPCR analyses of whole brain tissue. At 3 to 4 mo, *Gpr84* mRNA levels in 5xFAD mice were 2.46-fold higher than age/sex-matched wild-type controls, whereas *Tspo* expression was increased 1.34-fold relative to controls (Fig. 4*B*). At 7 to 8 mo, expression remained elevated for both genes, with *Gpr84* and *Tspo* levels measuring 2.66-fold and 1.60-fold higher than wild-type controls, respectively. These results demonstrate that GPR84 is elevated earlier and to a greater magnitude than TSPO during disease progression. Consistently, PET quantification of the cortex and hippocampus showed no group differences with TSPO-PET at 3 to 4 mo, while significant increases were detected only at 7 to 8 mo (*P* < 0.05; Fig. 4*C*). Ex vivo autoradiography confirmed these in vivo findings (Fig. 4*D*).

To directly compare the sensitivity of [^18^F]MGX-110S and [^18^F]-GE180 for detecting innate immune activation in the brain, we assessed 5xFAD-to-wild-type binding ratios by ex vivo autoradiography and gamma counting. In 3 to 4-mo-old 5xFAD mice, autoradiography quantification of the cortex and hippocampus showed higher [^18^F]MGX-110S binding ratios relative to [^18^F]GE180 (1.30 vs. 1.02-fold in the cortex; 1.22 vs. 0.94-fold in the hippocampus), with differences reaching statistical significance at 7 to 8 mo (*P* < 0.01; Fig. 4*E*). Gamma counting of perfused whole brains corroborated these results, revealing significantly elevated [^18^F]MGX-110S binding ratios in 5xFAD mice that were 1.77- to 3.21-fold greater than those obtained with [^18^F]GE180 (*P* < 0.01; Fig. 4*F*). Collectively, these data demonstrate that GPR84-PET is more sensitive than TSPO-PET for detecting disease-associated immune activation in 5xFAD mice, enabling earlier detection and more dynamic monitoring of neuroinflammatory progression.

### GPR84-PET Signal Correlates with CD68^+^ Myeloid Cells in 5xFAD Mouse Brains.

**Fig. 5.**
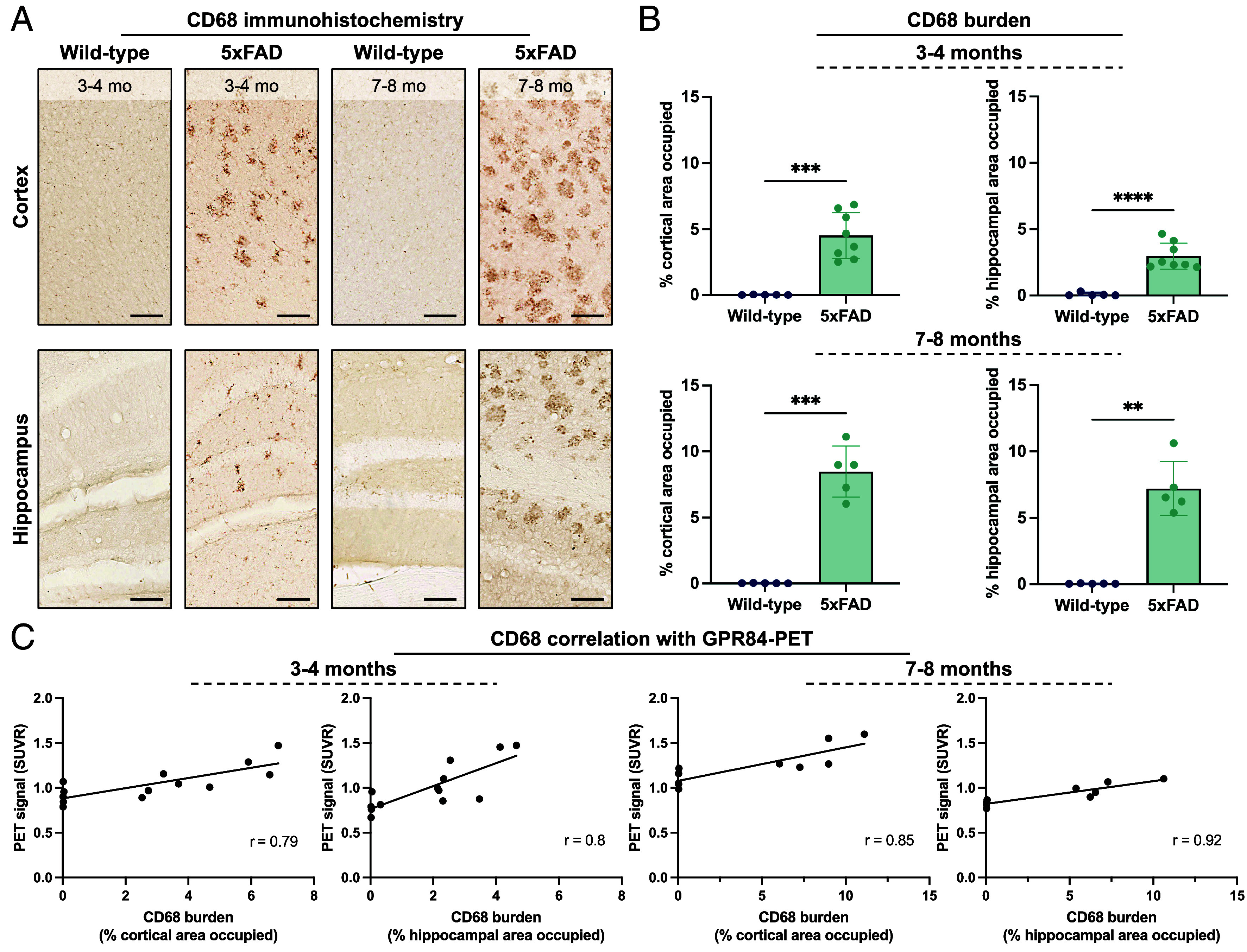
GPR84-PET signal correlates with CD68 immunohistochemistry of activated myeloid cells in 5xFAD mice. (*A*) Representative images at 10× magnification of CD68 immunohistochemical staining in the cortex and hippocampus of wild-type and 5xFAD mice at 3 to 4 and 7 to 8 mo of age. (*B*) Quantification of CD68 burden expressed as the percentage of cortical or hippocampal area occupied by CD68^+^ staining. (*C*) Correlation between CD68 burden and [^18^F]MGX-110S PET signal (SUVR) in the cortex and hippocampus of 3 to 4 and 7 to 8-mo-old mice, demonstrating a strong positive association between PET signal and histological microglial activation. Statistical analyses were performed using unpaired *t* tests (*B*) or simple linear regression (*C*). **P < 0.01, ***P < 0.001, ****P < 0.0001. n = 5 wild-type and n = 5 to 8 5xFAD mice. (Scale bars, 50 µm.) Data are shown as mean ± SD.

To determine whether [^18^F]MGX-110S PET signal reflects myeloid cell activation, we performed CD68 immunohistochemistry on contralateral hemispheres from the same 5xFAD and wild-type brains used for ex vivo autoradiography (Fig. 5*A* and *SI Appendix*, Fig. S8*A*). CD68, a lysosomal glycoprotein expressed by activated microglia and infiltrating macrophages, is a well-established marker of disease-associated myeloid activation in 5xFAD mice (41, 42). Quantitative analysis revealed a significantly larger CD68^+^ area in the cortex, hippocampus, and thalamus of 5xFAD mice compared with wild-type controls at both 3 to 4 mo (*P* < 0.001) and 7 to 8 mo of age (*P* < 0.01; Fig. 5*B* and *SI Appendix*, Fig. S8*B*). The proportion of CD68^+^ activated microglia and macrophages in these brain regions further increased in 7 to 8-mo-old 5xFAD brains relative to 3 to 4-mo-old 5xFAD mice, consistent with progressive myeloid activation during disease advancement. Importantly, regional [^18^F]MGX-110S PET signal correlated with CD68 staining in the cortex (r = 0.79 at 3 to 4 mo; r = 0.85 at 7 to 8 mo), hippocampus (r = 0.8 at 3 to 4 mo; r = 0.92 at 7 to 8 mo; Fig. 5*C*), and thalamus (r = 0.79 at 3 to 4 mo; r = 0.84 at 7 to 8 mo; *SI Appendix*, Fig. S8*C*), validating GPR84-PET as a quantitative method of disease-associated myeloid activation in vivo.

## Discussion

Neuroinflammation, involving sustained myeloid cell activation, is increasingly recognized as a key driver of neurodegenerative disease. In AD, chronic inflammation has emerged as a third core pathological hallmark in addition to Aβ plaques and NFTs (43), linking Aβ deposition to tau pathology and accelerating disease progression. While acute innate immune activation can be transiently protective, prolonged myeloid activation disrupts homeostasis, leading to synaptic loss and neurodegeneration. The development of PET tracers capable of sensitively and specifically detecting this maladaptive immune state in the brain and whole body is therefore critical for disease staging and therapeutic monitoring across neuroinflammatory disorders.

Among explored PET biomarkers, TSPO remains the most widely studied target for clinical imaging of neuroinflammation, but its limited specificity for activated myeloid cells constrains its utility for accurate quantification and tracking of maladaptive immune responses. Alternative biomarkers for innate immune imaging such as CSF1R, CD11b, and P2X7R have been explored, yet many of these suffer from similar limitations, including expression across multiple cell types, high basal expression in healthy tissue with limited differential expression in disease, and/or tracers available for these targets having poor BBB penetration or high nonspecific binding (44, 45). In contrast, GPR84 represents a paradigm shift in neuroimmune imaging: it is a highly inducible, myeloid cell-specific GPCR with minimal basal expression under healthy physiological conditions but is robustly upregulated by proinflammatory mediators such as LPS, TNF, and IL-1β (21, 46–48). This combination of cellular specificity and dynamic transcriptional range positions GPR84-PET as a promising tool for precise mapping of innate immune activation.

In our development of GPR84 tracers, we prioritized three key design criteria: physicochemical properties conducive to CNS penetration, compatibility with ^18^F radiolabeling, and high-affinity binding at nanomolar concentrations. Compared with our prior ^11^C-labeled GPR84 tracers (49), ^18^F offers a longer half-life (t_1/2_ = 109.7 min), enabling off-site production, broader distribution, and scalable solution-based radiochemistry. Guided by structure-activity relationships and CNS MPO scoring, we pursued bioisosteric replacement of –OCH_3_ with –F to directly incorporate ^18^F on the aromatic ring, enhancing metabolic stability while mitigating enzymatic defluorination by cytochrome P450s (50, 51).

To enable rapid access to both ^19^F reference standards and radiochemistry precursors, we developed a concise four-step synthetic pathway to 9-halo dihydropyrimido-isoquinolinones intermediates, reducing prior 10-step synthesis by more than half. This broadly applicable chemistry accommodated halo substituents and permitted structural choreography at the C2 carbonyl, thereby creating a versatile platform for development of other GPR84 tracer and related therapeutic scaffolds (52, 53). By improving synthetic efficiency and reproducibility, our approach is poised to facilitate future medicinal chemistry efforts to refine and diversify GPR84-targeted ligands for imaging and therapeutic applications.

Both manual and automated radiosynthesis of ^18^F-labeled GPR84 tracers were optimized using BPin precursors. Manual radiosynthesis enabled optimization of reaction conditions and tracer identity confirmation, though it suffered from radiation exposure, lower chemical purity, and batch-to-batch variability. Transitioning to automated radiosynthesis on a TRACERLab FX_FN_ module mitigated these constraints, yielding radiotracers with higher RCY and A_m_ while suppressing protodeboronation products commonly encountered in copper(II)-mediated ^18^F-fluorination of BPin scaffolds. Automated production improved radiation safety, reproducibility, and scalability under conditions compatible with current good manufacturing practice (cGMP), establishing a robust platform for both preclinical studies and future clinical translation.

Our lead tracer, [^18^F]MGX-110S, demonstrated high in vitro specificity for GPR84 and enabled detection of innate immune activation in vivo. Compared to established CNS TSPO-PET tracers used in clinical research for measuring neuroinflammation such as [^18^F]DPA-714 and [^18^F]FEPPA, [^18^F]MGX-110S exhibited more favorable brain uptake (Brain_1min/60min_ ≈ 3.7) and in vivo stability. Both [^18^F]DPA-714 and [^18^F]FEPPA display moderate peak uptake and high baseline profile (Brain_1min/60min_ ≈ 1 and 1.7, respectively), with only 54% and 85% intact tracer remaining in rodent brain at 60 min postinjection (54, 55). Given interspecies differences in tracer metabolism, clinical evaluation of [^18^F]MGX-110S is warranted to validate its metabolic profile and determine whether arterial sampling will be necessary for kinetic modeling.

To evaluate in vivo performance, we examined tracer binding in an LPS-induced systemic- and neuro-inflammation model. LPS administration is known to induce widespread microglial activation, neuronal injury, and release of neurotoxic mediators such as TNF, interleukin-1β (IL-1β), and prostaglandin E2 (PGE_2_) (56, 57). The increased binding of [^18^F]MGX-110S in brain and immune cell-rich peripheral tissues including the spleen, liver, and adipose is consistent with prior evidence of colocalization of GPR84 mRNA expression with Iba1—a canonical marker of activated microglia and peripheral macrophages—and corresponds with previous findings in LPS-induced mice demonstrating GPR84 as a marker of activated innate immune cells during systemic and CNS inflammation (21).

While other tracers such as those targeting CD11b and CSF1R have been developed to advance our understanding of innate immune processes, their clinical utility for imaging *neuro*inflammation is yet to be fully determined and have some key limitations. Available CD11b-targeted agents rely on antibody-based constructs or peptide ligands with poor BBB permeability, slow pharmacokinetics, and higher barrier to clinical translation, restricting their suitability for imaging rapidly evolving neuroimmune responses (58). In contrast, CSF1R-targeted tracers, including several ^11^C and ^18^F-labeled small molecules, can visualize microglial proliferation but cannot distinguish homeostatic from proinflammatory myeloid phenotypes and exhibit high levels of off-target binding (59, 60). Transcriptomic profiling further underscores these limitations: in 7-mo-old 5xFAD mice, *Csf1r* expression increased less than twofold relative to wild-type controls, whereas *Gpr84* rose more than fivefold (61). Similarly, in human multiomics datasets spanning 88 AD cohorts, GPR84 showed the strongest differential expression between AD and control groups among 408 GPCRs surveyed (62). Recent work by Cox et al. highlights the important functional role of GPR84 in driving proinflammatory signaling that impairs hippocampal function and accelerates cognitive decline in aged C57BL/6 mice (63). Therefore, taken together, both human and preclinical data indicate that GPR84 is a promising imaging target that plays a significant role in AD and age-associated cognitive decline.

When compared directly with TSPO-PET, which is currently the most widely used PET biomarker for neuroinflammation in clinical research, GPR84-PET demonstrated higher sensitivity in detecting microglial activation at in 3 to 4-mo-old 5xFAD mice, an age corresponding to the early emergence of amyloid pathology and the onset of microgliosis in this model. By 7 to 8 mo, as amyloid pathology and neuroinflammation became more widespread—extending across the cortex and hippocampus and into subcortical regions—tracer binding differences between 5xFAD and wild-type mice further amplified, closely mirroring the known spatiotemporal progression of amyloid pathology in this model (64, 65). Although rodent models cannot fully recapitulate the complexity and clinical characteristics of human AD, these findings highlight the ability of GPR84-PET to capture the spatiotemporal evolution of neuroinflammation with greater specificity and dynamic range than existing tracers.

Prior studies have established CD68 as a quantitative marker of amyloid phagocytosis in 5xFAD mice and their colocalization with Aβ within microglial phagolysosomes (41, 42). In this study, we showed correlation between [^18^F]MGX-110S binding and CD68^+^ staining, providing histological validation that GPR84-PET signal reflects the abundance of plaque-associated, activated myeloid cells in vivo. Notably, even at early disease stages when amyloid deposition and microgliosis are just emerging, GPR84-PET correlated with CD68, underscoring its sensitivity for detecting neuroinflammation prior to overt plaque burden. This capacity for early detection is particularly valuable for translational application, enabling identification of at-risk individuals, disease staging, therapeutic monitoring of existing and novel immunomodulatory therapies, and advancement of drug development for neurodegenerative and neuroinflammatory diseases.

In conclusion, we developed a streamlined synthetic strategy to access previously unreported bromo- and fluoro-substituted dihydropyrimido-isoquinolinones, enabling efficient synthesis of GPR84-PET tracers. Optimized manual and automated radiosynthesis produced chemically and radiologically pure tracers with high molar activity while minimizing protodeboronation byproducts that typically compromise yield, molar activity, and clinical translatability. [^18^F]MGX-110S demonstrated high specificity for GPR84 and enabled whole-body visualization of innate immune activation, showing superior sensitivity for early-stage neuroinflammation compared with TSPO-PET in 5xFAD mice. These findings establish GPR84-PET as a highly promising imaging approach with potential broad clinical applications, from accurate disease staging and therapeutic monitoring in AD to mechanistic studies of immune dysregulation in multiple sclerosis, Parkinson’s disease, and other neuroinflammatory and neurodegenerative diseases. Ongoing IND-enabling studies—including toxicology and clinical validation radiochemistry runs—are positioning this tracer for first-in-human evaluation.

## Materials and Methods

### Animals.

All animal studies were performed in female C57BL/6J mice (the Jackson Laboratory). 5xFAD mice [strain B6.Cg-Tg(APPSwFlLon,PSEN1*M146L*L286V)6799Vas/Mmjax, RRID:MMRRC_034848-JAX] were obtained from the Mutant Mouse Resource and Research Center (MMRRC) at The Jackson Laboratory, donated by Robert Vassar, Ph.D. (Northwestern University). All procedures were approved by the Stanford Administrative Panel on Laboratory Animal Care (APLAC), accredited by the Association for the Assessment and Accreditation of Laboratory Animal Care (AAALAC International). All federal and state regulations governing the humane care and use of laboratory animals were upheld.

### Precursors and Cold Standards Synthesis.

Briefly, 3-halo (fluoro or bromo phenyl) ethylamine was first N-acetylated, followed by polyphosphoric acid-induced cyclization to yield 6-halo-1-methyl-3,4-dihydroisoquinoline intermediates (**5** and **6**, Scheme 1). Ring closure with benzoyl isocyanate and excess triethylamine in toluene (80 °C, 16 h) produced 9-halo-6,7-dihydropyrimido[6,1-*a*]isoquinoline-2,4-dione derivatives (**7** and **8**). Reaction with POCl_3_ (65 °C, 3 d) produced 9-halo-2-chloro-6,7-dihydropyrimido[6,1-*a*]isoquinolin-4-one intermediates (**9** and **10**). The 9-bromo intermediate (**9**) was used to synthesize MGX-90S and MGX-100S precursors via i) coupling with (R)-dioxane alcohol in the presence of potassium *tert*-butoxide (RT, 1 h) and ii) Pd-catalyzed borylation with bis(pinacolato)diboron. Reference standards MGX-110S and MGX-111S were synthesized by substitution of the chloro group of the 9-fluoro intermediate (**10**) with (*R*) dioxane alcohol (details are provided in *SI Appendix*).

### [^18^F]MGX-110S and [^18^F]MGX-111S Radiosynthesis.

Radiolabeling conditions were optimized using KOTf/Et_4_NH_4_HCO_3_ and Kryptofix methods across multiple solvents (DMA/butanol and DMF) and temperatures (*SI Appendix*, Tables S2–S4). Optimized conditions were implemented for automated synthesis of [^18^F]MGX-110S and [^18^F]MGX-111S on a GE TRACERlab FX_FN_ module. [^18^F]Fluoride was trapped on an QMA-light cartridge, eluted with 0.1 M KOTf (5 mg in 495 µL) and tetraethylammonium bicarbonate (0.5 mg in 10 µL), and dried azeotropically with 1 mL acetonitrile at 110 °C. The precursor (MGX-90S or MGX-100S, 2 mg, 1 eq) was reacted with Cu(OTf)_2_ (8.2 mg, 5 eq) and pyridine (44 µL, 125 eq) in DMA/1-butanol (2/1, v/v, 250 μL) at 130 °C for 18 min. The crude mixture was purified by semipreparative HPLC (Gemini C18 column) using isocratic elution (H_2_O/MeCN + 0.1% TFA, 70:30) at 4 mL/min. The radioactive peak corresponding to [^18^F]MGX-110S [retention time (R_t_) = 17 to 19 min] or [^18^F]MGX-111S (R_t_ = 22.5 to 25 min) was collected, trapped on a C18 cartridge, eluted with ethanol, diluted with saline, and formulated for injection. Product identity was confirmed by analytical HPLC coinjection with nonradioactive standards. Molar activity was determined from the UV absorbance (254 nm) relative to calibration curves (see *SI Appendix* for details).

### [^18^F]GE180 Radiosynthesis.

[^18^F]GE180 was synthesized as previously described (66) and formulated in ethanol/saline (10:90, v/v).

### Cell Culture.

Human GPR84^+^ HEK293 cells and parental HEK293 controls (Creative Biogene, Shirley, NY) were cultured in Dulbecco’s modified Eagle’s medium (DMEM; Thermo Fisher Scientific) with 10% fetal bovine serum (FBS; Thermo Fisher Scientific) and 1% antibiotic-antimycotic (Thermo Fisher Scientific). For hGPR84^+^ HEK293 cells, puromycin (1 µg/mL; Thermo Fisher Scientific) was added to maintain stable selection. Cells were cultured at 37 °C in a humidified incubator with 5% CO_2_ and routinely tested for *Mycoplasma* contamination and confirmed negative prior to all binding studies.

hMDMs were isolated from whole blood (Stanford Blood Center, Stanford, CA) as previously described (67) and resuspended at 1 × 10^6^ cells/mL in serum-free RPMI 1640 media with 1% penicillin-streptomycin (pen/strep; Thermo Fisher Scientific), 10% FBS (Thermo Fisher Scientific), and 50 ng/mL macrophage colony-stimulating factor (M-CSF; Avantor, Radnor, PA).

### Cell Binding Studies.

Cells were seeded at 3 × 10^5^ cells/mL in 12-well plates (Corning, Glendale, AZ) ~24 h before experiments. For tracer binding studies, hGPR84^+^ HEK293 and parental HEK293 cells were incubated with prewarmed DMEM (without FBS or antibiotics) containing ~0.925 MBq (25 µCi/mL) of [^18^F]MGX-110S or [^18^F]MGX-111S (1 mL/well; n = 8/group). For blocking experiments, tracer was coincubated with GLPG1205 (35 µM). For hMDM studies, cells were stimulated with LPS (100 ng/mL) or saline 24 h prior to incubation with [^18^F]MGX-110S (~0.925 MBq/mL; n = 4/donor/group), with or without GLPG1205 (35 µM). After 40 min at 37 °C, media were aspirated, and cells were washed twice with ice-cold phosphate-buffered saline (PBS; 1 mL), trypsinized with 150 µL of Trypsin-EDTA, and neutralized with 600 µL DMEM containing 10% FBS and 1% antibiotic-antimycotic. Aliquots (500 µL) were transferred to gamma-counting tubes, and activity was measured on a 2470 Wizard2 gamma counter (Revvity, Inc., Waltham, MA). Radiotracer standards (3 × 500 µL) were counted in parallel for decay correction and normalization.

### Saturation Binding Studies.

To determine dissociation constants (K_D_), hGPR84^+^ HEK293 cells (1 × 10^6^ cells/well) were incubated with increasing concentrations of [^18^F]MGX-110S or [^18^F]MGX-111S (0.000148-37 MBq; 0.004 to 1,000 µCi/mL; n = 4/concentration, 10 concentrations total). Nonspecific binding was determined by coincubation with a 1,000-fold excess GLPG1205. After 1 h at 37 °C, media were aspirated, cells were trypsinized, then washed twice with ice-cold PBS, and gamma counted as described above. Tracer concentrations were calculated from specific activity and administered dose. Specific binding was calculated as total minus nonspecific binding and normalized to CPM per cell. K_D_ values were obtained by nonlinear regression (one-site specific binding model) using GraphPad Prism v10 (GraphPad Software, Boston, MA).

### Competitive Binding Studies.

To determine half-maximal inhibitory concentration (IC_50_), hGPR84^+^ HEK293 cells (1 × 10^6^ cells) were incubated with GLPG1205 (10^−3^–10^−12^ M) and [^18^F]MGX-110S or [^18^F]MGX-111S (1 mL per well; n = 4 per concentration) for 1 h at 37 °C. Cells were then trypsinized, washed twice with ice-cold PBS, and gamma counted as described above. IC_50_ values were determined by nonlinear regression (log inhibitor vs. response, variable slope; one-site fit logIC_50_) using GraphPad Prism v10.

### Radiometabolite Analysis.

Mice received LPS (5 mg/kg, i.p.) ~24 h before tracer injection. Purified [^18^F]MGX-110S (40.7 ± 1.55 MBq), verified by analytical HPLC, was administered via tail vein under anesthesia. Blood (~500 µL) was collected by cardiac puncture at 30 and 60 min postinjection into heparinized tubes and centrifuged at (1,800 g, 4 min, RT) to isolate plasma. Plasma (200 µL) was mixed with ice-cold acetonitrile (300 µL) to precipitate proteins. Following blood collection, mice were perfused with PBS (20 mL). Whole brain and a representative liver lobe were harvested, homogenized in acetonitrile (500 µL), and centrifuged (9,400 g, 4 min, RT). Supernatants and pellets were separated for gamma counting to determine extraction efficiency. For radiometabolite analysis, supernatants (120 µL) were injected into HPLC vials and analyzed by radio-HPLC using a Phenomenex Gemini C18 column (5 µm, 110 Å, 250 × 4.6 mm).

### PET Imaging and Analysis.

Mice were anesthetized with isoflurane gas and injected intravenously with either [^18^F]MGX-110S or [^18^F]GE-180 (5.55 MBq). For LPS studies, 60-min dynamic PET scans were initiated immediately prior to tracer injection. For AD studies, 10-min static PET scans were acquired 30 to 40 min postinjection. PET imaging was performed on a GNEXT scanner (Sofie Biosciences, Dulles, VA) in list-mode format, followed by CT scans for anatomical reference and attenuation/scatter correction. Images were reconstructed using OSEM3D/MAP (24 subsets, three iterations; matrix size 240 × 240 × 191). Whole-brain PET quantification was performed by fitting a 3D mouse brain atlas using Vivoquant 2022 (Perceptive Inc., London, UK), as previously described (68). PET images were visualized using Inveon Research Workspace (version 4.0, Siemens, Munich, Germany). Analysis rigor is described in *SI Appendix* (E. Supplementary Methods section under “PET imaging analysis”).

### Ex Vivo Gamma Counting and Autoradiography.

Following PET imaging, tissues were harvested for ex vivo biodistribution and autoradiography. Organs of interest (heart, lungs, liver, spleen, kidney, muscle, bone, bone marrow, adipose, brain, and tail) were dissected, weighed wet, and measured using an automatic gamma counter (Hidex, Turku, Finland). Brain tissue was further analyzed by digital autoradiography using an Amersham Typhoon phosphorimager (Cytiva, Marlborough, MA). The same sections were subsequently stained with Nissl (cresyl violet acetate, Sigma-Aldrich, St. Louis, MO) for anatomical reference. Autoradiography images were visualized using ImageJ software (version 1.53t). Organs with incomplete perfusion (e.g., visibly pink tissue indicating residual blood) were excluded.

### Flow Cytometry.

LPS and vehicle-treated mice were perfused with PBS and killed humanely under isoflurane anesthesia. Brains were harvested and mechanically homogenized in ice-cold CNS buffer (2.5% HEPES in HBSS without Ca/Mg; Gibco) and resuspended in FACS buffer (2% FBS [Thermo Fisher Scientific, #A5670701]). Antibody details are provided in SI Materials and Methods. Cells were washed and fixed with 2% paraformaldehyde (PFA, Thermo Fisher Scientific, J19943.K2) for 20 min at room temperature. Flow data were analyzed on the Cytek Aurora (Cytek Biosciences, Fremont, CA). FlowJo software (v.10.10.0, BD Biosciences) was used for the analysis and depiction of the gating strategy.

### RNA Extraction, cDNA Synthesis, and qRT-PCR.

Following gamma counting, brain tissues were snap-frozen in TRIzol (Thermo Fisher Scientific, Waltham, MA) on dry ice and stored at −80 °C. RNA was isolated using the manufacturer’s TRIzol RNA extraction protocol (Thermo Fisher Scientific) and complementary DNA was synthesized using the RT^2^ First Strand Kit (Qiagen, Redwood City, CA). qPCR was performed using primers targeting *Gpr84* (Qiagen) and *Tspo* (Qiagen), with *Gapdh* as the housekeeping control. Reactions were run on an Applied Biosystems QuantStudio 6 system (Thermo Fisher Scientific). All samples were analyzed in triplicate, and relative expression was calculated using 2^−ΔΔCT^.

### Immunohistochemistry.

Brains were fixed in 4% PFA (Thermo Fisher Scientific) for 24 h, followed by 30% sucrose (Thermo Fisher Scientific) at 4 °C and embedded in optimal cutting temperature (OCT) compound (Sakura Finetek USA, Torrance, CA). Coronal sections (50 μm) were prepared, rinsed in 1× PBS, and stored at −20 °C. Sections were incubated overnight at 4 °C with rat anti-mouse CD68 primary antibody (1:1,000) (MCA1957, Bio-Rad Laboratories, Hercules, CA). The following day, sections were incubated with biotinylated anti-rat secondary antibody (1:1,000) (BA-4001, Vector Laboratories, Newark, CA) for 45 min. Staining was visualized using the peroxidase substrate 3,3’-diaminobenzidine tetrahydrochloride hydrate (DAB; #D5637, Sigma-Aldrich, St. Louis, MO).

### Quantitation of CD68 Staining.

CD68-positive microglia/macrophages were quantified in two coronal brain sections per mouse from both the 3 to 4-mo group (n = 5 wild-type; n = 8 5xFAD) and the 7 to 8-mo group (n = 5 wild-type; n = 5 5xFAD). For each section, two 20× fields were sampled from the primary and secondary somatosensory cortices and two additional 20× fields from the hippocampus (between bregma +1.10 and −1.82), yielding a total of four fields per region per mouse. Images were analyzed using ImageJ (version 1.53t) with standardized thresholding parameters applied across all samples.

### Statistical Analysis.

Statistical analyses of in vivo PET, ex vivo gamma counting and autoradiography, and qPCR were performed using GraphPad Prism (version 10). Statistical analyses were performed using two-tailed unpaired *t* tests or one- or two-way ANOVA with multiple comparisons or nonparametric equivalents as indicated in the figure legends for each dataset.

## Supplementary Material

Appendix 01 (PDF)

## Data Availability

PET tracers generated in this study can be provided by M.L.J. under a Stanford University material transfer agreement. Other data are included in the article and/or *SI Appendix*.
