# Supplementary material for: Illuminating proinflammatory myeloid cells with PET tracers targeting GPR84
Source: Proc Natl Acad Sci U S A. 2026 May 21;123(21):e2536372123. doi: 10.1073/pnas.2536372123 (PMC13214000; doi:10.1073/pnas.2536372123)

SUPPORTING INFORMATION

Illuminating proinflammatory myeloid cells with PET tracers targeting GPR84

Mausam Kalita[a,1], Renesmee C. Kuo[a,b,1], Valentina Straniero[c], Samantha Reyes[a], Mallesh Pandrala[a], Alessia Lanzini[c], Sara Marsango[d], Desiree D'Moore[a], Piper Mahn[a], Andrew Setiadi[a], Mira Sundar[a], Spencer Mak[a], Sydney Nagy[a], Israt S. Alam[a], Poorva Jain[a], Grace Inay[a], Rim Malek[a], Allen F. Brooks[e], Corinne Beinat[a], Ermanno Valoti[c], Peter J. H. Scott[e], Graeme Milligan[d], Michelle L. James[a,f,*]

[a]Department of Radiology, Stanford University, Stanford, CA, USA, [b]Department of Electrical Engineering, Stanford University, Stanford, CA, USA, [c]Department of Pharmaceutical Sciences, University of Milan, Via Luigi Mangiagalli 25, Milano, Italy, [d]Centre for Translational Pharmacology, School of Molecular Biosciences, College of Medical, Veterinary and Life Sciences, University of Glasgow, Glasgow, Scotland, U.K., [e]Division of Nuclear Medicine, Department of Radiology, University of Michigan Medical School, Ann Arbor, Michigan, USA, [f]Department of Neurology and Neurological Sciences, Stanford University, Stanford, California, USA

[1]These authors contributed equally to this work

[*]Corresponding author. 3172 Porter Drive, Room 273, Palo Alto, CA, USA; Tel: 650-497-0153;

Email: mljames@stanford.edu

Table of Contents

A. Materials 3

B. Synthetic Procedures 4

C. General Procedure for Manual Radiochemistry 16

D. Automation of [^{18}F]MGX-110S and [^{18}F]MGX-111S Radiotracers 18

E. Supplementary Methods 18

F. Supplementary Figures 20

G. Supplementary Tables 28

H. ^{1}H, ^{19}F and ^{13}C NMR Spectra and High-Resolution Mass Spectrometry 31

A. Materials

All reactions were done under dry and nitrogen atmosphere unless mentioned otherwise. Anhydrous chemicals and solvents were purchased from commercial suppliers (Sigma-Aldrich, Ambeed, Fluorochem, Merck and TCI) and used without further purifications. 2-(hexylthio)-6-hydroxy-4(3H)-pyrimidinone (2-HTP) was from Sigma-Aldrich (Dorset, UK). 9-cyclopropylethynyl-2-((S)-1-[1,4]dioxan-2-ylmethoxy)-6,7-dihydropyrimido[6,1-a]isoquinolin-4-one (GLPG1205) was from MedChemExpress (Sollentuna, Sweden). [^{35}S]GTPγS was from PerkinElmer Life Sciences (Beaconsfield, UK). [^{3}H]9543 was provided by Laurent Saniere (Galapagos NV). Lipopolysaccharide from Escherichia coli O55:B5 (L2880-25 mg, Lot 0000189843, Source 0000174045) was purchased from Millipore Sigma. Tissue culture reagents were from Thermo Fisher Scientific (Loughborough, UK). Column chromatography was done with manual column chromatography using SilicaFlash R60 gel (20-45 µm, SiliCycle Inc.). Thin layer chromatography (TLC) was conducted on pre-coated polyester sheets (40 x 80 mm) from Machery-Nagel (POLYGRAM® SIL G/UV254) with 0.2 mm silica gel 60 with fluorescent indicator. For visualization, a UV light source (254 nm and 366 nm) was used. For **compound 3** to **compound 8**, the nuclear magnetic resonance (NMR) spectra were measured by Varian Mercury 300 NMR spectrometer/Oxford Narrow Bore superconducting magnet operating at 300 MHz (^{1}H-NMR). The ^{13}C-NMR spectra were acquired operating at 75 MHz. The ^{19}F-NMR spectra were acquired operating at 282 MHz. For all other compounds, the NMR spectra were recorded on a Varian 400 MHz NMR spectrometer equipped with a (^{1}H/^{19}F/^{13}C) probe head. Chemical shifts (δ) are given in ppm, coupling constants J in Hertz (Hz) and the multiplicities of the signals are designated as follows: s = singlet, bs = broad singlet, d = doublet, t = triplet, and m = multiplet. The solvent signal of $CDCl_3$ and CD_3OD, and DMSO-d_6 were calibrated on 7.26 ppm, 3.34 ppm, and 2.5 ppm for ^{1}H-NMR and 77.0 ppm, 49.15 ppm, and 39.52 ppm for ^{13}C-NMR spectra. For **compound 3** to **compound 8**, high resolution mass spectrometry (HRMS) spectra were acquired using a Q-tof SYNAPT G2-Si HDMS 8K (Waters) coupled with an electrospray ionization (ESI) source in positive (ES+) ion mode. HRMS analyses were performed at the Mass Spectrometry facility of the Unitech COSPECT at the University of Milan (Italy). Stanford University Mass Spectrometry (SUMS) Facility recorded high-resolution mass spectra (HRMS) for all other compounds.

B. Synthetic Procedures

N-(3-halo-phenethyl) acetamide: Compound 3 (X = Br) and Compound 4 (X = F)

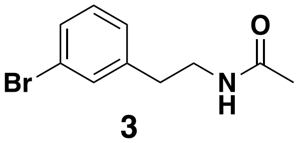

3

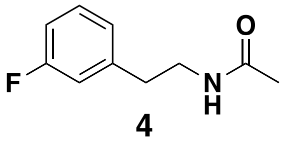

4

TEA (8.36 mL, 59.98 mmol) was added to a solution of 2-(3-halo-phenyl)ethylamine (10 g (X = Br) or 7g (X = F), 49.98 mmol) in DCM (100 mL) at 0 °C. Then, acetic anhydride (5.67 mL, 59.98 mmol) was added dropwise, always keeping the reaction mixture at 0°C. Once added, the reaction was allowed to room temperature and stirred at room temperature for 3 hours. After completion, the reaction was first washed with 10 % aqueous HCl followed by 10% aqueous NaCl. The organic phase was dried over Na_2SO_4, filtered, and concentrated under vacuum, affording quantitative yield of *N*-(3-bromophenethyl)acetamide (3) or *N*-(3-fluorophenethyl)acetamide (4), both as yellowish oils.

Compound 3

^{1}H-NMR δ (300 MHz, $CDCl_3$): 7.36 (m, 2H), 7.18 (dt, *J* = 7.6, 0.6 Hz, 1H), 7.12 (m, 1H), 5.54 (bs, 1H), 3.49 (dt, *J* = 7.0, 6.9 Hz, 2H), 2.79 (t, *J* = 7.0 Hz, 2H), 1.95 ppm (s, 3H).

^{13}C-NMR δ (75 MHz, $CDCl_3$): 170.2, 141.2, 131.8, 130.2, 129.7, 127.4, 122.6, 40.5, 35.3, 23.3.

HRMS (ESI): m/z calculated for $C_{10}H_{12}NONaBr$ [Na-adduct], 264.0000, found: 263.9993

Compound 4

^{1}H-NMR δ (300 MHz, $CDCl_3$): 7.28 (m, 1H), 6.98-6.88 (m, 3H), 5.55 (bs, 1H), 3.51 (dt, *J* = 7.0, 6.6 Hz, 2H), 2.82 (t, *J* = 7.0 Hz, 2H), 1.95 ppm (s, 3H).

^{13}C-NMR δ (75 MHz, $CDCl_3$): 170.3, 162.9 (d, *J* = 246.0 Hz), 141.5 (d, *J* = 7.2 Hz), 130.0 (d, *J* = 8.3 Hz), 124.4 (d, *J* = 2.7 Hz), 115.5 (d, *J* = 20.9 Hz), 113.4 (d, *J* = 21.0 Hz), 40.5, 35.4, 23.2.

^{19}F NMR δ (282 MHz, $CDCl_3$): -113.21 (dt, *J* = 6.0, 9.4 Hz).

HRMS (ESI): m/z calculated for $C_{10}H_{12}NONaF$ [Na-adduct], 204.0801, found: 204.0794

6-halo-1-methyl-3,4-dihydroisoquinoline: Compound 5 (X = Br) and Compound 6 (X = F)

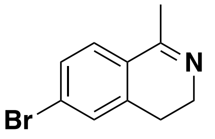

5

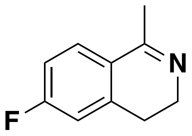

6

N-(3-bromo-phenethyl)acetamide (12.0 g, 49.77 mmol) or *N*-(3-fluorophenethyl)acetamide (9.0 g, 49.66 mmol) was treated with polyphosphoric acid [48 g (X = Br) or 36 g (X = F)] and heated to 200 °C for 4 hours in a sealed tube. The reaction mixture was then poured into stirred ice-cold water (300 mL); pH adjusted to 10 using 30% aqueous NH_4OH and extracted with ethyl acetate (3 x 100 mL). The collected organic phases were dried over Na_2SO_4, filtered, and concentrated under vacuum to give a residue, which was purified by flash chromatography on silica gel, eluted with ethyl acetate and giving 6-bromo-1-methyl-3,4-dihydroisoquinoline or 6-fluoro-1-methyl-3,4-dihydroisoquinoline as yellowish oils.

Compound 5

Yield amount = 6.71 g, Percentage yield: 60%

^{1}H-NMR δ (300 MHz, $CDCl_3$): 7.38 (m, 3H), 3.65 (t, *J = 7.5 Hz*, 2H), 2.67 (t, *J* = 7.5 Hz, 2H), 2.36 ppm (s, 3H).

^{13}C-NMR δ (75 MHz, $CDCl_3$): 163.4, 139.4, 130.4, 130.0, 128.2, 126.8, 124.6, 46.6, 25.8, 23.1.

HRMS (ESI): m/z calculated for $C_{10}H_{11}NBr$, 224.0075, found: 224.0073

Compound 6

Yield amount = 2.6 g, Percentage yield: 52%

^{1}H-NMR δ (300 MHz, $CDCl_3$): 7.46 (dd, *J* = 8.2, 5.8 Hz, 1H), 6.96 (m, 1H), 6.89 (dd, *J* = 8.2 Hz, 1H), 3.65 (t, *J* = 7.3 *Hz*, 2H), 2.70 (t, *J* = 7.3 Hz, 2H), 2.37 ppm (s, 3H).

^{13}C-NMR δ (75 MHz, $CDCl_3$): 163.4 (d, *J* = 251.3 Hz), 163.0, 140.2 (d, *J* = 8.5 Hz), 127.3 (d, *J* = 8.9 Hz), 125.8 (d, *J* = 3.0 Hz), 114.2 (d, *J* = 21.6 Hz), 113.4 (d, *J* = 21.5 Hz), 46.4, 26.1, 23.1.

^{19}F NMR δ (282 MHz, $CDCl_3$): -109.20 (dt, *J* = 5.6, 8.8 Hz)

HRMS (ESI): m/z calculated for $C_{10}H_{11}NF$, 164.0876, found: 164.0871

Benzoyl isocyanate

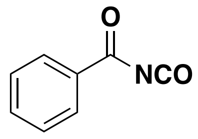

benzoyl isocyanate

B

Oxalyl chloride (29 mL) was slowly added to commercial benzamide (7.24 g, 59.80 mmol) under nitrogen atmosphere, at room temperature. The suspension was then heated at 50 °C and stirred for 2 hours. Next, the solution was concentrated *in loco* under vacuum to give 8.80 g (quantitative yield) of benzoyl isocyanate as a yellowish oil, which was directly used without further purification.

^{1}H-NMR δ (300 MHz, $CDCl_3$): 8.07 (d, *J* = 7.3 Hz, 2H), 7.65 (t, *J* = 7.4 Hz, 1H), 7.49 ppm (m, 2H)

9-halo-6,7-dihydropyrimido[6,1-*a*]isoquinoline-2,4-dione: Compound 7 (X = Br) and Compound 8 (X = F)

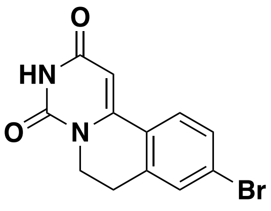

7

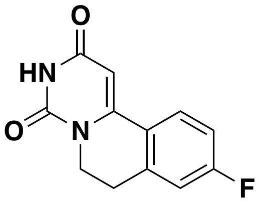

8

6-bromo-1-methyl-3,4-dihydroisoquinoline (compound **5**, 3.35 g, 14.95 mmol) (or 6-fluoro-1-methyl-3,4-dihydroisoquinoline [compound **6**, 2.58 g, 15.82 mmol)] and TEA (8.33 mL, 59.80 mmol (X = Br) or [8.82 mL, 63.28 mmol (X = F)] were sequentially added slowly to a solution of freshly prepared benzoyl isocyanate (8.80 g, 59.80 mmol) in toluene at 5-10 °C. The solution was warmed to RT and then heated and stirred at 80 °C overnight. After completion, the resulting suspension was filtered. For work up, the crude solid was stirred vigorously in water for 30 min at room temperature. Water was decanted, and solid was refluxed in methanol for 30 min. Lastly, the resulting solid was filtered through a frit funnel and dried under vacuum.

Compound 7

Compound color = white, Yield amount = 2.5 g, Percentage yield: 57%

^{1}H-NMR δ (300 MHz, DMSO-d_6): 11.35 (bs 1H), 7.86 (d, *J* = 6.0 Hz, 1H), 7.65 (s, 1H), 7.54 (d, *J* = 6.0 Hz, 1H), 6.21 (s, 1H), 3.90 (t, *J* = 7.0 Hz, 2H), 2.97 ppm (t, *J* = 7.0 Hz, 2H)

^{13}C-NMR δ (75 MHz, DMSO-d_6): 163.3, 151.4, 148.3, 139.4, 131.04, 130.8, 128.8, 126.9, 125.4, 97.2, 39.3, 27.3 ppm

HRMS (ESI) m/z calculated for $C_{12}H_9N_2O_2NaBr$ [Na-adduct], 314.9745, found: 314.9745

Compound 8

Compound color = yellow, Yield amount = 1.8 g, Percentage yield: 50%

^{1}H-NMR δ (300 MHz, DMSO-d$_6$): 11.32 (bs 1H), 7.98 (dd, *J* = 8.8, 5.5 Hz, 1H), 7.26 (dd, *J* = 8.8, 2.7 Hz, 1H), 7.18 (m, 1H), 6.17 (s, 1H), 3.91 (t, *J* = 6.2 Hz, 2H), 2.98 ppm (t, *J* = 6.2 Hz, 2H)

^{13}C-NMR δ (75 MHz, DMSO-d$_6$): 164.0 (d, *J* = 250.4 Hz), 163.4, 151.5, 148.4, 140.3 (d, *J* = 9.1 Hz), 129.7 (d, *J*=9.4 Hz), 124.1 (d, *J* = 2.8 Hz), 115.4 (d, *J* = 15.1 Hz), 115.1 (d, *J* = 15.3 Hz), 96.7, 39.4, 27.6 ppm

^{19}F NMR δ (282 MHz, DMSO-d$_6$): δ -108.87, -108.88, -108.90, -108.90, -108.92, -108.93, -108.94, -108.95 (ddd, *J* = 9.2, 7.4, 4.4 Hz, 1F)

HRMS (ESI): m/z calculated for $C_{12}H_9N_2O_2NaF$ [Na-adduct], 255.0546, found: 255.0542

9-halo-2-chloro-6,7-dihydropyrimido[6,1-*a*]isoquinolin-4-one [Compound 9 (X = Br) and Compound 10 (X = F)]

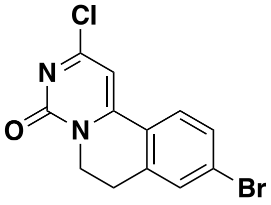

9

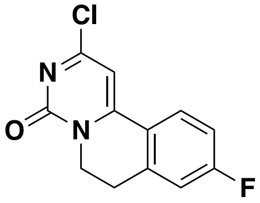

10

9-bromo-6,7-dihydropyrimido[6,1-*a*]isoquinoline-2,4-dione (compound **7**, 1 equivalent) or 9-fluoro-6,7-dihydropyrimido[6,1-*a*]isoquinoline-2,4-dione (compound **8**, 1 equivalent) was placed into a 250 mL round bottom flask (RBF) equipped with a stir bar and subjected to three cycles of vacuum and nitrogen flush. Subsequently, 30 mL $POCl_3$ was added to the RBF and stirred at 65 °C for 3 days. The reaction was cooled to room temperature and directly poured into ice cold water (300 mL) inside a 1L conical flask, which was kept in an ice bath. The pH of this mixture was brought to 7-8 by *slowly* adding sodium carbonate in the solid form while stirring (producing a foam/froth-like solution). Once a pH of 7-8 was reached, the yellow precipitate was filtered through a frit, washed with cold water (1000 mL) while stirring, and the precipitate manually removed with a glass rod. The compound was then dried under vacuum.

Compound 9

Compound color = pale yellow, Percentage yield: 82.15%

^{1}H NMR (400 MHz, DMSO-*d$_6$*): δ 8.01 (d, *J* = 8.4 Hz, 1H), 7.81 – 7.57 (m, 2H), 7.26 (s, 1H), 4.06 (t, *J* = 6.5 Hz, 2H), 3.19 – 2.94 (m, 2H)

^{13}C NMR (400 MHz, DMSO-*d$_6$*): δ 164.17, 153.84, 153.24, 139.44, 131.09, 130.56, 129.05, 126.79, 125.33, 100.04, 40.24, 25.92

HRMS (ESI): m/z calculated for $C_{12}H_8BrClN_2O$ $[M+H]^+$ 310.9578, found 310.9573

Compound 10

Compound color = yellow, Percentage yield: 78.32%

^{1}H NMR (400 MHz, Chloroform-*d*): δ 7.77 (dd, *J* = 8.8, 5.2 Hz, 1H), 7.19 – 7.02 (m, 2H), 6.72 (s, 1H), 4.31 – 4.19 (m, 2H), 3.07 (t, *J* = 6.6 Hz, 2H)

^{13}C NMR (400 MHz, Chloroform-*d*): δ 163.34, 151.47, 148.34, 140.31, 140.22, 129.71, 129.62, 124.14, 115.47, 115.25, 115.04, 96.75, 27.61

^{19}F NMR (400 MHz, Chloroform-*d*) δ -104.06, -104.07, -104.08, -104.09, -104.10, -104.11 (td, *J* = 8.2, 5.2 Hz, 1F)

HRMS (ESI): m/z calculated for $C_{12}H_8ClFN_2O$ $[M+H]^+$ 251.0378, found 251.0389

9-halo-2-[[(2S)-1,4-dioxan-2-yl]methoxy]-6,7-dihydropyrimido[6,1-*a*]isoquinolin-4-one [Compound 11 (X = Br), MGX-110S (X = F)]

9-halo-2-[[(2S)-2,3-dihydro-[1,4]dioxino[2,3-*b*]pyridin-2-yl]methoxy]-6,7-dihydropyrimido[6,1-a]isoquinolin-4-one [Compound 12 (X = Br), MGX-111S (X = F)]

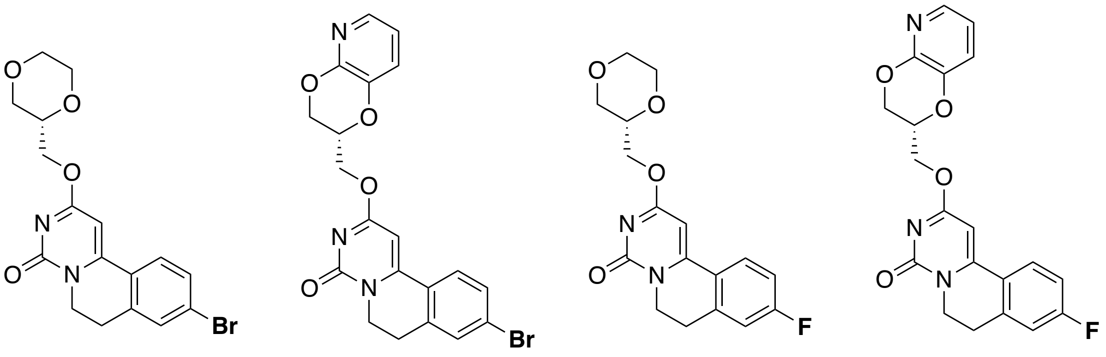

11 12 MGX-110S MGX-111S

General Procedure

9-bromo-2-chloro-6,7-dihydropyrimido[6,1-*a*]isoquinolin-4-one (compound 9, 1 equivalent) or 9-fluoro-2-chloro-6,7-dihydropyrimido[6,1-*a*]isoquinolin-4-one (compound 10, 1 equivalent) were placed in a round bottom flask and dissolved in dry THF (10 mL). Next, (2*R*)-[1,4]dioxan-2-yl-methanol or (2*R*)-hydroxymethyl-2,3-duhydro-[1,4]dioxino[2,3-*b*]pyridine (1.5 equivalent) was added and stirred for 5 minutes. Potassium *tert*-butoxide (1.5 equivalent) dissolved in dry THF (100 mg/mL concentration) was then added dropwise, changing the color of the reaction mixture to deep red. The reaction was quenched after 60 minutes by adding saturated NH_4Cl (50 mL). Ethyl acetate (100 mL) was then added and stirred for 5 minutes. Subsequently, the mixture was transferred to a separation flask and the organic layer was washed with water (1* 100 mL), brine (1* 100 mL), dried over anhydrous Na_2SO_4, and concentrated using a rotary evaporator. The crude product was then purified

via column chromatography using silica gel (20-45 μm) stationary phase and ethyl acetate/methanol gradient (from 1% MeOH to 5% MeOH) mobile phase.

Compound 11

Compound color = yellow solid, Percentage yield: 64.41%

^{1}H NMR (400 MHz, Chloroform-*d*): δ 7.44 – 7.19 (m, 3H), 6.13 (s, 1H), 4.33 – 4.08 (m, 2H), 3.97 (t, *J* = 6.4 Hz, 2H), 3.75 (dq, *J* = 6.7, 3.2 Hz, 1H), 3.69 – 3.36 (m, 5H), 3.25 (t, *J* = 10.8 Hz, 1H), 2.78 (t, *J* = 6.4 Hz, 2H)

^{13}C NMR (400 MHz, Chloroform-*d*): δ 170.66, 156.72, 151.40, 138.02, 131.34, 131.08, 127.22, 126.61, 126.17, 90.46, 73.13, 67.72, 66.60, 66.34, 66.12, 40.12, 27.58

HRMS (ESI): m/z calculated for $C_{17}H_{17}BrN_2O_4$ $[M+H]^+$ 393.0478, found 393.0460

MGX-110S

Compound color = pale yellow solid, Percentage yield: 72.96%

^{1}H NMR (400 MHz, Chloroform-*d*): δ 7.70 (dd, *J* = 8.7, 5.3 Hz, 1H), 7.15 – 6.95 (m, 2H), 6.31 (s, 1H), 4.51 – 4.33 (m, 2H), 4.26 – 4.15 (m, 2H), 3.97 (dddd, *J* = 10.1, 6.3, 3.8, 2.7 Hz, 1H), 3.90 – 3.59 (m, 5H), 3.47 (dd, *J* = 11.5, 10.1 Hz, 1H), 3.01 (t, *J* = 6.5 Hz, 2H)

^{13}C NMR (400 MHz, Chloroform-*d*): δ 170.70, 165.91, 163.37, 156.80, 151.50, 139.15, 139.06, 128.34, 128.24, 123.53, 123.50, 115.44, 115.34, 115.22, 115.12, 90.14, 73.19, 67.76, 66.64, 66.37, 66.10, 40.12, 27.92, 27.90

^{19}F NMR (400 MHz, Chloroform-*d*): δ -106.45, -106.46, -106.47, -106.48, -106.49, -106.51 (td, *J* = 8.3, 5.3 Hz, 1F)

HRMS (ESI): m/z calculated for $C_{17}H_{17}FN_2O_4$ $[M+H]^+$ 333.1278, found 333.1209

Compound 12

Compound color = off white/grey solid, Percentage yield: 74.42%

^{1}H NMR (400 MHz, Chloroform-*d*): δ 7.83 (dd, *J* = 4.8, 1.7 Hz, 1H), 7.65 – 7.40 (m, 3H), 7.23 (dd, *J* = 7.9, 1.6 Hz, 1H), 6.88 (dd, *J* = 7.9, 4.8 Hz, 1H), 6.33 (d, *J* = 2.7 Hz, 1H), 4.68 (d, *J* = 5.0 Hz, 2H), 4.61 – 4.46 (m, 2H), 4.32 (dd, *J* = 11.7, 7.2 Hz, 1H), 4.25 – 4.10 (m, 2H), 3.00 (t, *J* = 6.4 Hz, 2H)

^{13}C NMR (400 MHz, Chloroform-*d*): δ 170.77, 156.95, 152.23, 150.86, 140.49, 138.82, 138.41, 131.78, 131.51, 127.65, 127.21, 126.42, 125.41, 119.09, 90.52, 71.06, 65.88, 64.88, 40.57, 27.94

HRMS (ESI): m/z calculated for $C_{20}H_{16}BrN_3O_4$ $[M+H]^+$ 442.0378, found 442.0405

MGX-111S

Compound color = off white solid, Percentage yield: 47.44%

^{1}H NMR (400 MHz, Chloroform-*d*): δ 7.91 – 7.65 (m, 2H), 7.23 (dd, *J* = 7.8, 1.5 Hz, 1H), 7.14 – 6.97 (m, 2H), 6.94 – 6.82 (m, 1H), 6.30 (d, *J* = 4.7 Hz, 1H), 4.68 (d, *J* = 5.0 Hz, 2H), 4.62 – 4.47 (m, 2H), 4.39 – 4.15 (m, 3H), 3.02 (t, *J* = 6.5 Hz, 2H).
^{13}C NMR (400 MHz, Chloroform-*d*): δ 170.56, 166.16, 163.62, 156.79, 152.10, 150.66, 140.27, 139.36, 139.27, 138.62, 128.59, 128.50, 125.20, 123.54, 118.87, 115.64, 115.55, 115.41, 115.33, 89.98, 70.88, 65.68, 64.60, 40.34, 28.03.
^{19}F NMR (400 MHz, Chloroform-*d*): δ -106.08, -106.10, -106.11, -106.12, -106.13, -106.14. (dt, *J* = 8.5, 4.3 Hz, 1F).
HRMS (ESI): m/z calculated for $C_{20}H_{16}FN_3O_4$ $[M+H]^+$ 382.1178, found 382.1209

9-*O*-Pinacolatoboro-2-[[(2S)-1,4-dioxan-2-yl]methoxy]-6,7-dihydropyrimido[6,1-a]isoquinolin-4-one (MGX-90S)
9-*O*-Pinacolatoboro-2-[[(2S)-2,3-dihydro-[1,4]dioxino[2,3-*b*]pyridin-2-yl]methoxy]-6,7-dihydropyrimido[6,1-a]isoquinolin-4-one (MGX-100S)

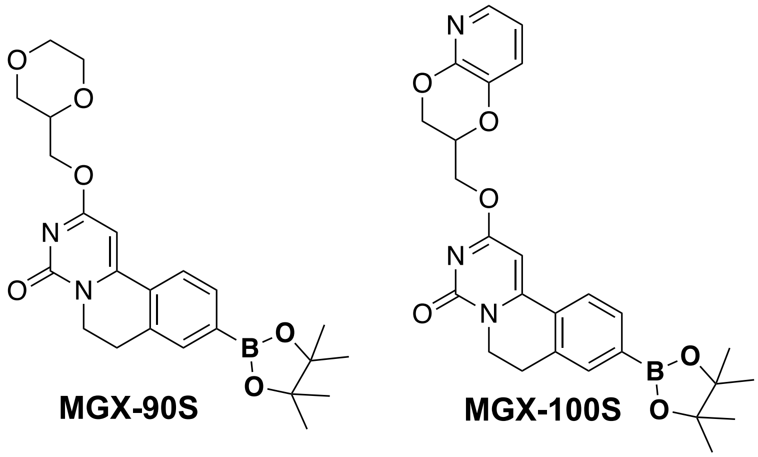

General Procedure
Compound 11 or 12 (1 equivalent) in a sealed tube equipped with a stir bar was dispersed in anhydrous toluene (10 mL) followed by degassing with nitrogen purge for 10 min. Next, bis(pinacolato)diboron (1.5 equivalent) and potassium acetate (3 equivalent) were added to the same tube. This mixture was purged with nitrogen again for another 10 min followed by addition of [Pd)dppf)$_2$Cl$_2$] (0.2 equivalent). The tube was sealed and stirred at 100 °C for 2 hours. The reaction was allowed to reach room temperature, filtered through a celite pad, and washed with diethyl ether (3 *50 mL). The solvents were removed using a rotary evaporator and the crude product was purified *via* preparatory TLC on silica gel plate using ethyl acetate/methanol (3% MeOH) mobile phase. (NOTE: purifying these compounds through column chromatography worked sub-optimally presumably due to labile boronic ester on silica gel)

MGX-90S

Compound color = pale yellow or dark grey solid, Percentage yield: 36.86%

^{1}H NMR (400 MHz, Chloroform-*d*): δ 7.82 – 7.75 (m, 1H), 7.73 (s, 1H), 7.68 (d, *J* = 7.9 Hz, 1H), 6.41 (d, *J* = 1.3 Hz, 1H), 4.50 – 4.33 (m, 2H), 4.23 – 4.14 (m, 2H), 3.97 (dddd, *J* = 9.0, 6.3, 3.8, 1.9 Hz, 1H), 3.89 – 3.60 (m, 5H), 3.48 (dd, *J* = 11.5, 10.1 Hz, 1H), 3.01 (t, *J* = 6.5 Hz, 2H), 1.35 (s, 12H)

^{13}C NMR (400 MHz, Chloroform-*d*): δ 170.73, 156.97, 152.21, 135.14, 134.49, 133.83, 129.43, 124.84, 90.84, 84.33, 74.99, 73.20, 67.79, 66.63, 66.37, 66.06, 40.49, 27.66, 24.84

HRMS (ESI): m/z calculated for $C_{23}H_{29}BN_2O_6$ $[M+H]^+$ 441.2178, found 441.2209

MGX-100S

Compound color = pale yellow solid, Percentage yield: 19.66%

^{1}H NMR (400 MHz, Chloroform-*d*): δ 7.88 – 7.77 (m, 2H), 7.77 – 7.67 (m, 2H), 7.23 (dd, *J* = 7.8, 1.6 Hz, 1H), 6.89 (dd, *J* = 7.9, 4.8 Hz, 1H), 6.41 (s, 1H), 4.69 (d, *J* = 5.0 Hz, 2H), 4.63 – 4.50 (m, 2H), 4.33 (dd, *J* = 11.6, 7.2 Hz, 1H), 4.27 – 4.11 (m, 2H), 3.03 (t, *J* = 6.5 Hz, 2H), 1.36 (s, 12H)

^{13}C NMR (400 MHz, Chloroform-*d*): δ 170.82, 157.18, 153.04, 150.90, 140.48, 138.86, 135.55, 134.91, 134.22, 129.66, 125.43, 125.28, 119.09, 90.89, 84.74, 71.12, 65.93, 64.82, 40.93, 28.01, 25.21

HRMS (ESI): m/z calculated for $C_{26}H_{28}BN_3O_6$ $[M+H]^+$ 490.2178, found 490.2163

Scheme S1. Synthesis of Bpin precursor for ^{18}F labeling using alternative route (10 steps synthesis)

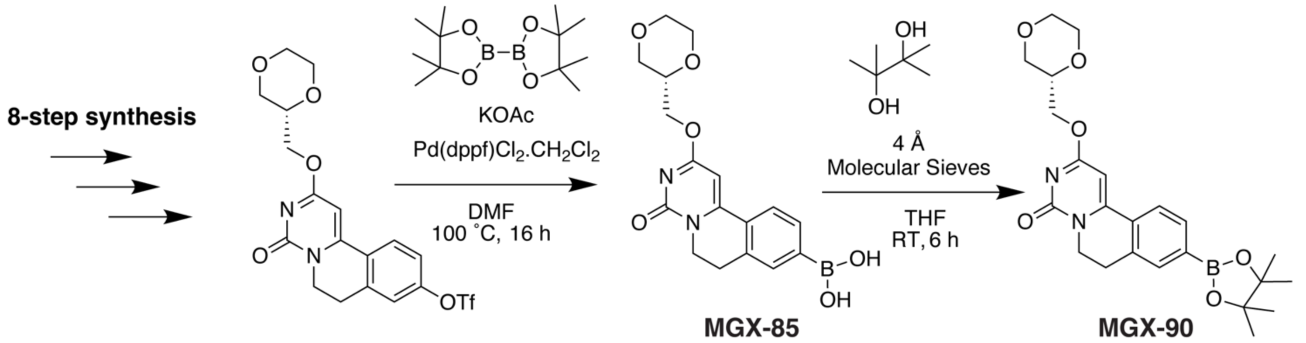

(*S*)-(2-((1,4-dioxan-2-yl)methoxy)-4-oxo-6,7-dihydro-4H-pyrimido[6,1-a]isoquinolin-9-yl)boronic acid (MGX-85S)

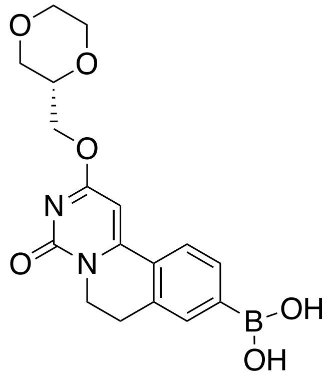

MGX-85S

Under a nitrogen atmosphere, (S)-2-((1,4-dioxan-2-yl)methoxy)-4-oxo-6,7-dihydro-4H-pyrimido[6,1-a]isoquinolin-9-yl trifluoromethanesulfonate (0.5 g, 1.08 mmol) was placed in a sealed tube containing dimethylformamide (10 mL) and purged with nitrogen for 10 minutes. Following this, Bis(pinacolato)diboron (0.41 g, 1.62 mmol) and potassium acetate (0.31 g, 3.24 mmol) were introduced into the reaction mixture. Nitrogen purging was continued for an additional 5 minutes before the addition of [1,1'-Bis(diphenylphosphino)ferrocene]palladium(II) dichloride (0.17 g, 0.21 mmol). The sealed tube was then heated to 100 °C overnight. Upon cooling to room temperature, water was added to the reaction mixture, which was stirred for 10 minutes. The resulting brown solid was collected by filtration and washed thoroughly with water, followed by hexane. The solid was subsequently taken up in acetone (25 mL) and stirred for 15 minutes, then filtered to afford the desired product as a pale-brown solid (0.16 g, 41%).

^{1}H NMR (400 MHz, DMSO-*d*$_6$): δ 7.89 (d, *J* = 8.0 Hz, 1H), 7.81 – 7.57 (m, 2H), 6.61 (s, 1H), 4.23 (d, *J* = 4.9 Hz, 2H), 4.04 – 3.94 (m, 2H), 3.92 (s, 2H), 3.87 – 3.78 (m, 1H), 3.73 (td, *J* = 12.1, 2.8 Hz, 2H), 3.66 – 3.50 (m, 2H), 3.50 – 3.40 (m, 1H), 3.35 (dd, *J* = 11.5, 10.0 Hz, 1H), 2.96 (t, *J* = 6.5 Hz, 2H)

^{13}C NMR (400 MHz, DMSO-*d₆*): 170.73, 156.06, 135.50, 134.23, 133.35, 128.57, 125.65, 90.01, 73.18, 67.56, 66.16, 65.78, 27.44
HRMS (ESI): m/z calculated for $C_{17}H_{19}BNO_2O_6$ $[M+H]^+$ 359.1409, found 359.1399

(*S*)-2-((1,4-dioxan-2-yl)methoxy)-9-(4,4,5,5-tetramethyl-1,3,2-dioxaborolan-2-yl)-6,7-dihydro-4H-pyrimido[6,1-a]isoquinolin-4-one (MGX-90S)

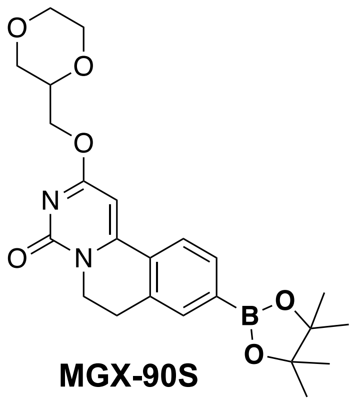

MGX-90S

Under a nitrogen atmosphere, (*S*)-(2-((1,4-dioxan-2-yl)methoxy)-4-oxo-6,7-dihydro-4H-pyrimido[6,1-a]isoquinolin-9-yl)boronic acid (165 mg, 0.460 mmol) and approximately 2 g of 4 Å molecular sieves were placed in an oven-dried round-bottom flask (RBF) containing 8 mL of THF. Pinacol (0.16 g, 1.38 mmol) was then added at room temperature, and the mixture was stirred for 6 hours. The molecular sieves were removed by filtration, and the filtrate was evaporated to dryness. Diethyl ether (20 mL) was added, and the undissolved solid was removed by filtration. The volume of the filtrate was reduced until precipitation was observed, and the resulting solid was collected by filtration. The solid was washed with copious amounts of hexane and dried under vacuum to yield the desired product as a pale yellow solid (38 mg, 19%).

^{1}H NMR (400 MHz, $CDCl_3$): δ 7.82 – 7.77 (m, 1H), 7.74 (s, 1H), 7.69 (d, *J* = 7.8 Hz, 1H), 6.42 (s, 1H), 4.41 (qd, *J* = 11.9, 5.1 Hz, 2H), 4.20 (t, *J* = 6.5 Hz, 2H), 4.03 – 3.92 (m, 1H), 3.91 – 3.61 (m, 5H), 3.48 (dd, *J* = 11.5, 10.1 Hz, 1H), 3.01 (t, *J* = 6.5 Hz, 2H), 1.36 (s, 12H)
^{13}C NMR (400 MHz, Chloroform-*d*): δ 170.73, 156.97, 152.21, 135.14, 134.49, 133.83, 129.43, 124.84, 90.84, 84.33, 74.99, 73.20, 67.79, 66.63, 66.37, 66.06, 40.49, 27.66, 24.84
HRMS (ESI): m/z calculated for $C_{23}H_{29}BN_2O_6$ $[M+H]^+$ 441.2178, found 441.2209

Scheme S2. Optimization process to access 9-bromo-2-chloro-6,7-dihydropyrimido[6,1-*a*]isoquinolin-4-one (**compound 7**), an intermediate for the synthesis of **MGX-90** and **MGX-100** precursors

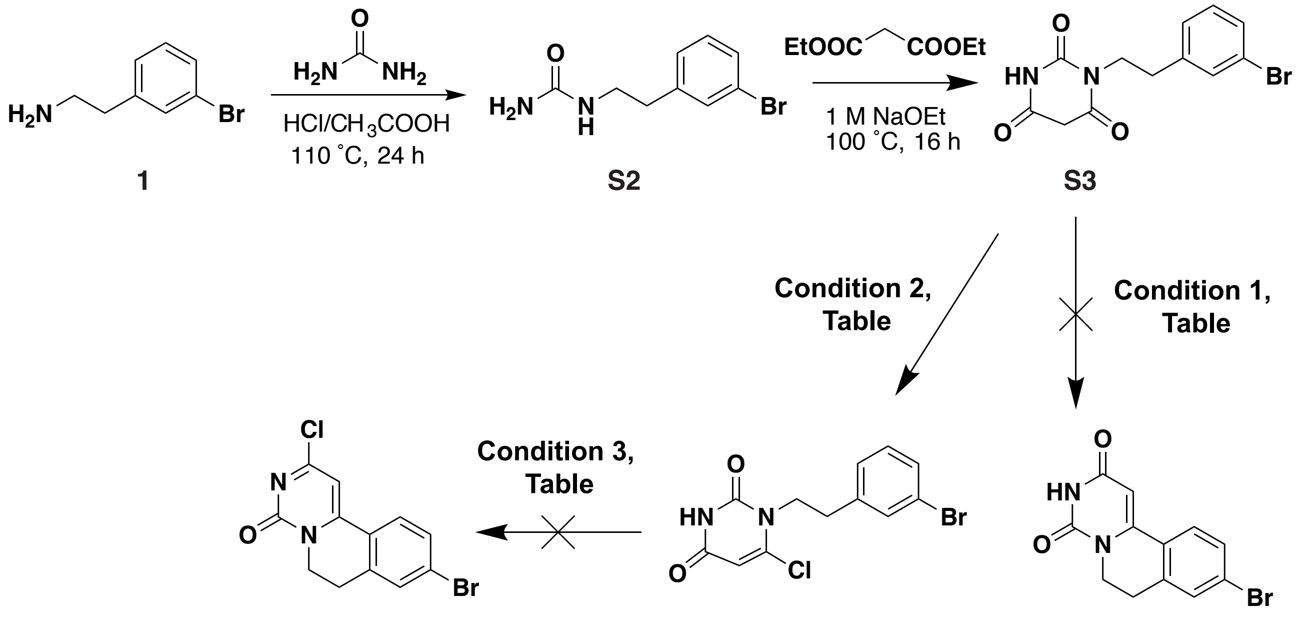

1-(3-bromophenethyl)pyrimidine-2,4,6(1*H*,3*H*,5*H*)-trione (S3)

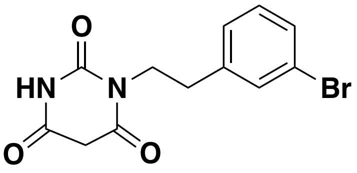

S3

Diethyl malonate (1.25 mL, 8.23 mmol, 2 equivalent) was added into a 48 mL heavy wall pressure vessel equipped with a stir bar under constant flow of nitrogen gas. Added freshly prepared 1M sodium ethoxide (8.23 mL, 8.23 mmol, 2 equivalent) dropwise while stirring. The vessel was sealed, and reaction was heated to 100 °C for 30 minutes by immerging the vessel into oil bath. Oil bath was removed, reaction was allowed to reach room temperature, unsealed the vessel, and added compound **3** (1 g, 4.11 mmol, 1 equivalent) dissolved in anhydrous 200 proof ethanol (10 mL). The vessel was sealed and heated to 100 °C by immerging it in oil bath and allowed to heat for 16 h. Next, the reaction was removed from the oil bath, allowed to reach room temperature, acidified by adding 1 N HCl (pH ~5). This produced grey precipitate. The vessel was kept at 4 °C for 12 hours, which resulted more solid product. The precipitate was filtered through a frit funnel and washed with cold water (500 mL). This reaction gave us 1.18 g compound **S3**.

Compound color = grey solid, Percentage yield: 92.91%

^{1}H NMR (400 MHz, CDCl$_3$): δ 7.93 (s, 1H), 7.37 (d, *J* = 8.7 Hz, 2H), 7.21 – 7.08 (m, 2H), 4.11 – 3.94 (m, 2H), 3.61 (s, 2H), 2.96 – 2.75 (m, 2H)
HRMS (ESI): m/z calculated for $C_{12}H_1BrN_2O_3$ $[M+H]^+$ 311.0026, found 311.0023

1-(3-bromophenethyl)-6-chloropyrimidine-2,4(1*H*,3*H*)-dione (S4)

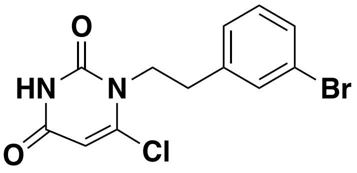

S4

Compound **S3** (1 g, 3.214 mmol) was placed into a 100 mL round bottom flask (RBF) equipped with a stir bar. The compound was subjected to three cycles of vacuum and nitrogen. Added 20 mL $POCl_3$ and stirred at 65 °C for 2 days. The reaction was cooled to room temperature and directly poured into ice cold water (300 mL) inside a 1L conical flask, which was kept in an ice bath. The pH of this mixture was brought to 7-8 by *slowly* adding sodium carbonate in the solid form while stirring. This step produced foam/froth. Once pH is reached pH 7-8, the yellow precipitate was filtered through a frit, washed with cold water (1000 mL) while stirring the precipitate manually with a glass rod. The compound was dried under vacuum. This precipitation method yielded 4.13 g of Compound **S4** (14.30 mmol, 75.05%).

Compound color = yellow solid, Percentage yield: 92.91%

^{1}H NMR (400 MHz, CDCl$_3$): δ 7.46 – 7.39 (m, 1H), 7.34 (dt, *J* = 7.2, 1.8 Hz, 1H), 7.22 – 7.11 (m, 2H), 5.86 (d, *J* = 1.7 Hz, 1H), 4.14 – 4.02 (m, 2H), 2.87 (dd, *J* = 9.0, 6.8 Hz, 2H)
^{13}C NMR (400 MHz, CDCl$_3$): δ 161.35, 151.28, 142.64, 140.31, 131.96, 130.08, 129.78, 127.55, 122.51, 101.29, 41.77, 33.18.
HRMS (ESI): m/z calculated for $C_{12}H_{10}BrClN_2O_2$ $[M+H]^+$ 328.9687, found 328.9686

C. General Procedure for Manual Radiochemistry

Scheme S3. Manual Radiosynthesis of [^{18}F]MGX-110S and [^{18}F]MGX-111S

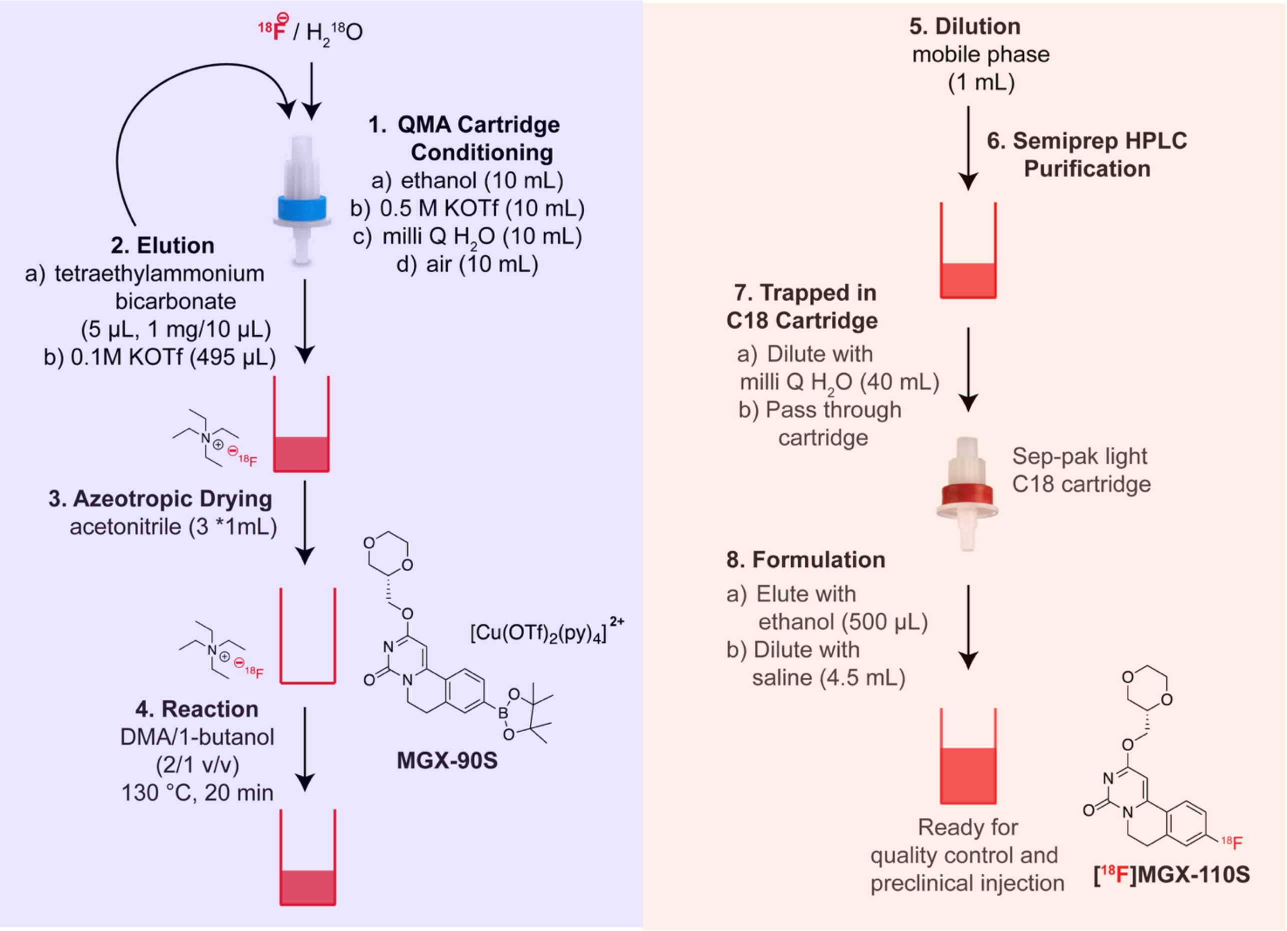

No carrier added-aqueous [^{18}F]-fluoride ion was produced on a PETtrace cyclotron (GE Healthcare) by irradiation of a 2.5 mL water target using a 16 MeV proton beam on 95% enriched [^{18}O]H_2O by the [^{18}O(p,n)^{18}F] nuclear reaction. [^{18}F]fluoride was trapped on a QMA-light Sep-Pak cartridge (Waters) that was preconditioned with ethanol (10 mL), 0.5 M potassium trifluoromethanesulfonate (KOTf) aqueous solution (10 mL), and Milli-Q H_2O (10 mL). The cartridge was dried by pushing air (10 mL) through a syringe. [^{18}F]fluoride was trapped in the cartridge and then eluted into the reaction vial using a solution of tetraethylammonium bicarbonate (0.5 mg or 5 µL of 1 mg/10 µL stock concentration) and KOTf (9.3 mg or 495 µL of 0.1 M KOTf) in 0.5 mL of water. The [^{18}F]fluoride was then treated with acetonitrile (3* 1 mL) and azeotropically dried at 110 °C under vacuum and helium pressure. When dry [^{18}F]fluoride (a white layer formed around bottom of the reaction vial) reached approximately 50 °C, reaction solvent mixture (DMA/1-butanol, v/v 2/1, 250 µL) was added to the vial, vortexed for 2-3 seconds, and dissolved [^{18}F]fluoride. Next, a solution of MGX-90S or MGX-100S precursor (2.5 mg, 1 equivalent) and $Cu(OTf)_2py_4$ (4.62 mg, 1.2 equivalent) in a solvent mixture of (DMA/1-butanol, v/v 2/1, 250 µL) was added, and the reaction was heated at 130 °C for 20 min.

Manual Synthesis Analysis

Radio-TLC of crude: After cooling to 50 °C, a small aliquot of reaction mixture (10 μL) was added to a vial of acetone (1 mL) for radio-TLC analysis. Crude reaction mixture was spotted on a standard silica gel TLC plate and developed in ethyl acetate/methanol (10/1.2 v/v) solvent mixture. The product retention factor (Rf) was ~0.25 (Figure S2). The radiochemical conversion (% RCC) was calculated using following formula:

$$\%RCC=\frac{integration\ of\ the\ product\ peak}{sum\ of\ integration\ of\ all\ peaks} * 100$$

HPLC analysis of crude: An aliquot of the crude reaction mixture (20 μL in 100 μL HPLC mobile phase) was injected onto a reversed-phase analytical HPLC column (Phenomenex Gemini 5 μm C18 110 Å, LC column 250 x 4.6 mm, P/No. 00G-4435-E0, S/No. H21-308418) using mobile phase (water + 0.1% TFA/MeCN + 0.1% TFA (isocratic 60:40) with a flow rate of 1 mL/min to elute [^{18}F]MGX-110S with a retention time (Rt) of 7.47 min. To confirm identity, crude reaction mixture was spiked with 1 mg/mL reference standard in HPLC mobile phase (100 μL reference standard solution was added to the above crude reaction mixture after first HPLC injection and briefly agitated).

Manual semi-prep HPLC purification and formulation: After crude reaction mixture was cooled down to approximately 50 °C, it was diluted with mobile phase (1.0 mL) and purified by semi-preparative HPLC with mobile phase: water + 0.1% TFA/MeCN + 0.1% TFA (isocratic 65:35) as an eluent at a 4 mL/min flow rate (semiprep column: Gemini 5 μm C18 110 Å, LC column 250 x 10 mm, P/No. 00G-4435-N0, S/No. H19-371945). The collected peak (8.5 to 9.5 min) in a 50 mL falcon tube was diluted with Milli-Q water (40 mL), trapped in a pre-conditioned sep-pak light C18 cartridge [pre-conditioned with ethanol (5 mL) and Milli-Q water (20 mL)], and pure tracer was eluted with ethanol (500 μL) into a product vial. Finally, the product was diluted with saline (4.5 mL) to achieve final formulation of 10% ethanol in saline. Quality control tests (HPLC of pure tracer and co-injections) were performed on the end of synthesis (EOS) formulation. For [^{18}F]MGX-110S, decay uncorrected radiochemical yield = 10.02 ± 0.03% (n = 5), molar activity = 3.58 ± 0.43 Curie/μmol or 132.59 ± 16.11 GBq/μmol (n = 5; **Table S7**).

Analytical HPLC analysis of EOS tracer

To determine molar activity, radiochemical and chemical purity, an aliquot of undiluted final product (120 μL) of known radioactivity was injected onto an analytical reversed-phase HPLC column (Phenomenex Gemini 5 μm C18 110 Å, LC column 250 x 4.6 mm, P/No. 00G-4435-E0, S/No. H21-308418) using mobile phase (water + 0.1% TFA/MeCN + 0.1% TFA (isocratic 60:40) with a flow rate of 1 mL/min to elute [^{18}F]MGX-110S and [^{18}F]MGX-111S with an elution time of 7.51 min and 8.34 min respectively. The area of the UV absorbance peak measured at 254 nm corresponding to the carrier product was measured (integrated) on the HPLC chromatogram and compared to a standard

curve relating mass to UV absorbance. Molar activity (Am) was calculated using the equation Am = A/n, where A is the activity of pure tracer (expressed in becquerels or Curies) and n is the moles of reference standards (calculated from the area under UV absorbance peak of the carrier product at 254 nm and then compared to the standard curve of the reference standard (MGX-110S or MGX-111S).

The tracer identity was confirmed by co-injecting the reference standard with final tracer (added 100 μL of 1 mg/mL stock solution to the HPLC vial containing pure tracer).

D. Automation of [^{18}F]MGX-110S and [^{18}F]MGX-111S Radiotracers

[^{18}F]Fluoride in [^{18}O]H_2O was transferred to a GE TRACERlab FXFN synthesizer and trapped on an anion exchange resin [QMA-light cartridge, prepared by preconditioning with 10 mL of EtOH, 10 mL 0.5 M potassium trifluoromethanesulfonate (KOTf), 10 mL Milli-Q H_2O, and dried with 10 mL air], followed by elution with 500 μL of an aqueous solution containing 0.1 M KOTf (495 μL, 5 mg) and tetraethylammonium bicarbonate (1 mg/10 μL, 0.5 mg), followed by azeotropic drying with 1 mL acetonitrile at 110 °C. The reactor was cooled down to 70 °C with compressed air. Next, a solution of MGX-90S (or MGX-100S) precursor (2 mg, 1 equivalent), $Cu(OTf)_2$ (8.2 mg, 5 equivalent), and pyridine (44 μL, 125 equivalent) in solvent mixture (DMA/1-butanol, v/v 2/1, 250 μL) were added to the reactor in this order and stirred at 130 °C for 18 minutes. The reaction mixture was then cooled to 50 °C with compressed air and 2 mL of HPLC mobile phase [water + 0.1% TFA/MeCN + 0.1% TFA (3:2)] was added to the reactor. The crude reaction mixture was then injected onto a semi-preparative column (Gemini 5 μm C18 110 Å, LC column 250 x 10 mm, P/No. 00G-4435-N0, S/No. H19-371945) using water + 0.1% TFA/MeCN + 0.1% TFA (isocratic 70:30) as an eluent at a 4 mL/min flow rate. The radioactive peak at retention time (Rt) 17 to 19 min ([^{18}F]MGX-110S) or 22 to 24 min ([^{18}F]MGX-111S) was collected into a dilution flask containing 20 mL water (Figure S4). The resulting solution was passed through a pre-conditioned sep-pak light C18 cartridge (conditioned with 5 mL ethanol, 20 mL Milli-Q water) and rinsed with 10 mL water. The pure tracer trapped on the cartridge was eluted with 0.5 mL anhydrous ethanol and rinsed with 9.5 mL of sterile saline into the product vial to give formulated [^{18}F]MGX-110S (or [^{18}F]MGX-111S). The product vial was transferred from the synthesis module into a lead pig and total activity was measured using a dose calibrator. Identity of the product and molar activity were determined with HPLC (see "**HPLC analysis of EOS tracer**" above for further details).

E. Supplementary Methods

Study design

This study was designed to develop ^{18}F PET tracers for GPR84 and assess their sensitivity and specificity for detecting innate immune activation using the LPS-induced systemic and neuroinflammation model and the 5xFAD transgenic mouse model of AD. To characterize tracer pharmacology, we performed *in vitro* cell binding, saturation binding, and competitive binding assays. *In vivo* PET imaging, *ex vivo* biodistribution, and autoradiography were then used to assess the sensitivity and specificity of GPR84-PET tracers in detecting innate immune activation in both models. for comparative benchmarking, TSPO-PET was performed using [^{18}F]GE180, followed by *ex vivo* gamma counting and autoradiography. In addition, RNA was extracted from brain tissue for qPCR analyses to measure *Gpr84* and *Tspo* expression and validate tracer specificity. Sample sizes were determined empirically, consistent with standards in the field, to provide sufficient power to detect statistical differences. Experiments were not blinded.

<u>Flow cytometry: antibody details</u>

Flow cytometry was performed using the following antibodies: AF488 CD45 (BioLegend, San Diego, CA, #160305), PB CD11b (BioLegend, #101223), BV570 CD68 (BioLegend, #137021), and PE GPR84 (Alomone labs, Israel, #AGR-052-PE).

<u>PET imaging analysis</u>

PET analysis was not conducted truly blind as researchers had access to mouse group assignments. To minimize bias, each mouse was assigned a unique identifier-based scanning group and bed position (e.g., G1P1, G1P2), and analyses were performed using these coded labels. Group identities were only revealed after analysis for data aggregation and visualization. In addition, multiple researchers independently analyzed the data to enhance rigor and reproducibility.

F. Supplementary Figures

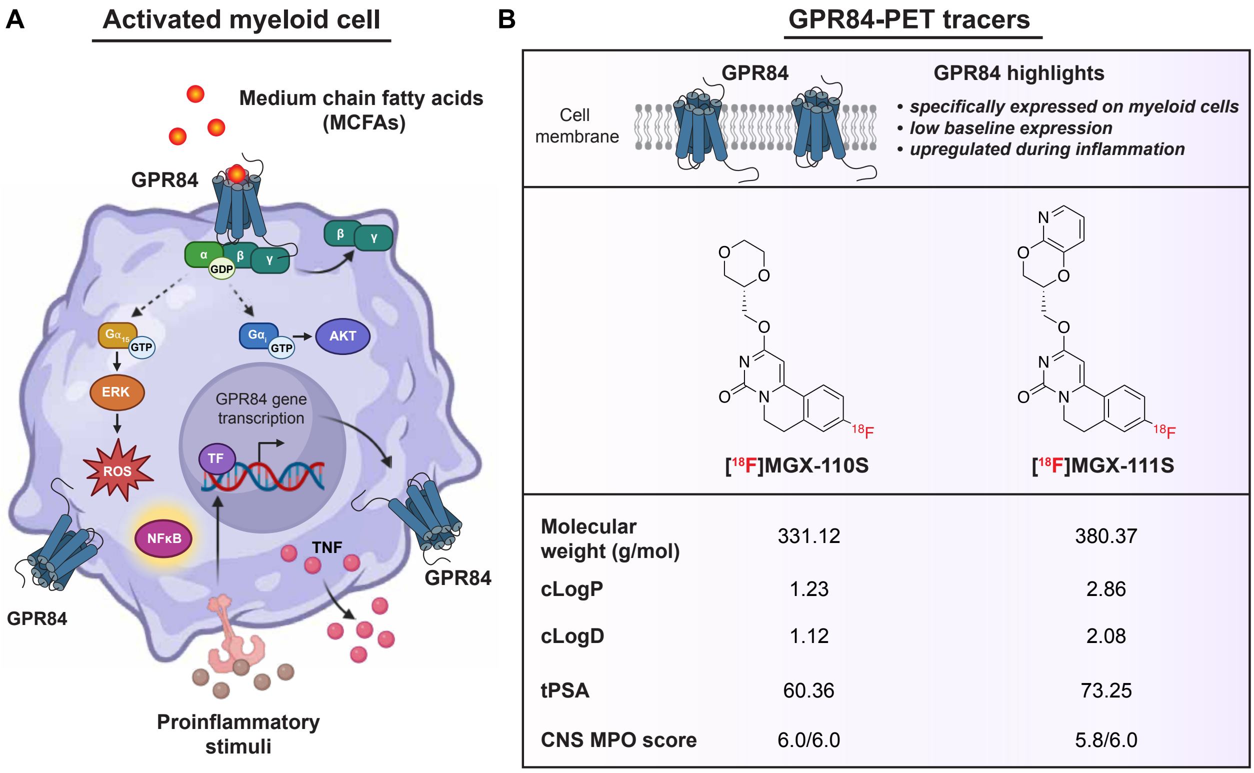

Figure S1. **GPR84 is a driver of myeloid activation and a functionally relevant PET biomarker.** (A) GPR84 is markedly upregulated in activated myeloid cells, particularly monocytes, macrophages, and microglia, under inflammatory conditions. Activated by medium-chain fatty acids, it amplifies pro-inflammatory signaling through Gi-mediated pathways, driving calcium mobilization, NF-κB activation, and secretion of cytokines and reactive oxygen species. These features establish GPR84 as a functional marker of maladaptive innate immune activation and a potential therapeutic and imaging target in neuroinflammatory disease. (B) Chemical scaffolds of previously reported ^{11}C-labeled tracers and newly designed ^{18}F-labeled tracers, along with calculated multiple parameter optimization (MPO) scores (molecular weight, cLogP, cLogD, tPSA, CNS MPO scores*) predicting favorable blood-brain barrier (BBB) permeability. *Values calculated using Chemdraw and ChemAxon software.

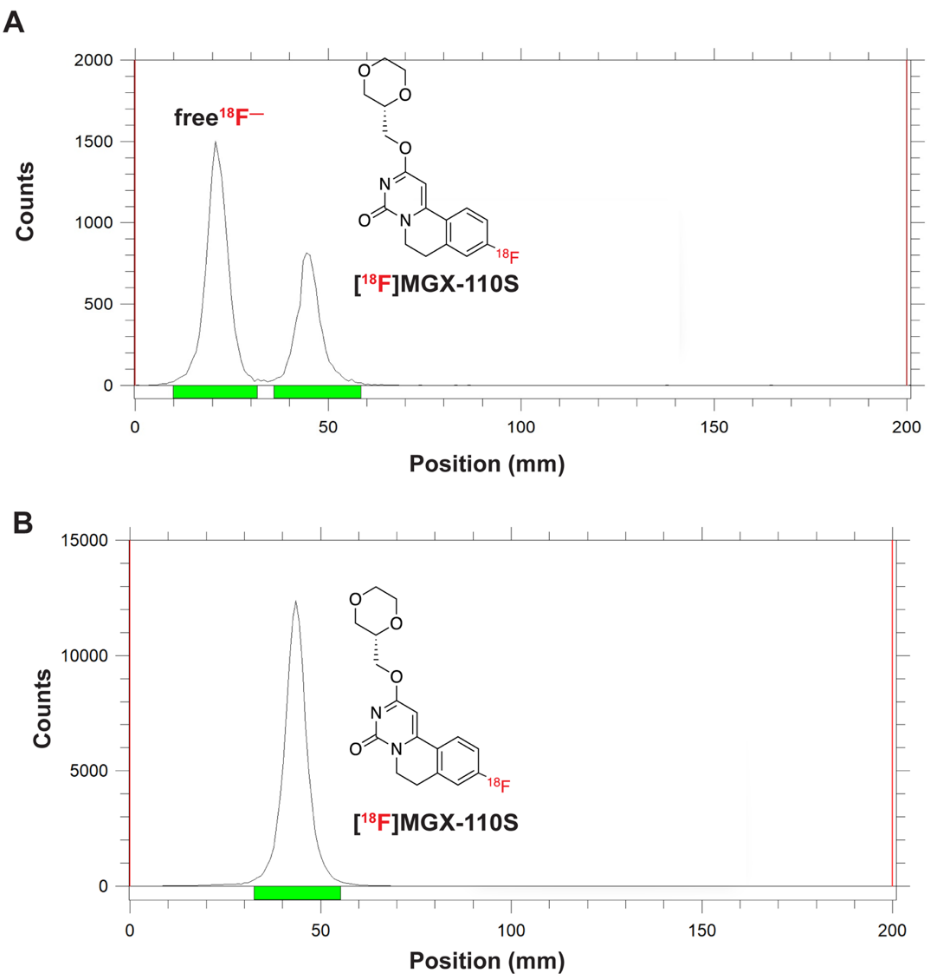

Figure S2. RadioTLC chromatography of (A) the crude reaction mixture and (B) pure [^{18}F]MGX-110S tracer. Radiochemical conversion (RCC) was measured by radioTLC (eluent = 10:1.2 ethyl acetate: methanol).

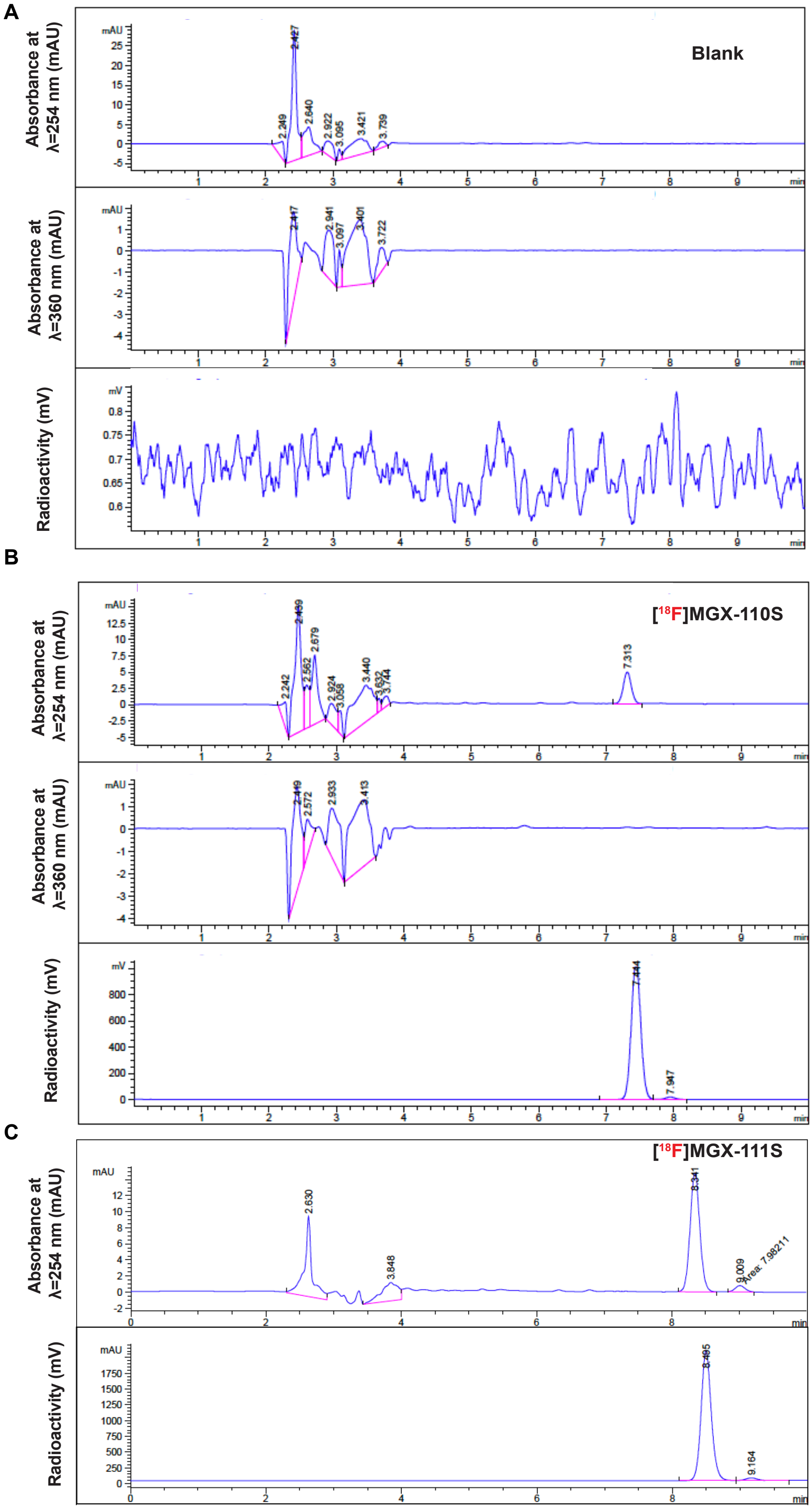

Figure S3. Percentage chemical and radiochemical purity determined using HPLC profiles of (A) saline blank, (B) [^{18}F]MGX-110S, and (C) [^{18}F]MGX-111S.

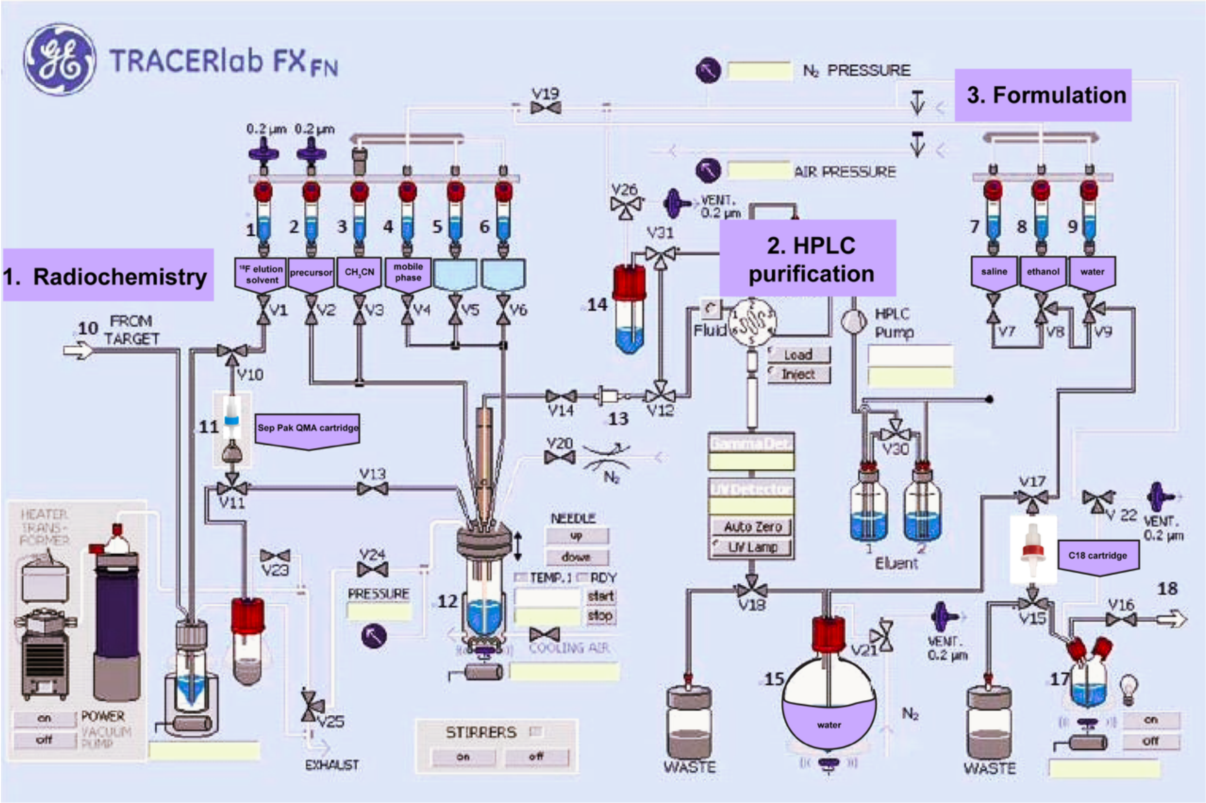

Automated Radiochemistry Highlights

- ✓ >20% radiochemical yield (decay uncorrected)
- ✓ >6 Curie/µmol (or >222 GBq/µmol) molar activity
- ✓ >99% radiochemically pure tracer
- ✓ >95% chemically pure tracer

Figure S4. Automated [^{18}F]MGX-110S and [^{18}F]MGX-111S radiosynthesis was performed using a GE TRACERlab FX_{FN} synthesizer. The automated synthesis involves three steps in sequence: 1) radiochemistry, 2) HPLC purification, and 3) formulation.

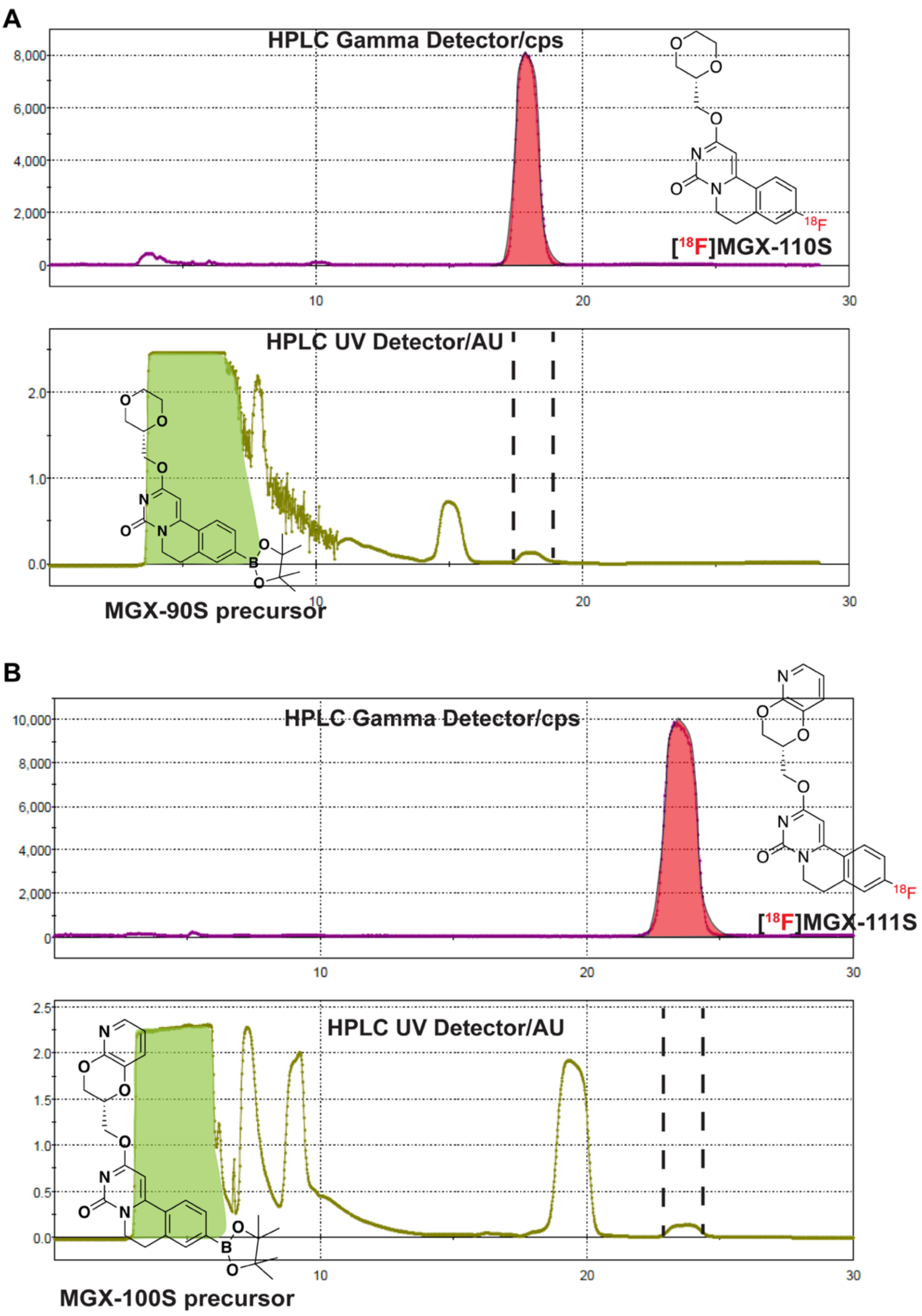

Figure S5. Semi-preparative HPLC of (A) [^{18}F]MGX-110S (red highlighted area, elution time = 17.5 min) and MGX-90S precursor (green highlighted area, elution time = 3-7 min) (B) [^{18}F]MGX-111S (red highlighted area, elution time 22.5 min) and MGX-100S precursor (green highlighted area, elution time = 3-5 min) (top = radioactivity (mV) and bottom = UV absorbance at λ = 254 nm). Mobile phase: water + 0.1% trifluoroacetic acid/acetonitrile + 0.1% trifluoroacetic acid isocratic (70:30 v/v). Semi-preparative Phenomenex column: 00G-4435-NO, Gemini 5µm C18 110 Å, 250 x 10 mm 5 micron, 597433-5.

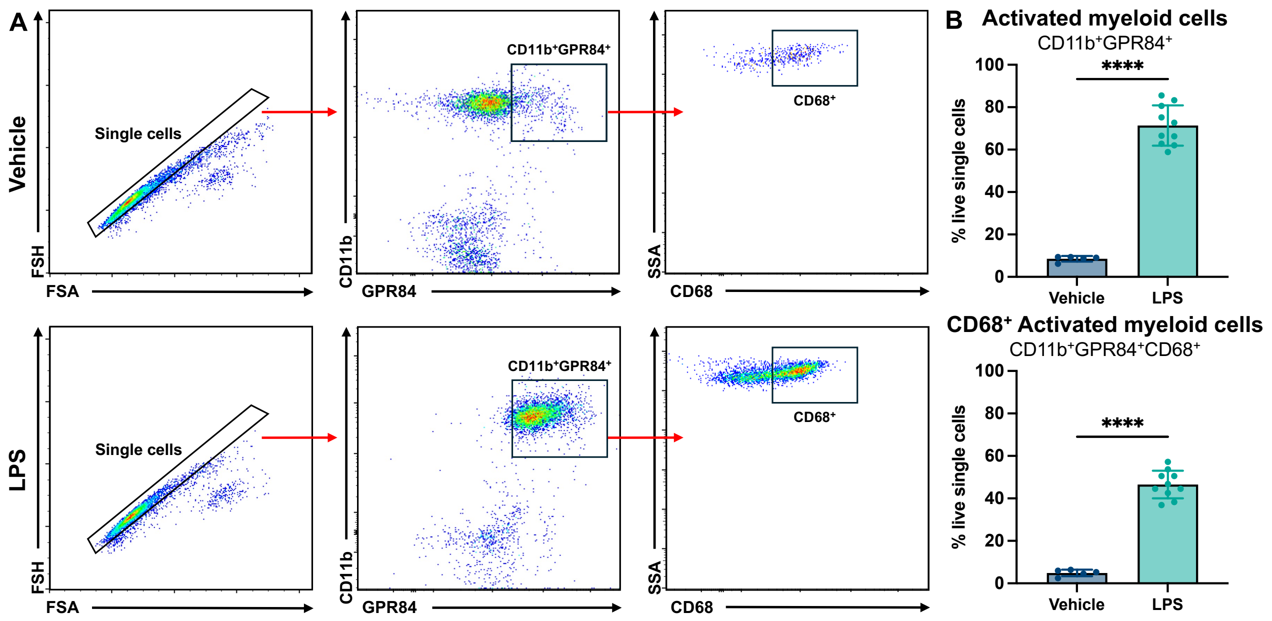

Figure S6. GPR84 is a highly specific marker for activated myeloid cells. (A) Flow cytometry gating strategy for activated myeloid cells (CD11b$^+$GPR84$^+$) and CD68$^+$ activated myeloid cells (CD11b$^+$GPR84$^+$CD68$^+$). (B) Summarized proportion of activated myeloid cells that express GPR84 in mice treated with intraperitoneal LPS (5 mg/kg) or saline (vehicle). Statistical analyses were performed using unpaired t tests. *denotes direct comparison of groups (****$P<0.0001$). n=5 vehicle and n=10 LPS. Data are shown as mean ± SD.

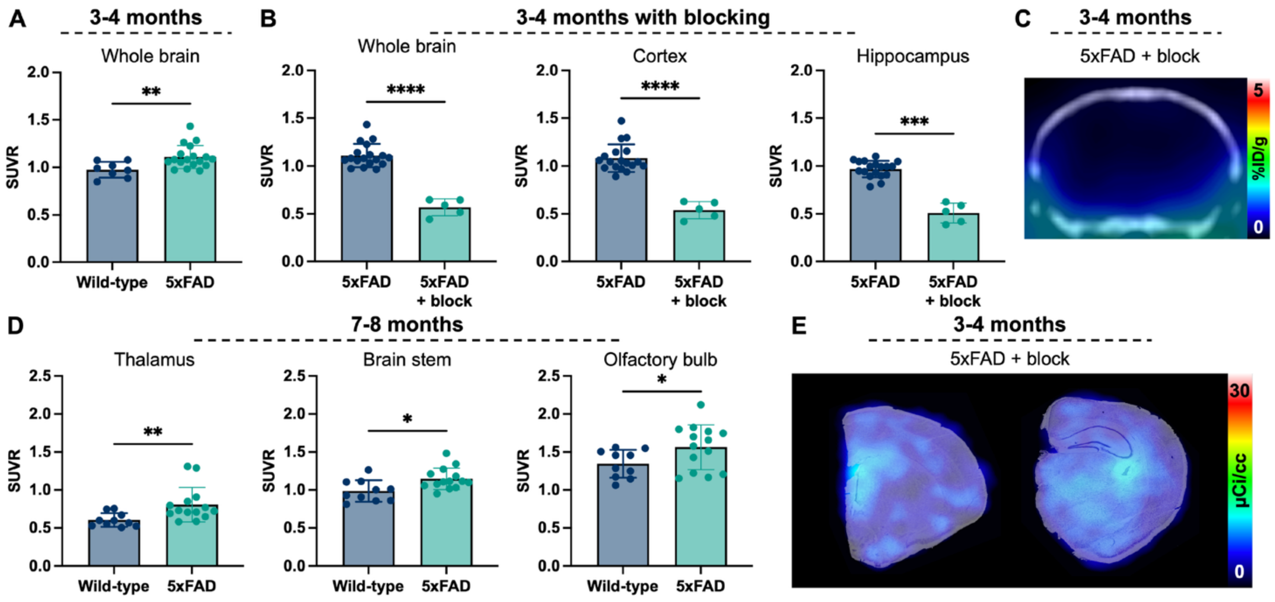

Figure S7. [18F]MGX-110S sensitively detects innate immune activation and specifically targets GPR84 in 5xFAD mice. (A) Quantification of PET images (SUVR, normalized to cerebellum) in whole brains of wild-type and 5xFAD mice at 3-4 months. (B) Blocking with GPR84 antagonist GLPG1205 (2 mg/kg) shows binding specificity in whole brain, cortex, and hippocampus. (C) Representative coronal PET/CT image showing reduced [18F]MGX-110S binding following GLPG1205 (2 mg/kg) pretreatment. (D) PET quantification of thalamus, brain stem, and olfactory bulb of 7-8-month-old 5xFAD and wild-type mice. (E) Representative *ex vivo* autoradiography images with Nissl overlay confirming tracer binding specificity to GPR84 in cortex and hippocampus of 5xFAD mice at 3-4 months. Statistical analyses were performed using unpaired t tests. *denotes direct comparison of groups (*P<0.05, **P<0.01, ***P<0.001, ****P<0.0001). n=8-10 wild-types, n=14-18 5xFAD, and n=5 5xFAD + block for *in vivo* PET quantification. Data are shown as mean ± SD.

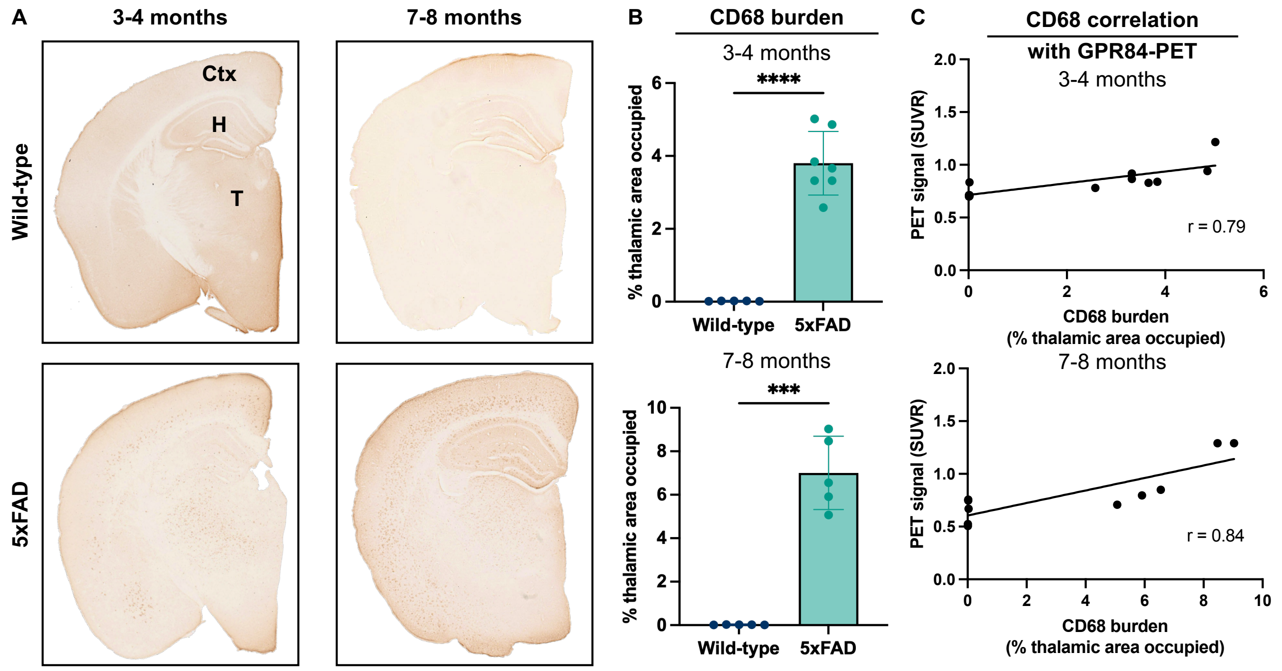

Figure S8. GPR84-PET correlates with CD68 immunohistochemistry. (A) CD68 staining of representative mouse brains. (B) Quantification of CD68 burden expressed as the percentage of thalamic area occupied by CD68$^+$ staining. (C) Correlation between CD68 burden and [^{18}F]MGX-110S PET signal (SUVR) in thalamus of 3-4 and 7-8-month-old 5xFAD and wild-type mice. Statistical analyses were performed using unpaired t tests (B) or simple linear regression (C). * denotes direct comparison of groups (***P<0.001, ****P<0.0001). n=5 wild-type and n=5-7 5xFAD mice. Ctx = cortex, H = hippocampus, T = thalamus. Data are shown as mean ± SD.

G. Supplementary Tables

Table S1. Optimization conditions for central intermediates **7** and **9** required to access final reference standards and precursors for ^{18}F labeling

Reagents	Reaction solvent	Conditions	Result
1) Compound **S4** (1 equiv)<br>Tf_2O (1.1 equiv)<br>2-chloropyridine (1.2 equiv)<br>-78 °C to room temperature	dichloromethane (DCM)	microwave (MW)<br>110 °C, 5 min<br>120 °C, 5 min<br>140 °C, 5 min	multiple undesired products
2) Compound **S4**<br>$POCl_3$	—	50 °C, 3 days<br>130 °C, 16 h	Compound **S5**
3) Compound **S5**<br>$AlCl_3$	dichloroethane (DCE)	microwave (MW)<br>90 °C, 5 min<br>100 °C, 5 min<br>100 °C, 30 min<br>no MW ,reflux, 18 h	starting material recovered

Table S2. Head-to-head comparison of radiochemistry efficacy using potassium triflate *versus* kryptofix methods

Method	^{18}F Elution solvents	Temperature (°C)	% Radiochemical conversion
Potassium triflate method	Potassium triflate (10 mg)<br>Tetraethylammonium bicarbonate (0.05 mg)<br>in 500 µL milli Q H_2O	70<br>90<br>120<br>150	0<br>0<br>20<br>40
Kryptofix method	Kryptofix 222 (15 mg)<br>K_2CO_3(3 mg)<br>in 1000 µL milli Q H_2O	70<br>90<br>120	0<br>0<br>0

Table S3. Screening solvent mixture for ^{18}F recovery from QMA cartridge

Elution Solvents	^{18}F trapped in QMA (mCi)	^{18}F eluted (mCi)	$\%^{18}F$ recovery
1) 0.1M KOTf (495 μL) tetraethylammonium bicarbonate (5 μL, 0.1 mg/μL)	3.01	2.82	93.69
2) 0.1M KOTf (495 μL) tetraethylammonium bicarbonate (5 μL, 0.1 mg/μL) + acetonitrile (0.5 mL)	2.10	1.52	72.38
3) 0.1M KOTf (495 μL) tetraethylammonium bicarbonate (5 μL, 0.1 mg/μL) + acetonitrile (1 mL)	2.08	0.55	26.20

Table S4. Optimizing manual radiochemistry conditions (molar equivalents of copper reagent, reaction temperature, and % radiochemical conversion)

Reaction solvents	$Cu(OTf)_2(py)_4$ molar equivalence	Temperature (°C)	% Radiochemical conversion
DMA/butanol 2/1 v/v	1	70<br>90<br>120<br>150	0<br>0<br>20<br>40
	0.1	120<br>150	0<br>0
DMF	1	70<br>90<br>120<br>150	0<br>0<br>0<br>0
	0.1	120<br>150	0<br>0

Table S5: Screening conditions for radiosynthesis automation

Elution Solvents	precursor (mg)	$Cu(OTf)_2(Py)_4$ or $Cu(OTf)_2$ + pyridine	^{18}F pre-dissolution	air purge	% Radiochemical conversion	% Radiochemical yield (decay uncorrected)
tetraethylammonium bicarbonate (5 µL, 0.1 mg/µL) 0.1M KOTf (495 µL) + acetonitrile (1 mL)	2.0	$Cu(OTf)_2$ + pyridine	yes	no	36.81	2.41 (calculated based on 26.20% ^{18}F eluted from QMA)
tetraethylammonium bicarbonate (5 µL, 0.1 mg/µL) 0.1M KOTf (495 µL)	3.6	$Cu(OTf)_2(Py)_4$	yes	no	5.24	1.46
	3.6	$Cu(OTf)_2(Py)_4$	yes	yes	12.49	4.40
	2.0	$Cu(OTf)_2$ + pyridine	yes	no	27.44 ± 9.08 (n=3)	22.81 ± 1.52 (n=3)
	2.0	$Cu(OTf)_2$ + pyridine	no	no	21.74 ± 2.02 (n=3)	21.89 ± 0.01 (n=3)

Table S6: Summary of manual *versus* automated radiochemistry of [^{18}F]MGX-110S (n=5 each)

Specifications	Manual radiochemistry	Automated radiochemistry
% Radiochemical yield (decay uncorrected)	10.02 ± 0.03	21.89 ± 0.01
Molar activity	132.59 ± 16.11 GBq/µmol<br>3.58 ± 0.43 Ci/µmol	246.79 ± 7.77 GBq/µmol<br>6.67 ± 0.21 Ci/µmol
% Radiochemical purity	97.28 ± 2.15	98.11 ± 1.73
% Chemical purity	90.32 ± 3.08	98.28 ± 1.67

Table S7. Summary of end-of-synthesis (EOS) quality assessments for fully automated production of ^{18}F-radiotracer

Radiotracer	% Radiochemical yield (decay uncorrected)	Molar activity	% Radiochemical purity	% Chemical purity
[^{18}F]MGX-110S	21.89 ± 0.01	246.79 ± 7.77 GBq/µmol<br>6.67 ± 0.21 Ci/µmol	98.11 ± 1.73	98.28 ± 1.67
[^{18}F]MGX-111S	15.58 ± 4.28	106.56 ± 43.66 GBq/µmol<br>2.88 ± 1.18 Ci/µmol	97.59 ± 1.57	98.09 ± 2.09

H. ^{1}H, ^{19}F and ^{13}C NMR Spectra and High-Resolution Mass Spectrometry

***N*-(3-bromophenethyl)acetamide (3)**

^{1}H NMR (300 MHz, $CDCl_3$):

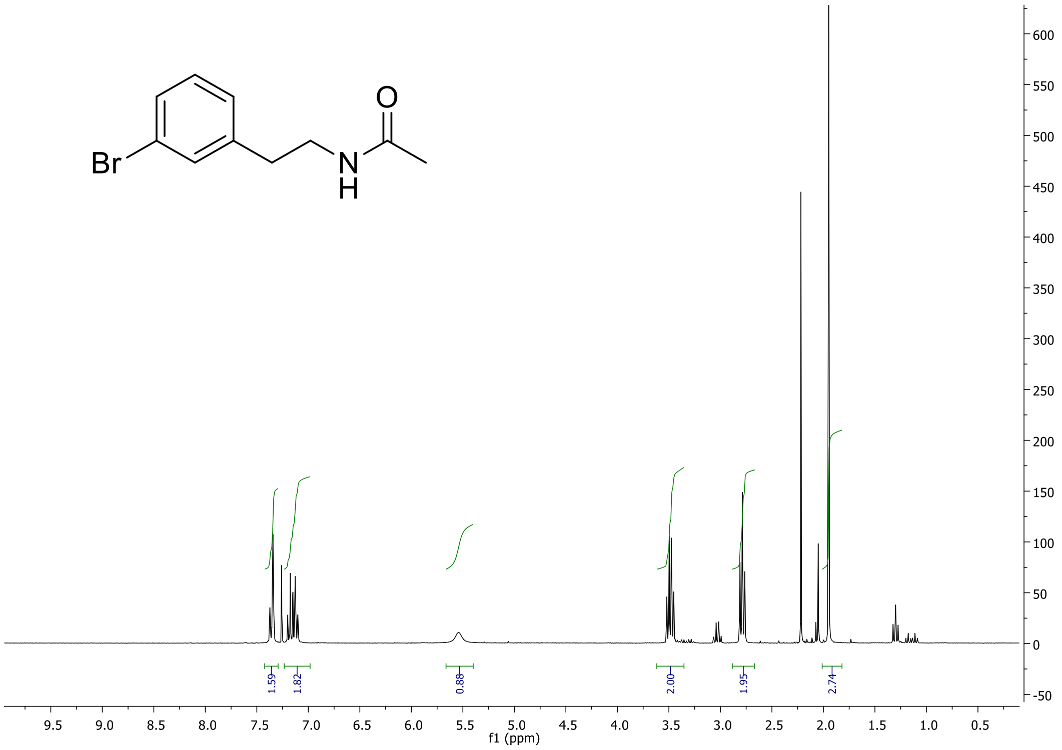

^{13}C NMR (75 MHz, $CDCl_3$):

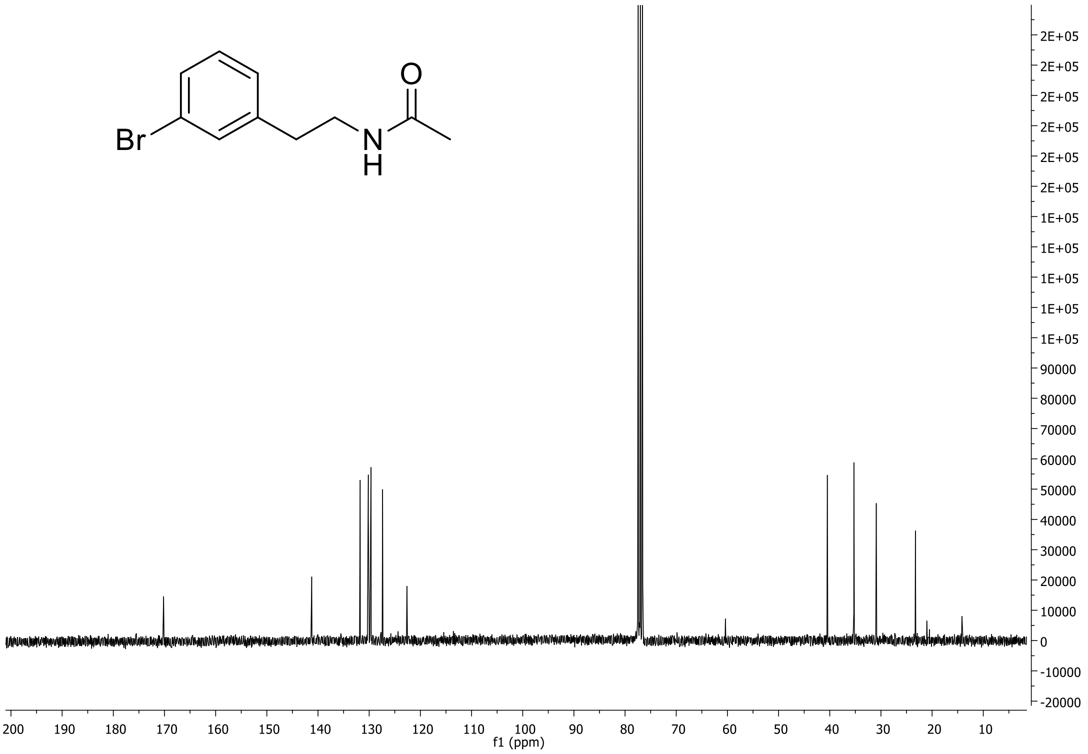

HRMS (TOF MS ES+):

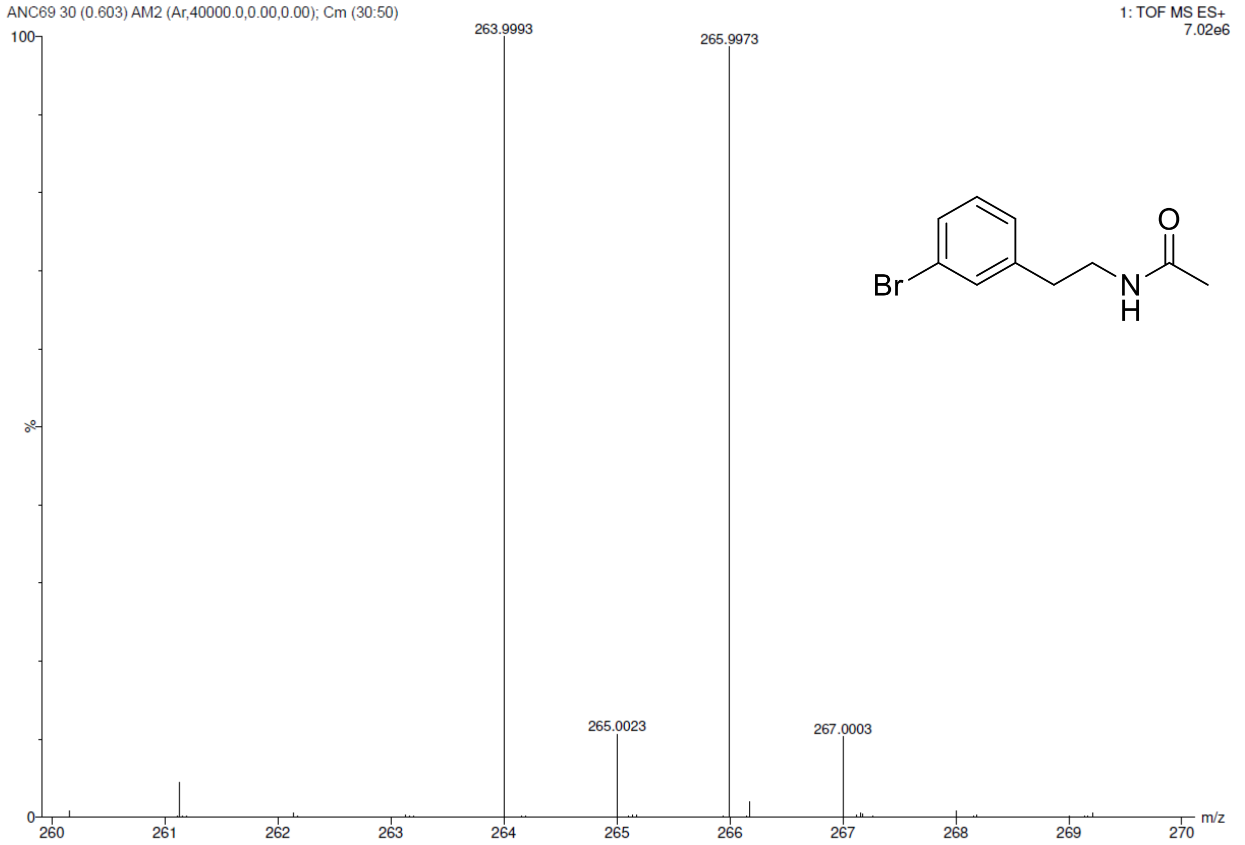

Single Mass Analysis
Tolerance = 5.0 PPM / DBE: min = -5.0, max = 300.0
Element prediction: Off
Number of isotope peaks used for i-FIT = 5

Monoisotopic Mass, Even Electron Ions
2 formula(e) evaluated with 1 results within limits (all results (up to 1000) for each mass)
Elements Used:
C: 10-10 H: 12-13 N: 1-1 O: 1-1 Na: 0-2 Br: 1-1

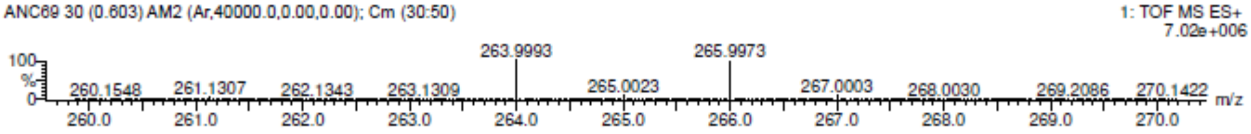

Minimum:				-5.0				
Maximum:		20.0	5.0	300.0				
Mass	Calc. Mass	mDa	PPM	DBE	i-FIT	Norm	Conf(%)	Formula
263.9993	264.0000	-0.7	-2.7	4.5	3144.3	n/a	n/a	C10 H12 N O Na Br

_N_-(3-fluorophenethyl)acetamide (4)

^{1}H NMR (300 MHz, $CDCl_3$):

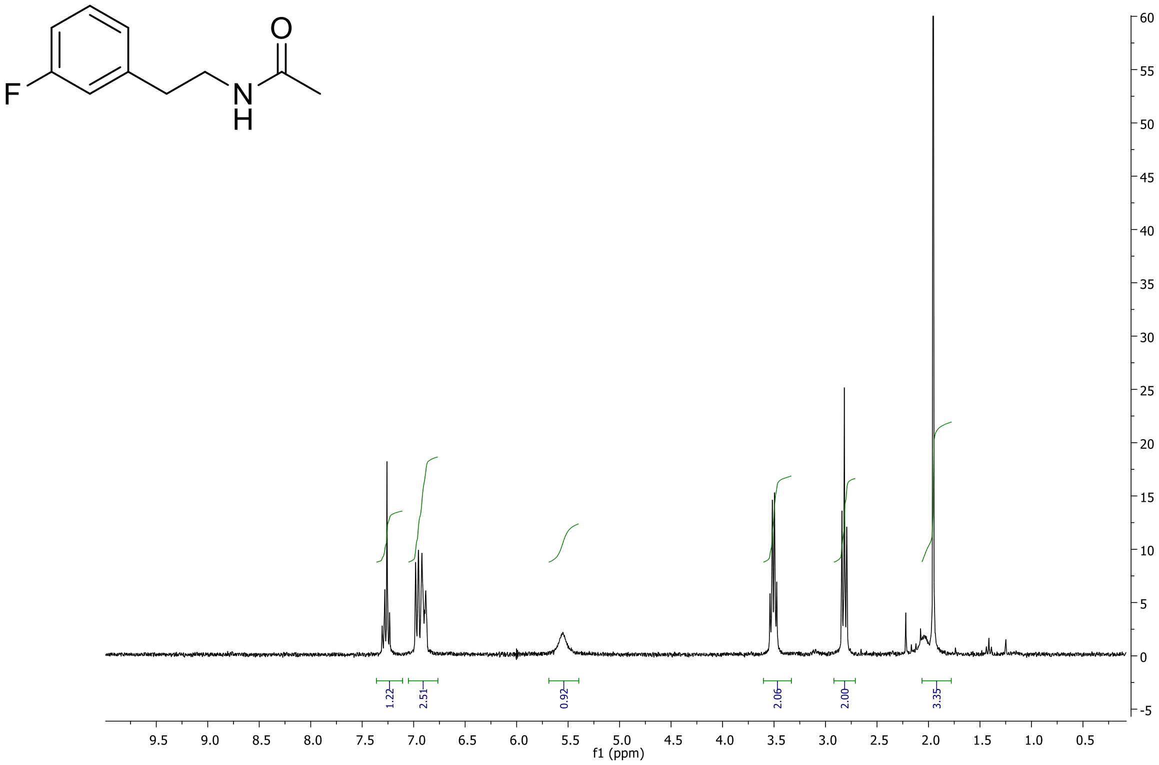

^{13}C NMR (75 MHz, $CDCl_3$):

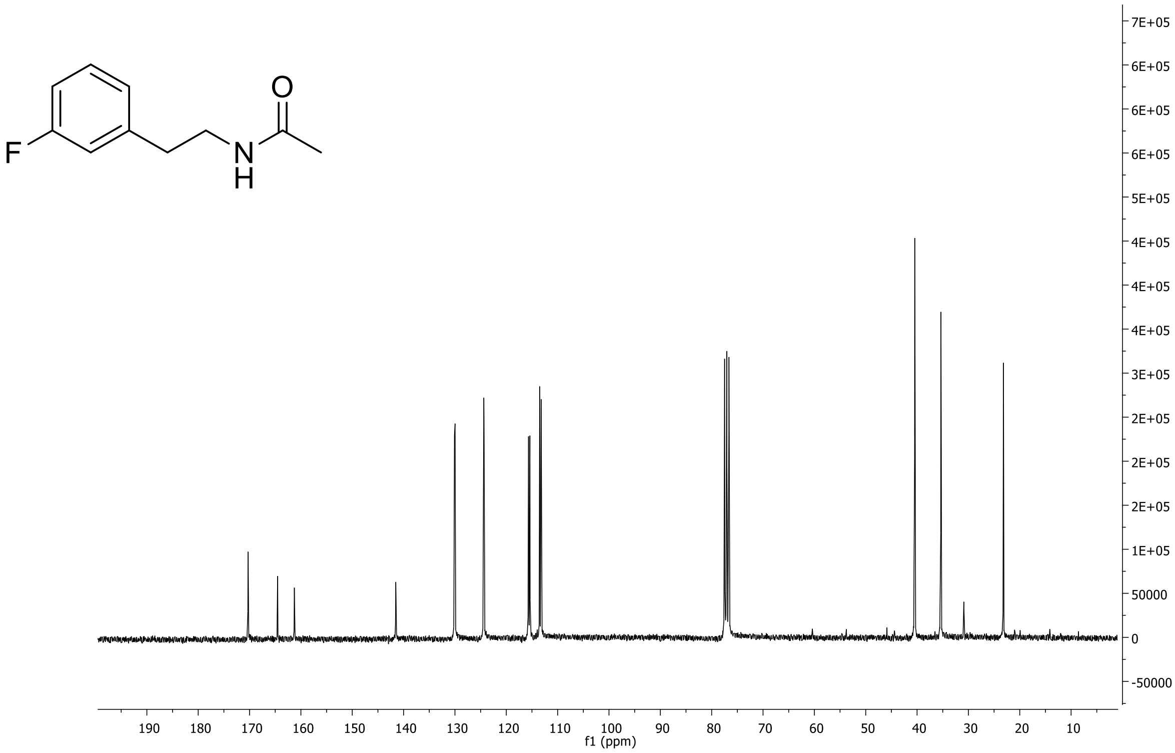

^{19}F NMR (282 MHz, $CDCl_3$):

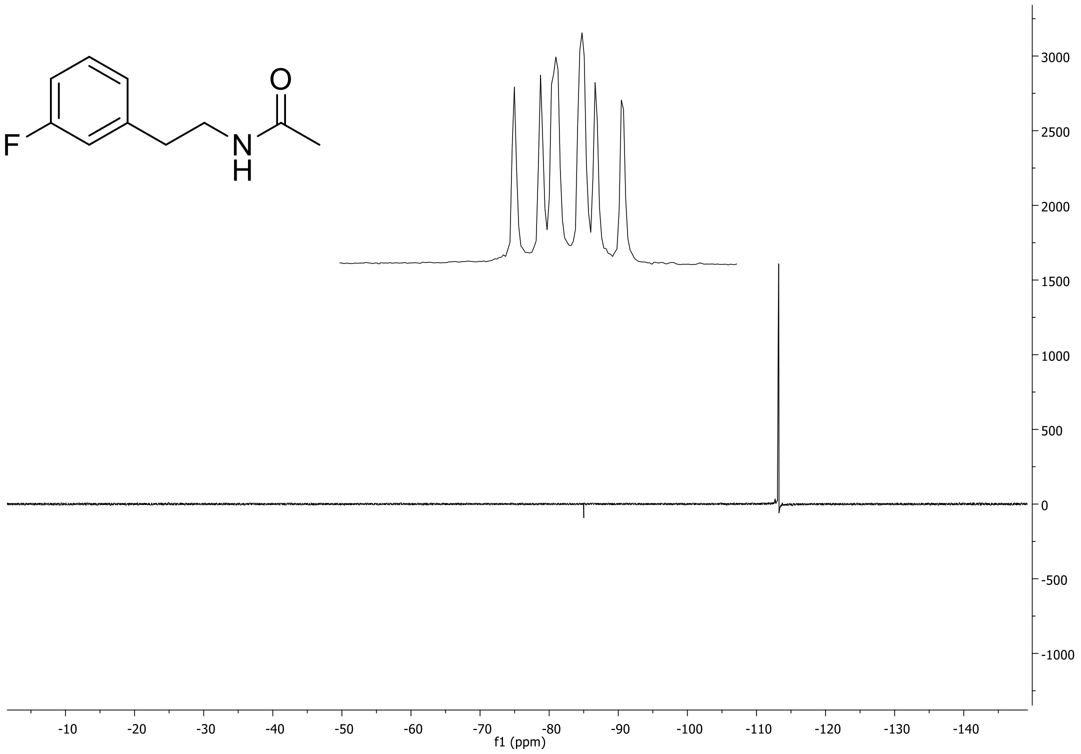

HRMS (TOF MS ES+):

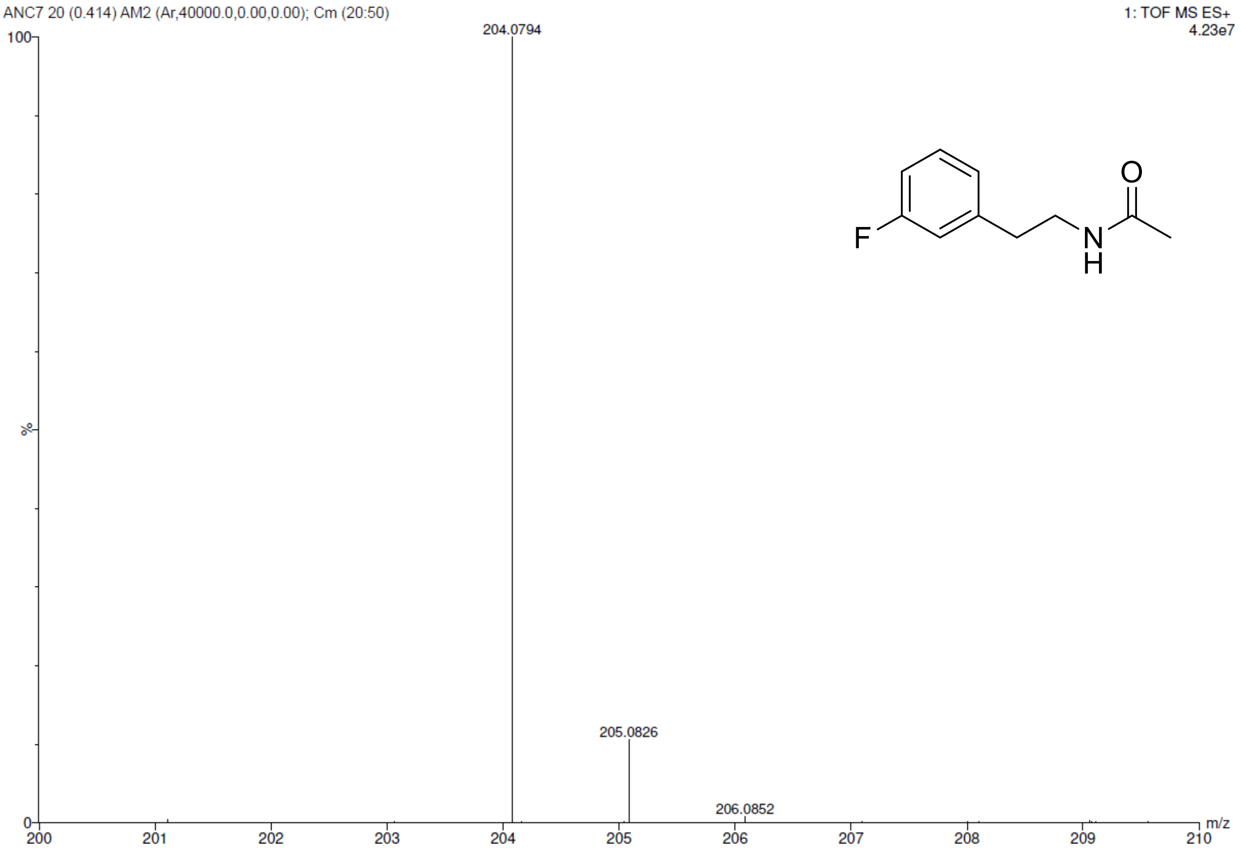

Elemental Composition Report **Page 1**

Single Mass Analysis
Tolerance = 5.0 PPM / DBE: min = -5.0, max = 300.0
Element prediction: Off
Number of isotope peaks used for i-FIT = 5

Monoisotopic Mass, Even Electron Ions
2 formula(e) evaluated with 1 results within limits (all results (up to 1000) for each mass)
Elements Used:
C: 10-10 H: 12-13 N: 1-1 O: 1-1 F: 1-1 Na: 0-2

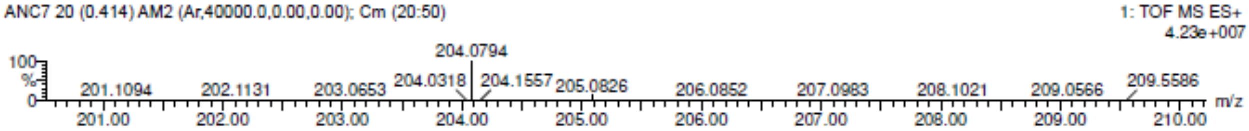

Minimum:				-5.0				
Maximum:		20.0	5.0	300.0				
Mass	Calc. Mass	mDa	PPM	DBE	i-FIT	Norm	Conf(%)	Formula
204.0794	204.0801	-0.7	-3.4	4.5	3925.4	n/a	n/a	C10 H12 N O F Na

6-bromo-1-methyl-3,4-dihydroisoquinoline (5)

^{1}H NMR (300 MHz, $CDCl_3$):

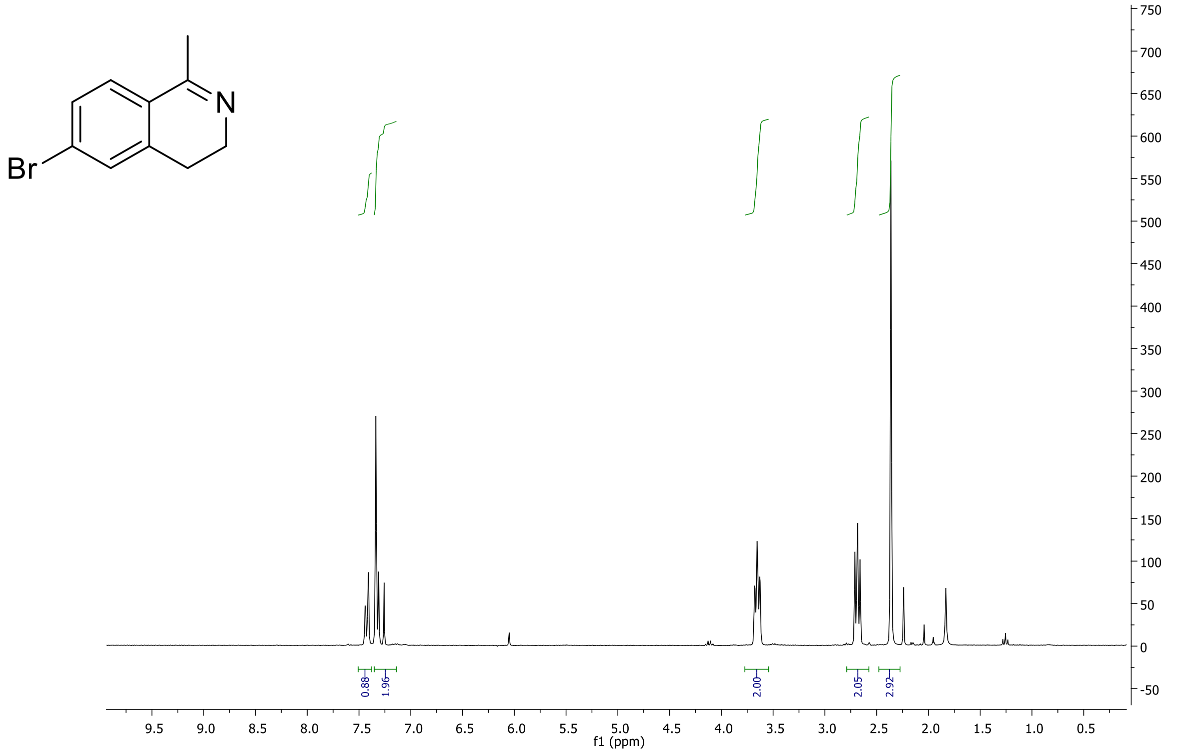

^{13}C NMR (75 MHz, $CDCl_3$):

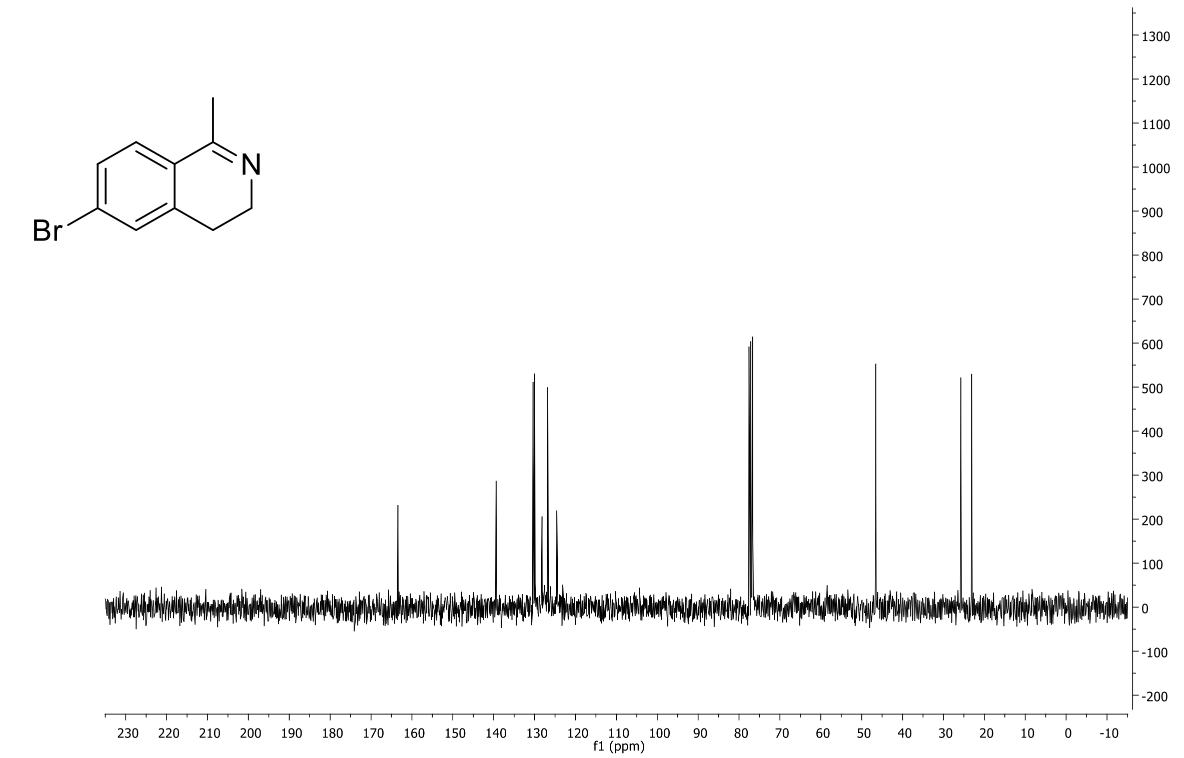

HRMS (TOF MS ES+):

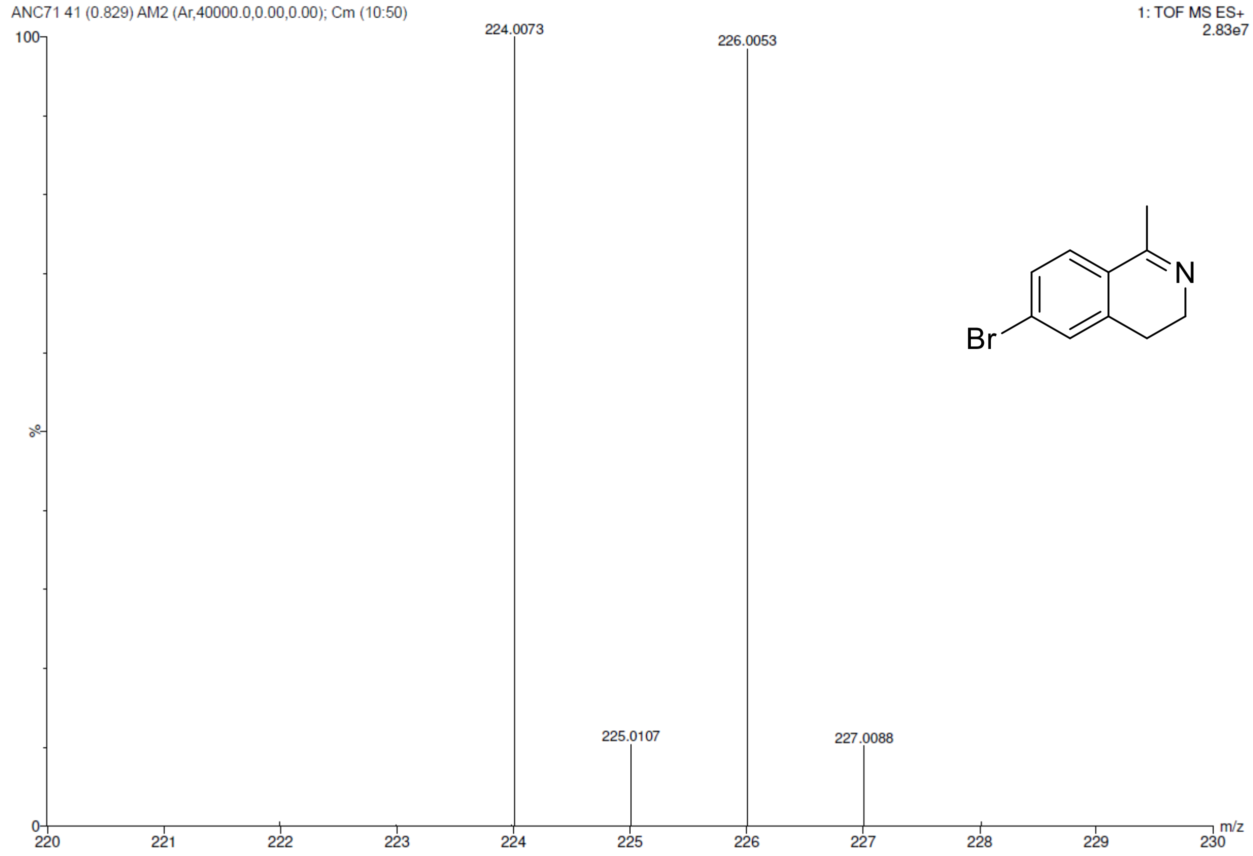

Elemental Composition Report

Page 1

Single Mass Analysis

Tolerance = 5.0 PPM / DBE: min = -5.0, max = 300.0
Element prediction: Off
Number of isotope peaks used for i-FIT = 5

Monoisotopic Mass, Even Electron Ions
3 formula(e) evaluated with 1 results within limits (all results (up to 1000) for each mass)
Elements Used:
C: 10-10 H: 10-11 N: 1-1 Na: 0-2 Br: 1-1

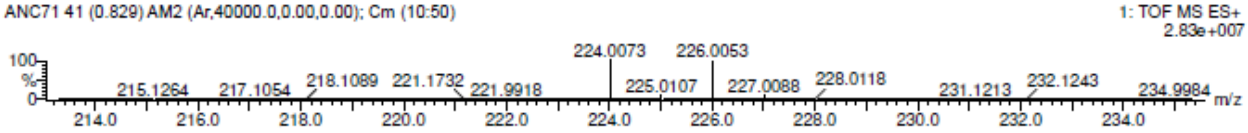

Minimum:				-5.0				
Maximum:		20.0	5.0	300.0				
Mass	Calc. Mass	mDa	PPM	DBE	i-FIT	Norm	Conf(%)	Formula
224.0073	224.0075	-0.2	-0.9	5.5	3964.4	n/a	n/a	C10 H11 N Br

6-fluoro-1-methyl-3,4-dihydroisoquinoline (6)

^{1}H NMR (300 MHz, $CDCl_3$):

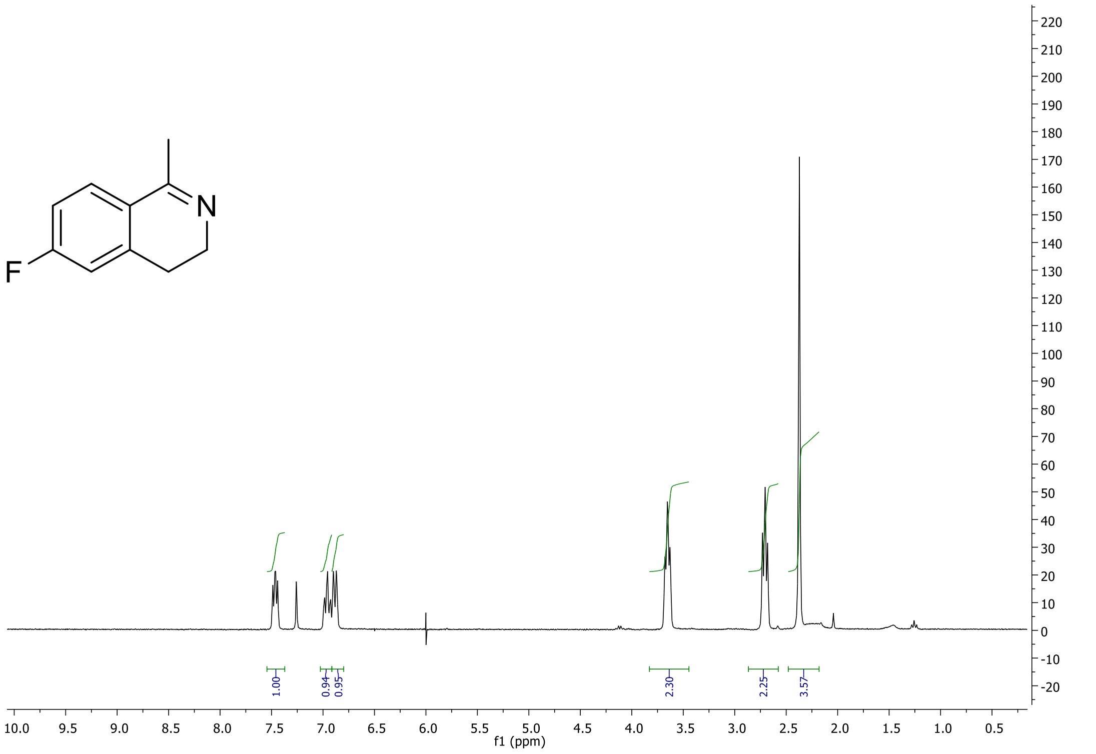

^{13}C NMR (75 MHz, $CDCl_3$):

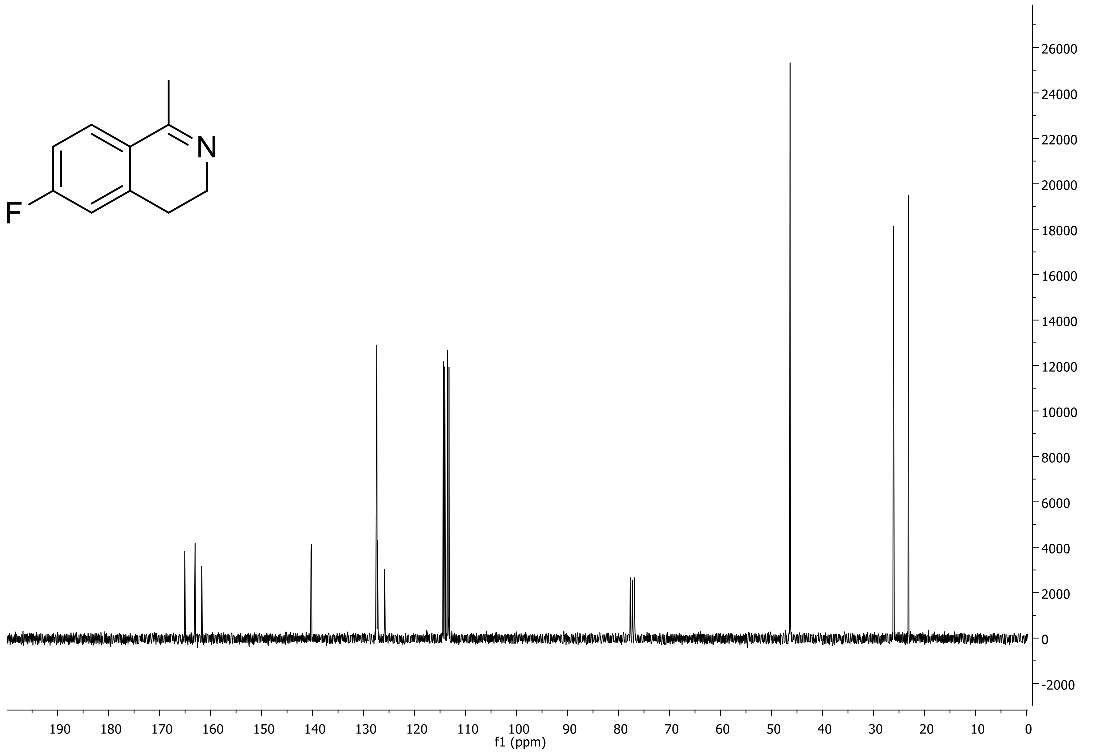

^{19}F NMR (282 MHz, $CDCl_3$):

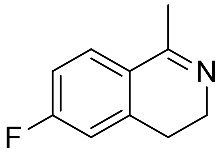

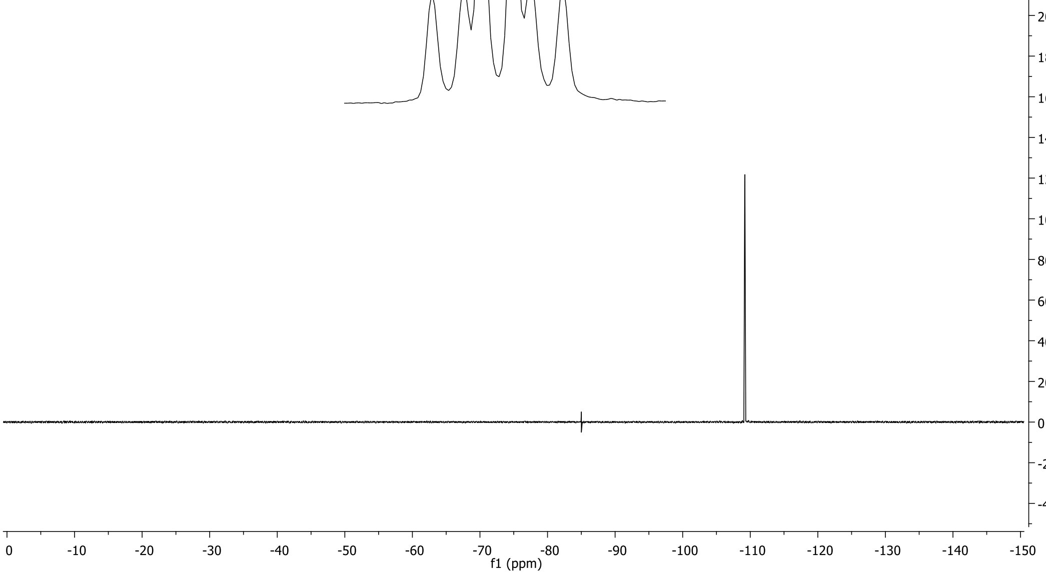

HRMS (TOF MS ES+):

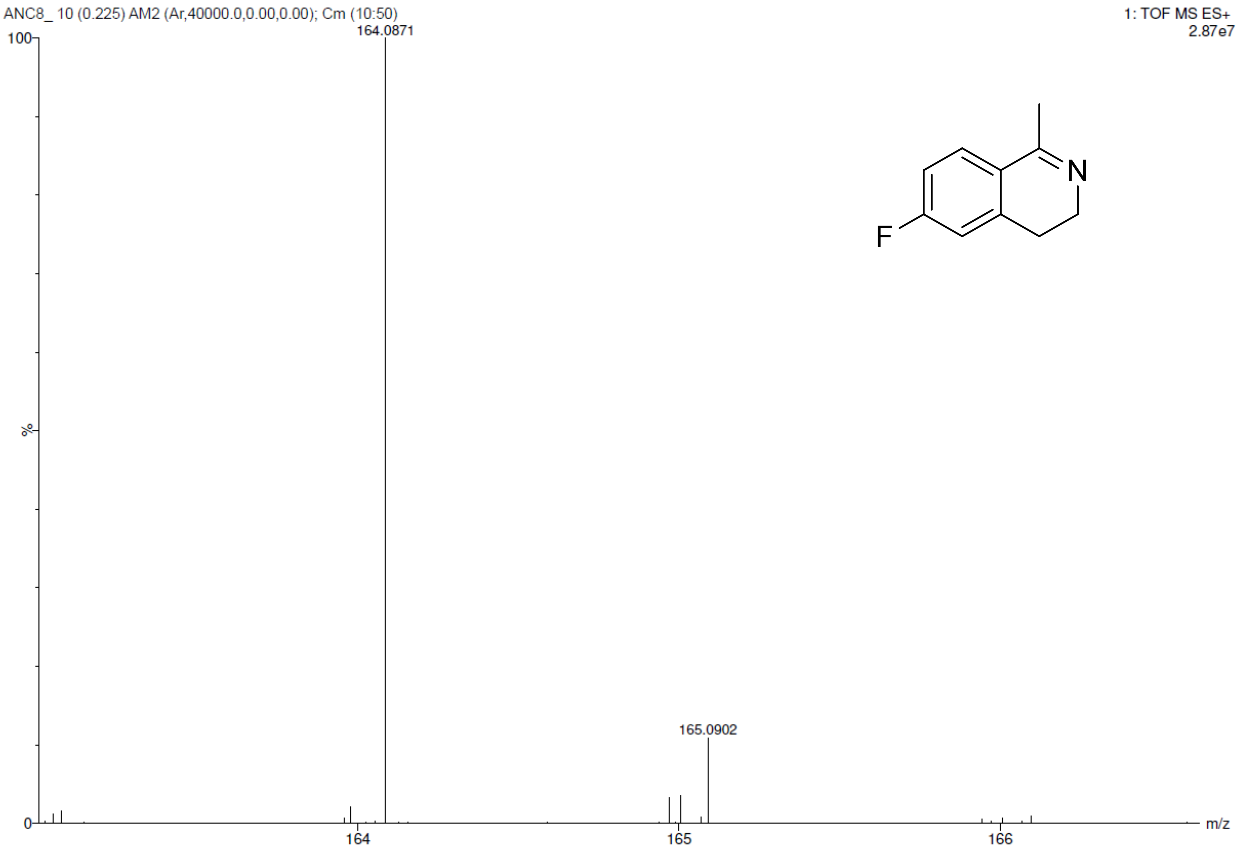

Elemental Composition Report

Page 1

Single Mass Analysis

Tolerance = 5.0 PPM / DBE: min = -5.0, max = 300.0
Element prediction: Off
Number of isotope peaks used for i-FIT = 5

Monoisotopic Mass, Even Electron Ions
3 formula(e) evaluated with 1 results within limits (all results (up to 1000) for each mass)
Elements Used:
C: 10-10 H: 10-11 N: 1-1 F: 1-1 Na: 0-2

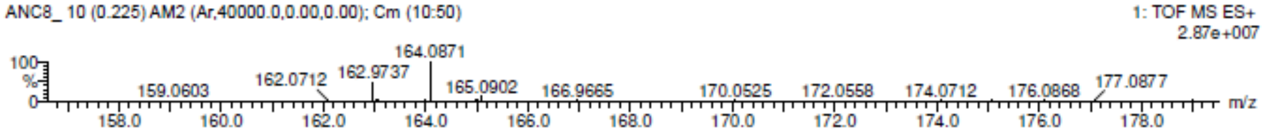

Minimum:				-5.0				
Maximum:		20.0	5.0	300.0				
Mass	Calc. Mass	mDa	PPM	DBE	i-FIT	Norm	Conf(%)	Formula
164.0871	164.0876	-0.5	-3.0	5.5	4897.3	n/a	n/a	C10 H11 N F

Benzoyl isocyanate (B)

^1H NMR (300 MHz, $CDCl_3$):

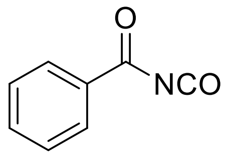

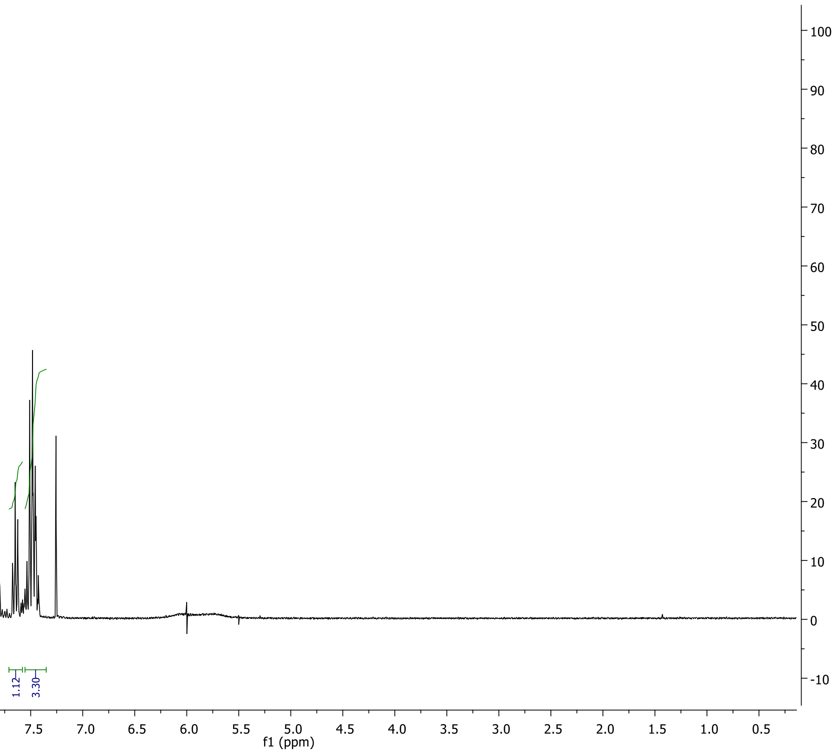

9-bromo-6,7-dihydropyrimido[6,1-*a*]isoquinoline-2,4-dione (7)

^{1}H NMR (300 MHz, DMSO-d_6):

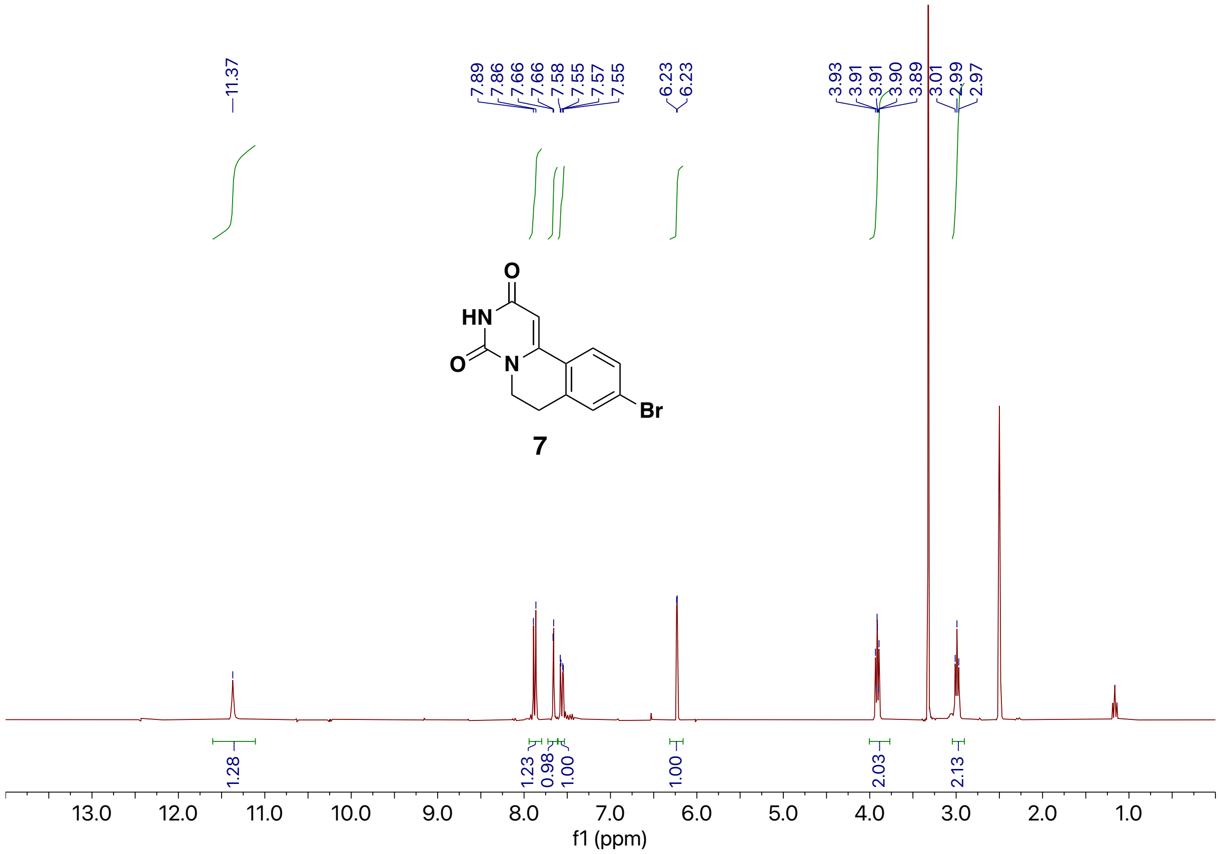

^{13}C NMR (75 MHz, DMSO-d_6):

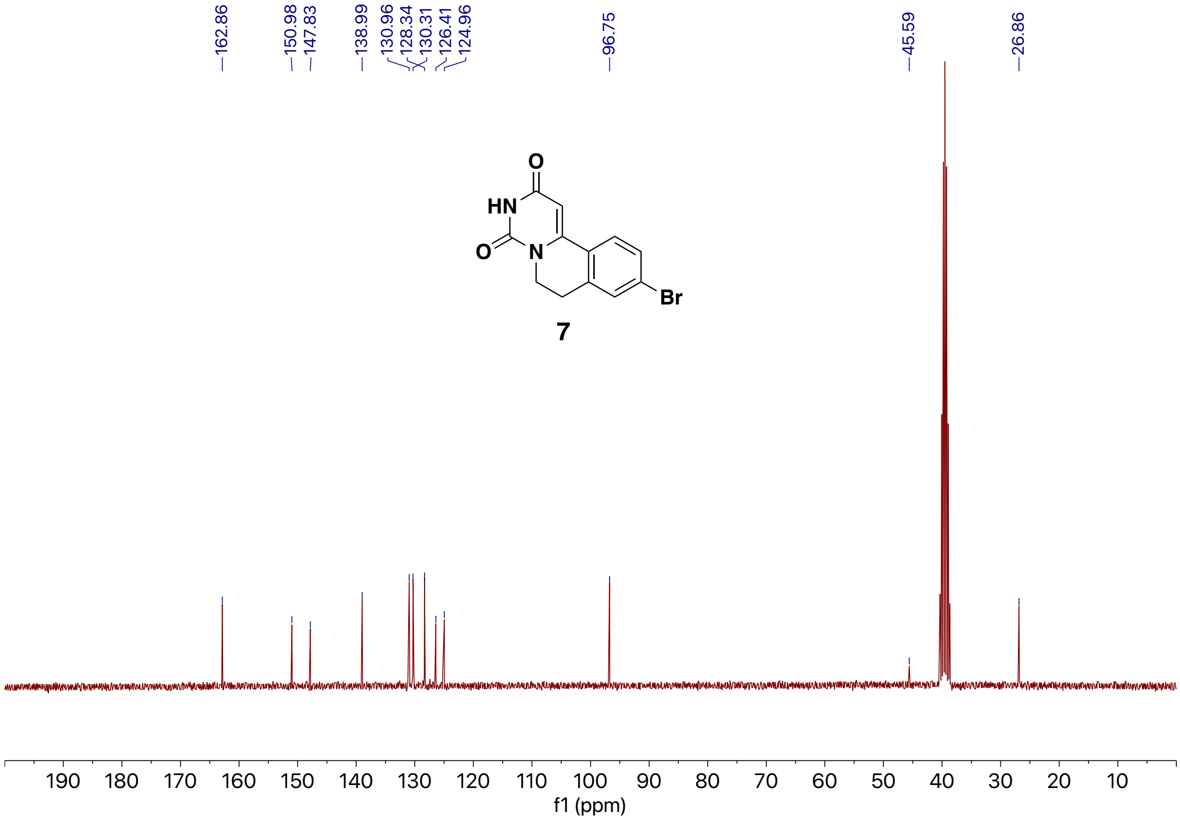

HRMS (TOF MS ES+):

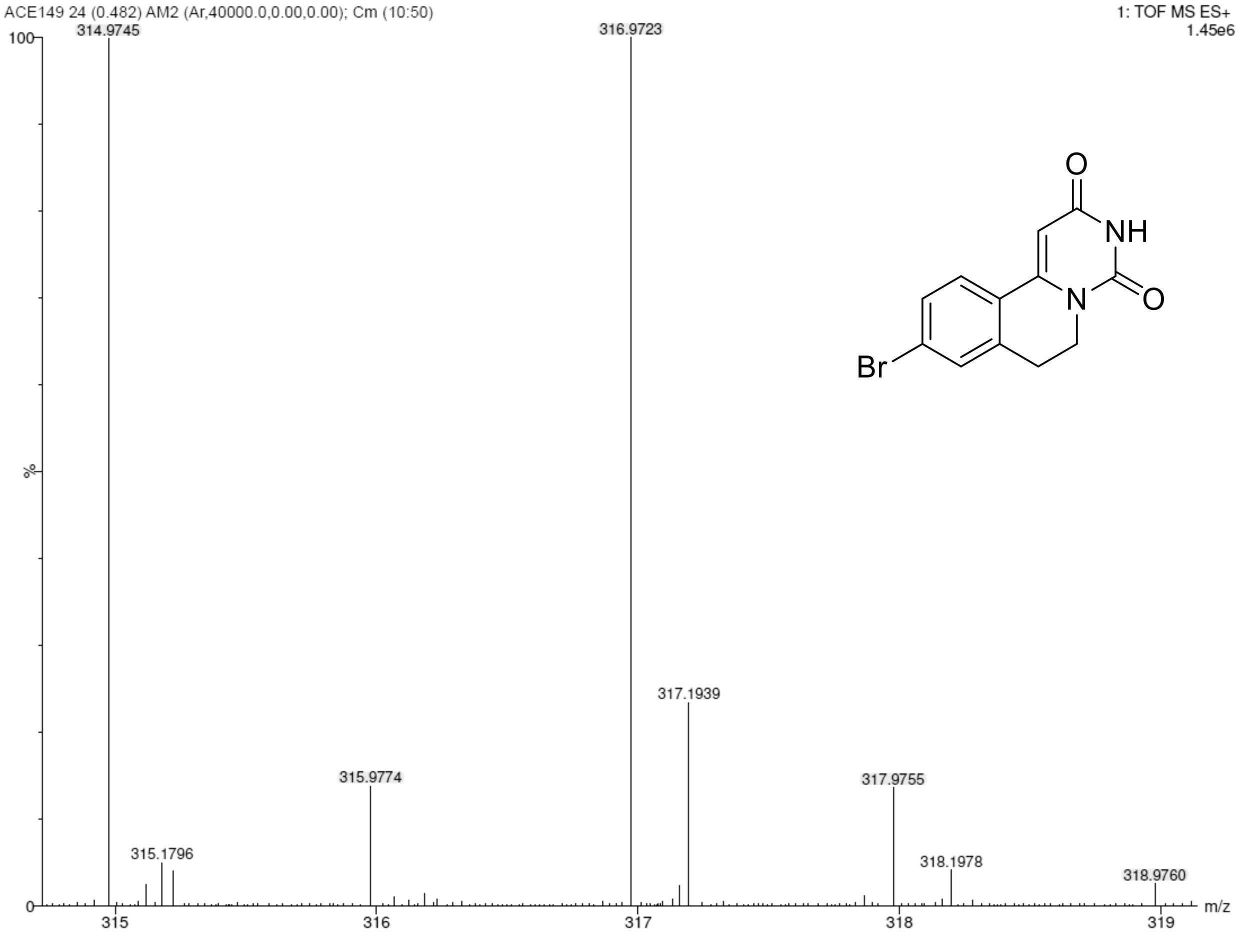

Elemental Composition Report

Page 1

Single Mass Analysis

Tolerance = 5.0 PPM / DBE: min = -5.0, max = 300.0
Element prediction: Off
Number of isotope peaks used for i-FIT = 7

Monoisotopic Mass, Even Electron Ions
4 formula(e) evaluated with 1 results within limits (all results (up to 1000) for each mass)
Elements Used:
C: 12-12 H: 9-9 N: 2-2 O: 2-2 Na: 0-4 Br: 1-1

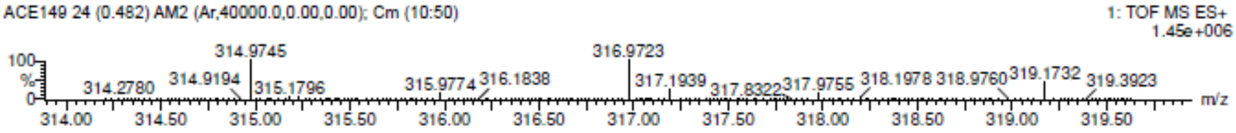

Minimum:				-5.0				
Maximum:		5.0	5.0	300.0				
Mass	Calc. Mass	mDa	PPM	DBE	i-FIT	Norm	Conf(%)	Formula
314.9745	314.9745	0.0	0.0	8.5	3076.0	n/a	n/a	C12 H9 N2 O2 Na Br

9-fluoro-6,7-dihydropyrimido[6,1-*a*]isoquinoline-2,4-dione (8)

^{1}H NMR (300 MHz, DMSO-d$_6$):

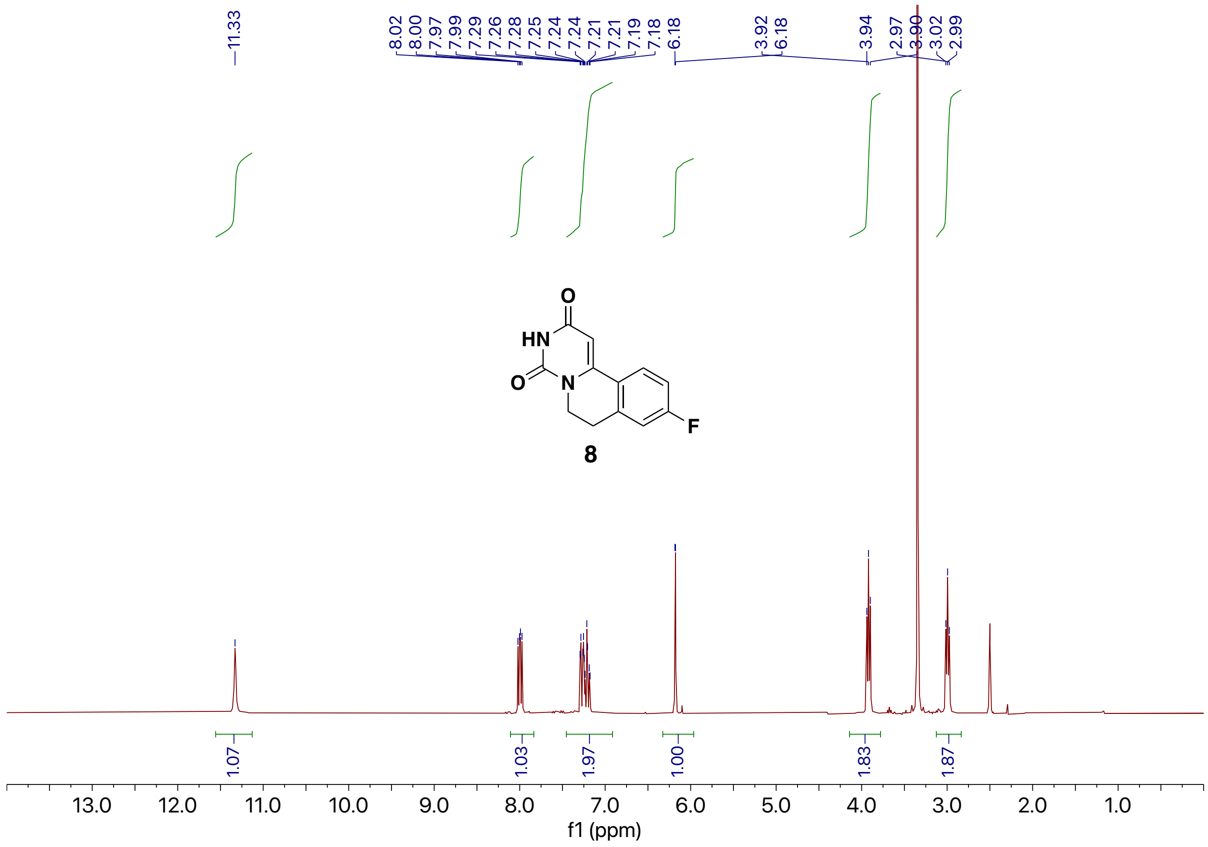

^{13}C NMR (75 MHz, DMSO-d$_6$):

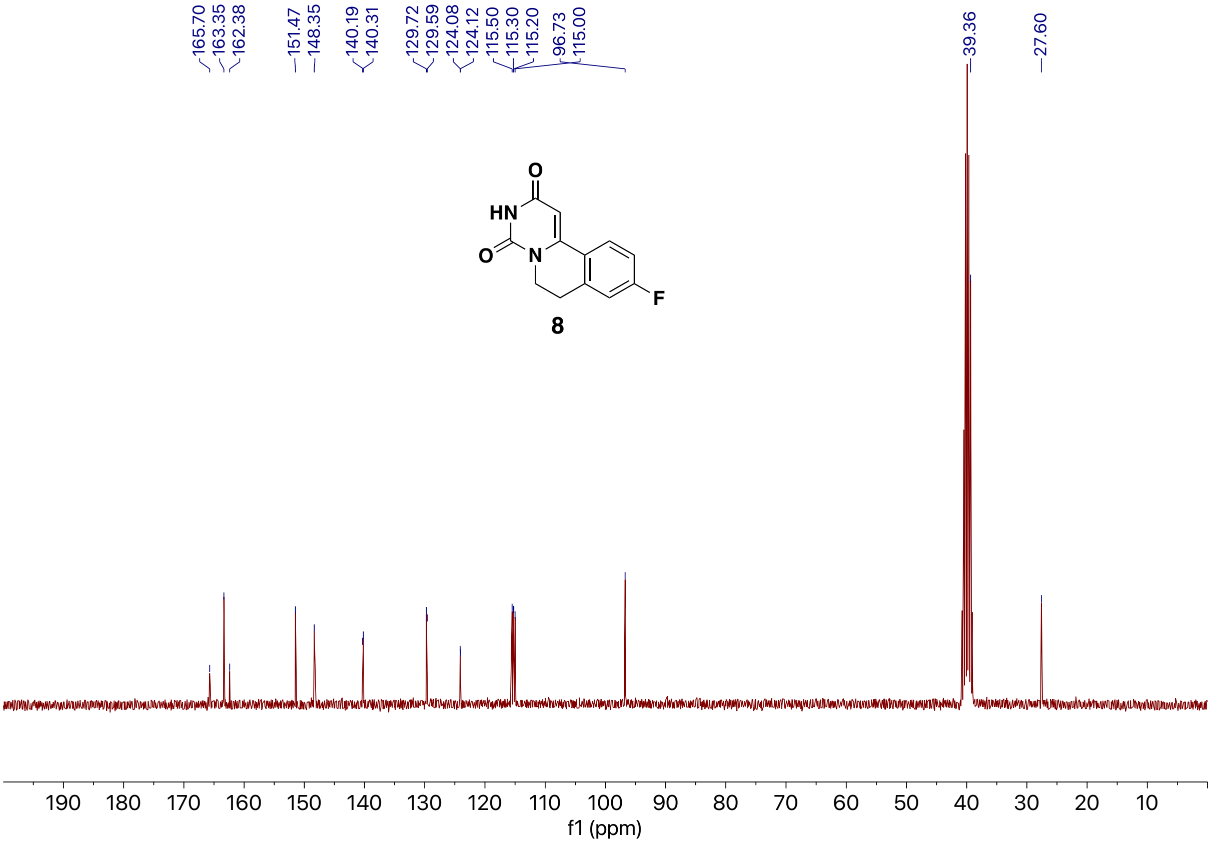

^{19}F NMR (282 MHz, DMSO-d$_6$):

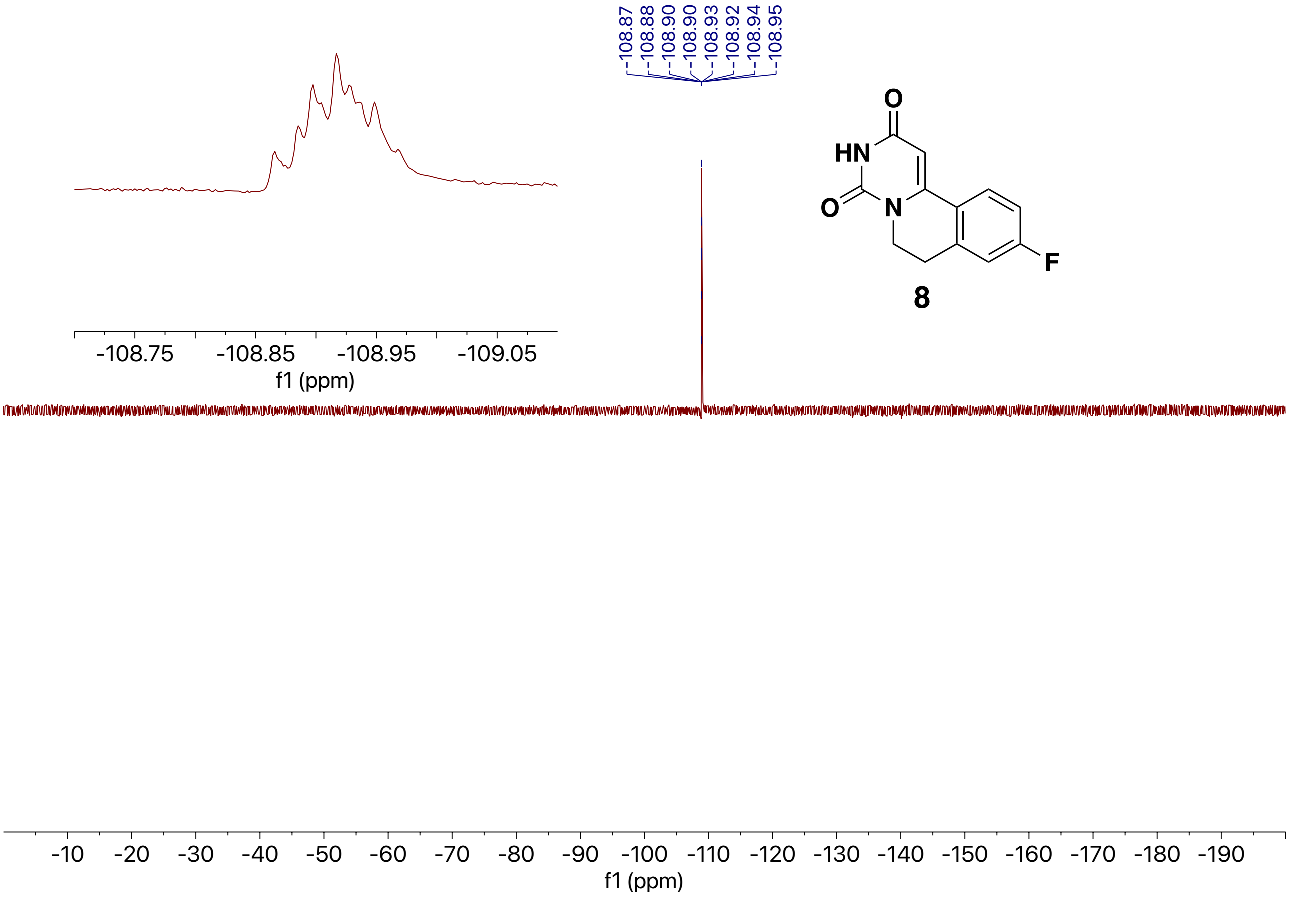

HRMS (TOF MS ES+):

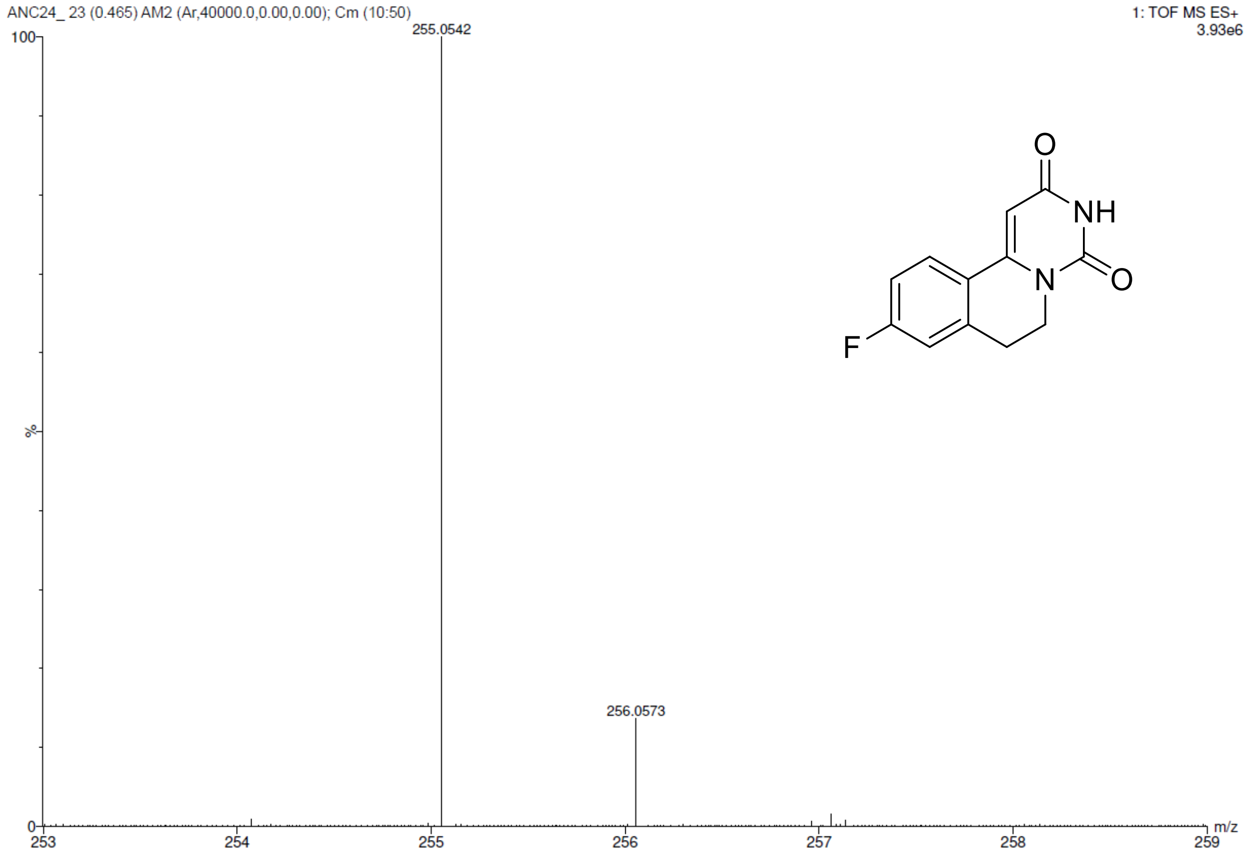

Elemental Composition Report

Page 1

Single Mass Analysis

Tolerance = 5.0 PPM / DBE: min = -5.0, max = 300.0
Element prediction: Off
Number of isotope peaks used for i-FIT = 7

Monoisotopic Mass, Even Electron Ions
2 formula(e) evaluated with 1 results within limits (all results (up to 1000) for each mass)
Elements Used:
C: 12-12 H: 9-10 N: 2-2 O: 2-2 Na: 0-2 F: 1-1

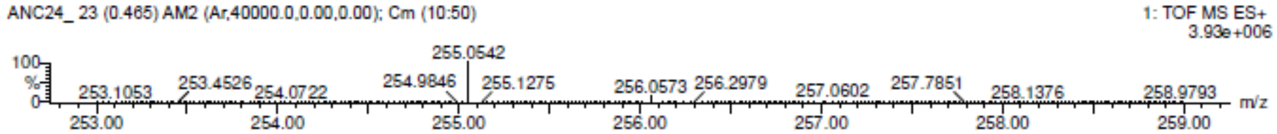

Mass	Calc. Mass	mDa	PPM	DBE	i-FIT	Norm	Conf(%)	Formula
Minimum:				-5.0				
Maximum:		20.0	5.0	300.0				
255.0542	255.0546	-0.4	-1.6	8.5	3058.1	n/a	n/a	C12 H9 N2 O2 Na F

9-bromo-2-chloro-6,7-dihydropyrimido[6,1-*a*]isoquinolin-4-one (9)

^{1}H NMR (400 MHz, DMSO-d_6):

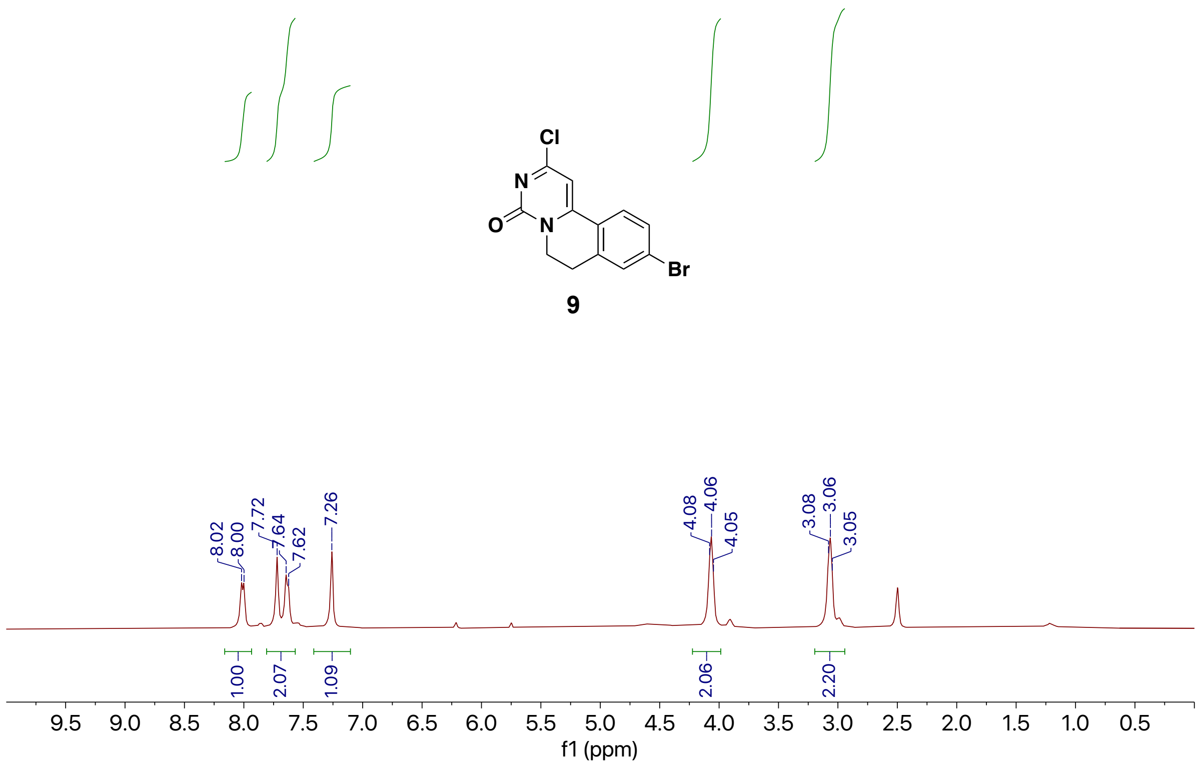

^{13}C NMR (400 MHz, DMSO-d_6):

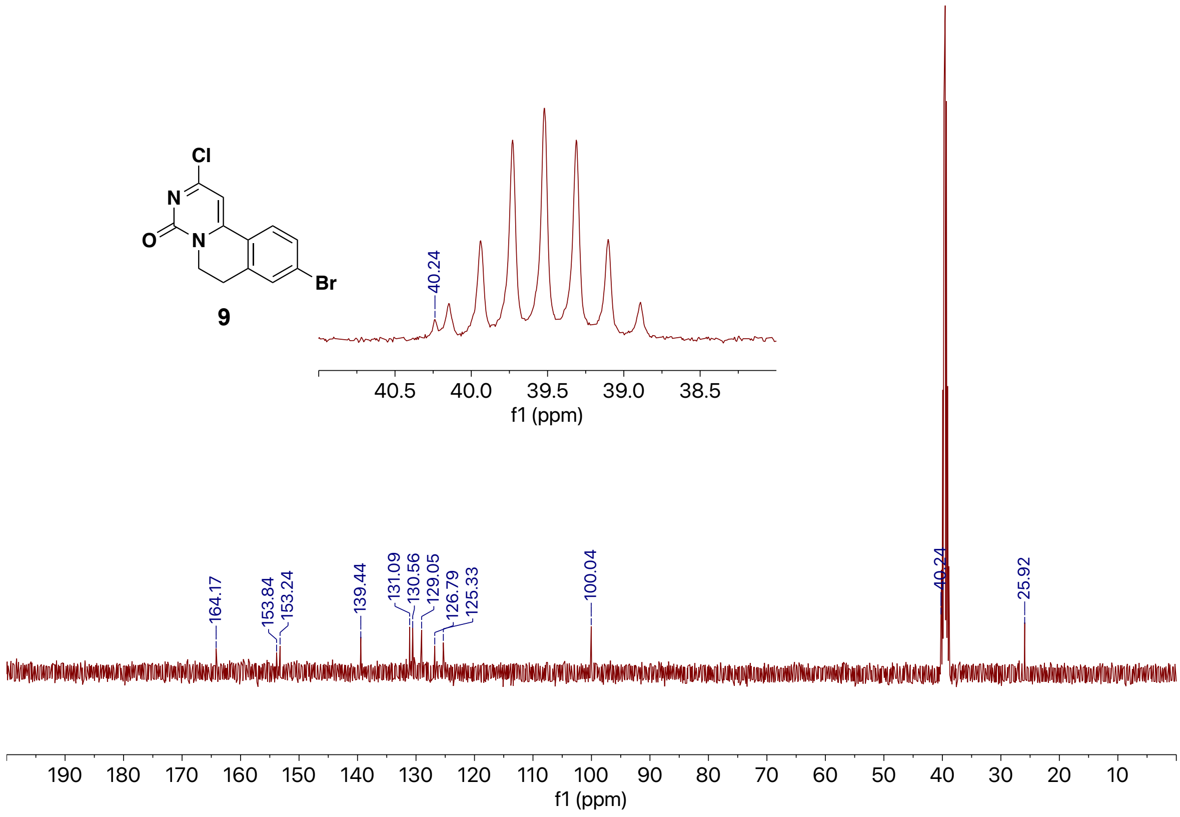

HRMS (ESI positive mode):

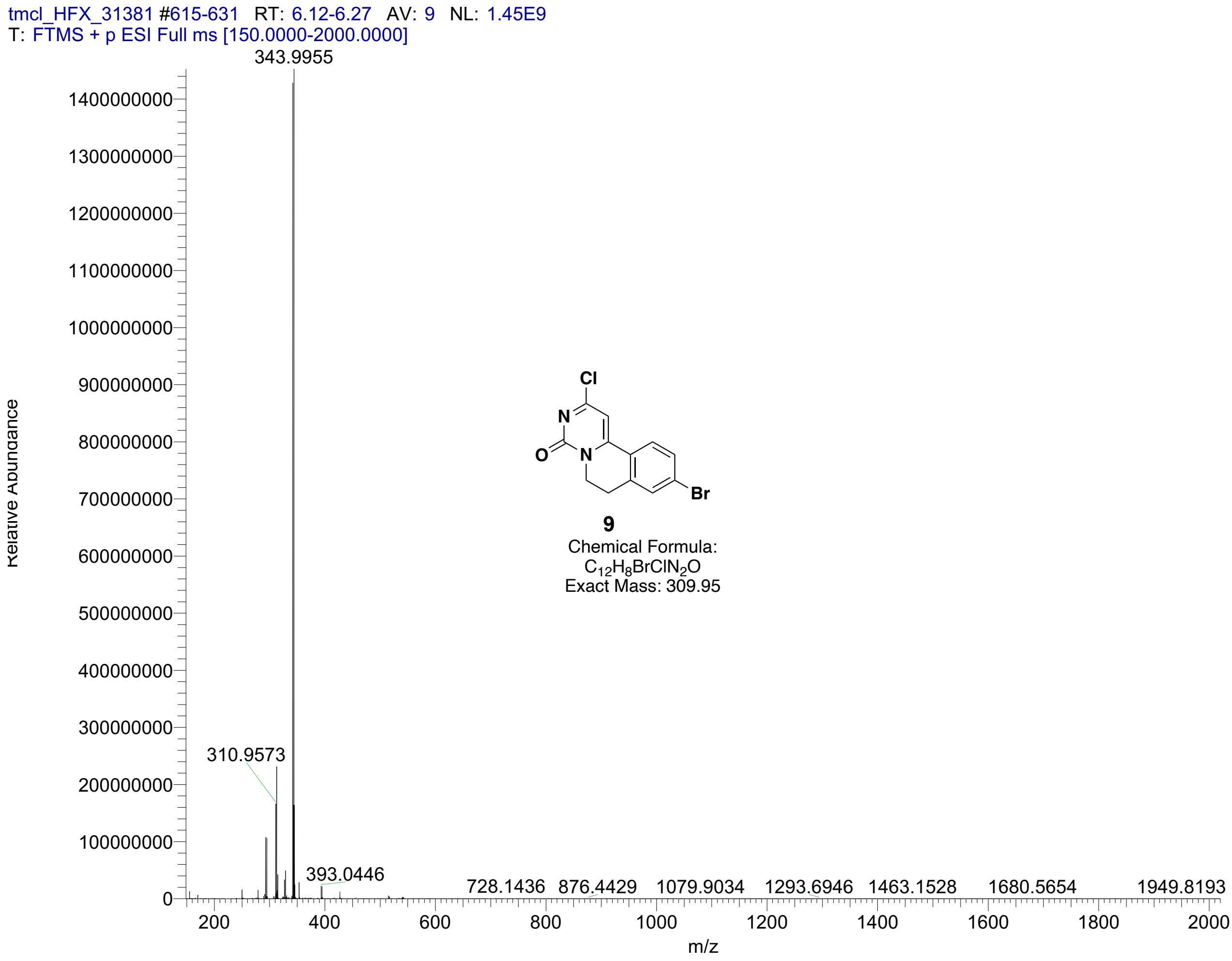

9-fluoro-2-chloro-6,7-dihydropyrimido[6,1-*a*]isoquinolin-4-one (10)

^{1}H NMR (400 MHz, Chloroform-*d*):

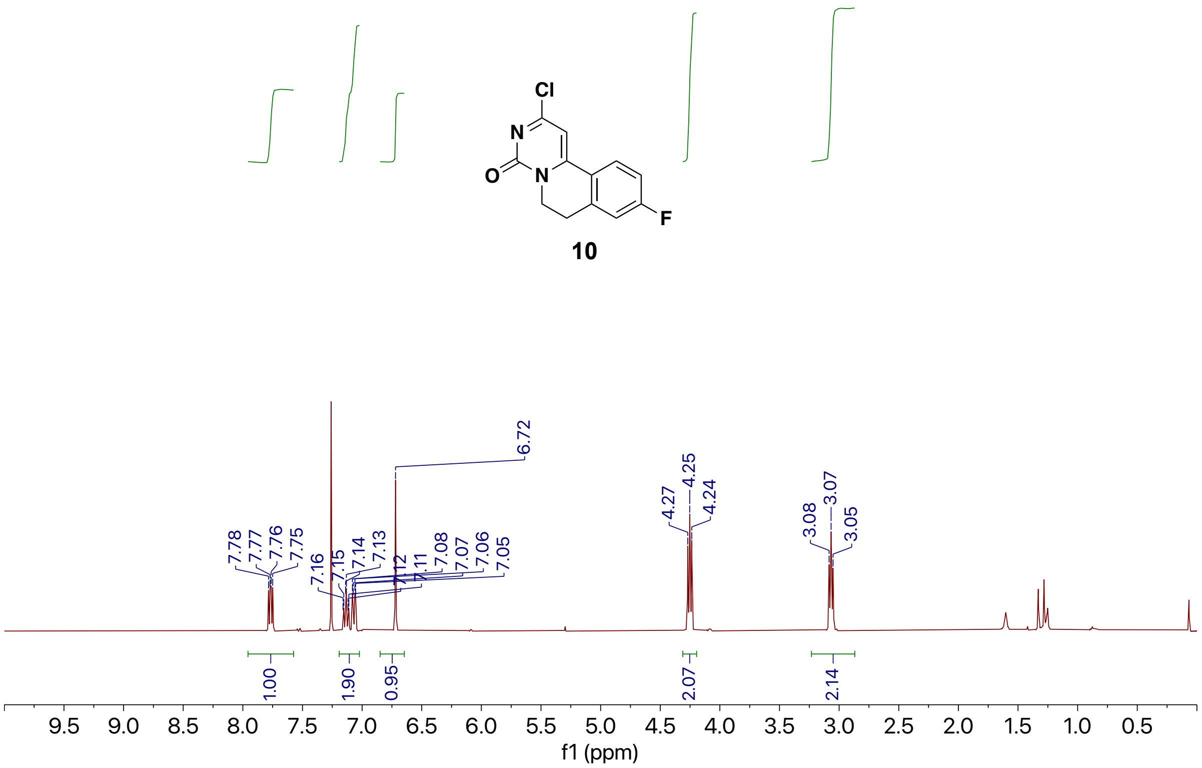

^{13}C NMR (400 MHz, Chloroform-*d*):

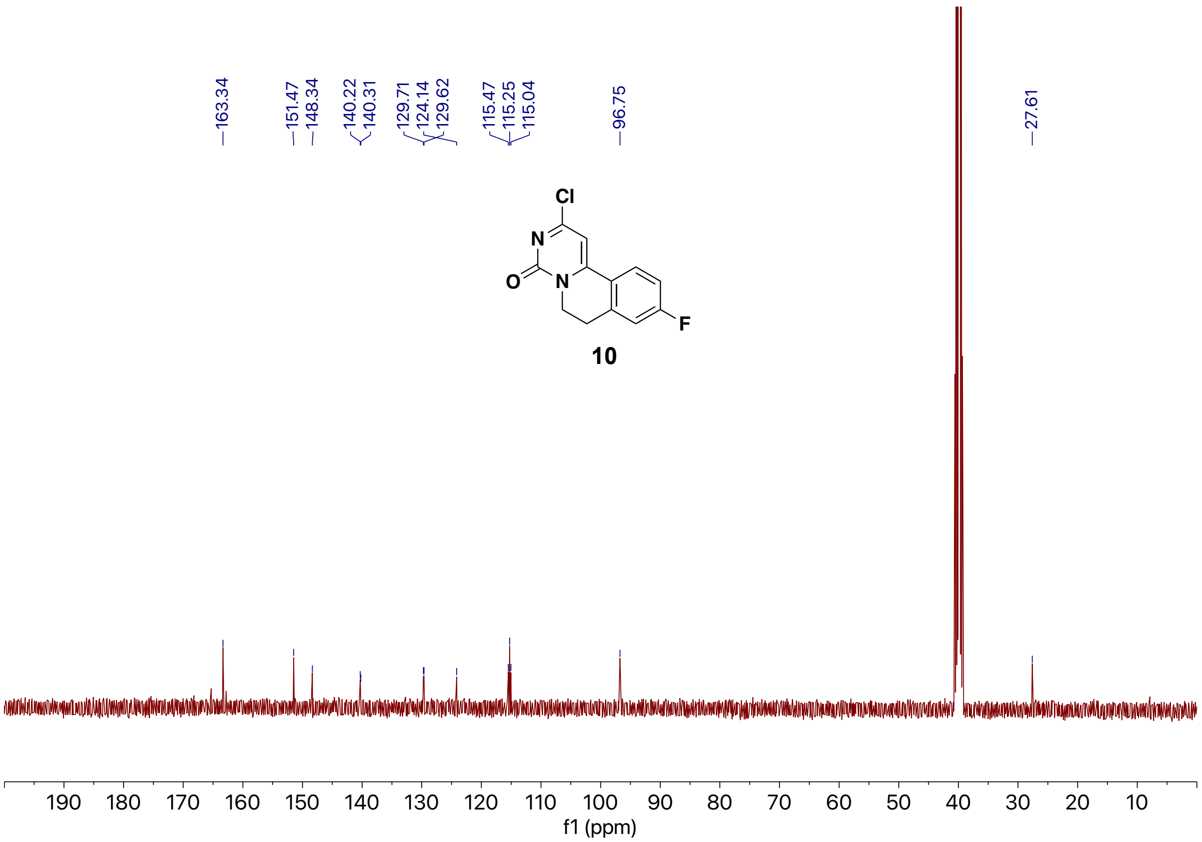

^{19}F NMR (400 MHz, Chloroform-*d*):

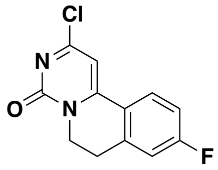

10

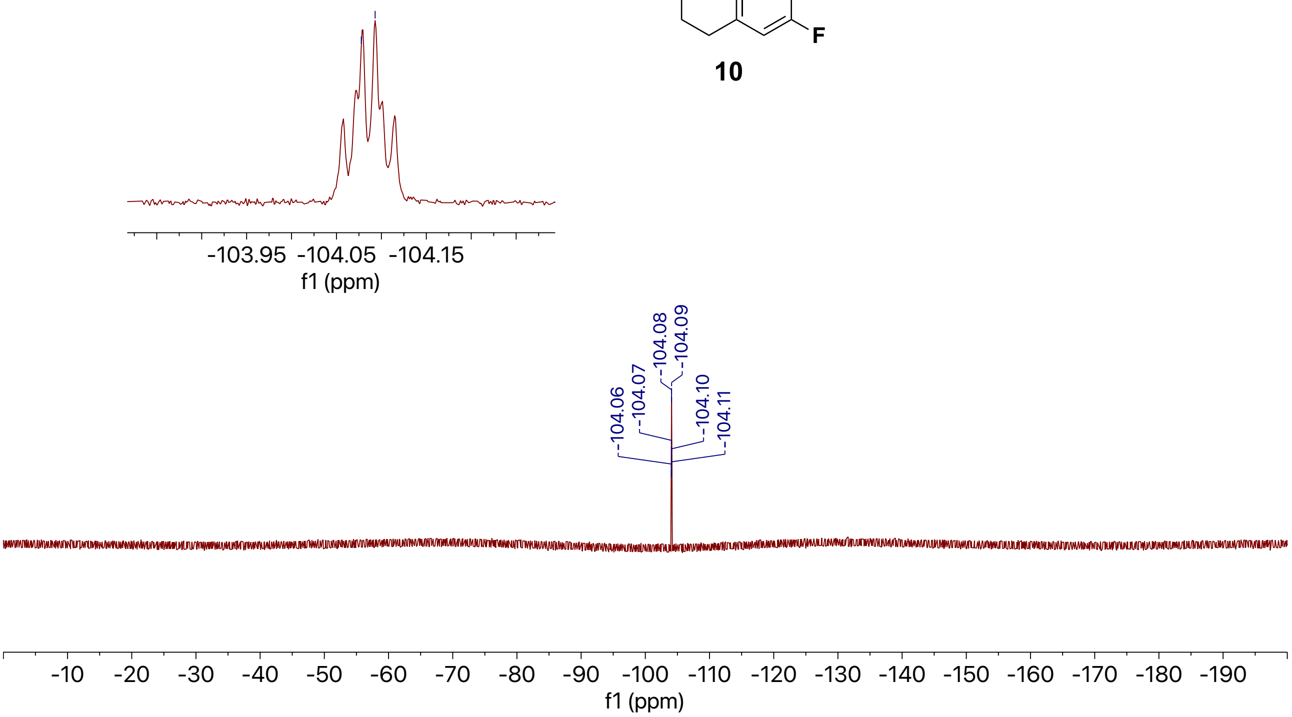

HRMS (ESI positive mode):

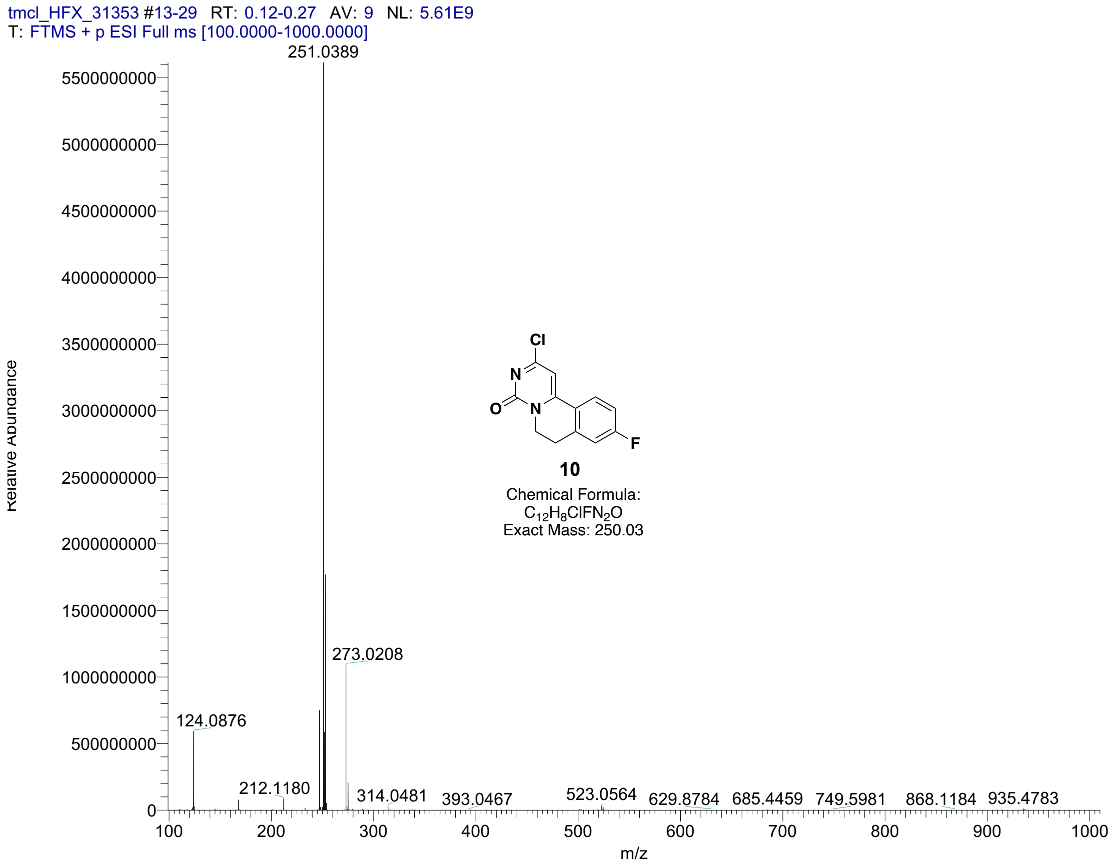

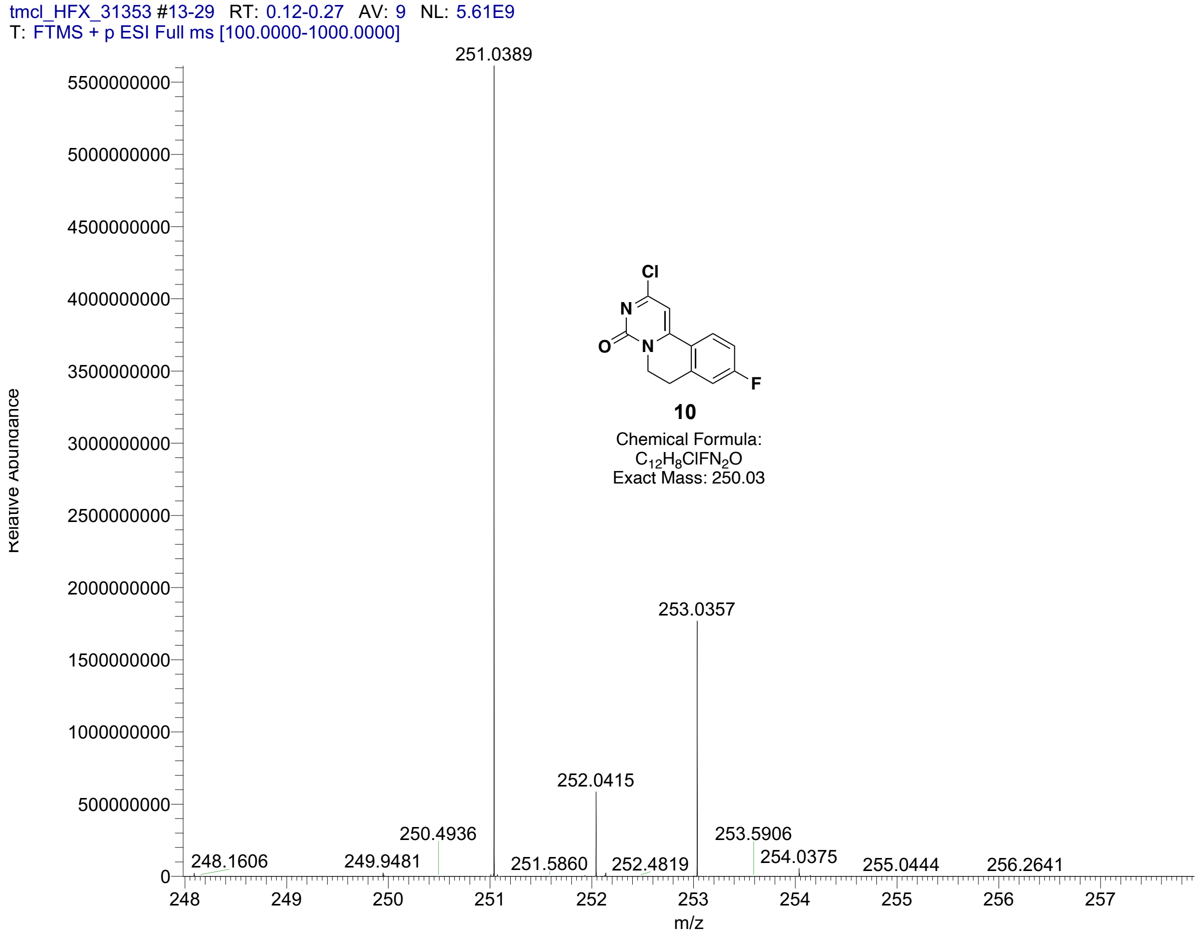

9-bromo-2-[[(2S)-1,4-dioxan-2-yl]methoxy]-6,7-dihydropyrimido[6,1-*a*]isoquinolin-4-one (11)

^{1}H NMR (400 MHz, Chloroform-*d*):

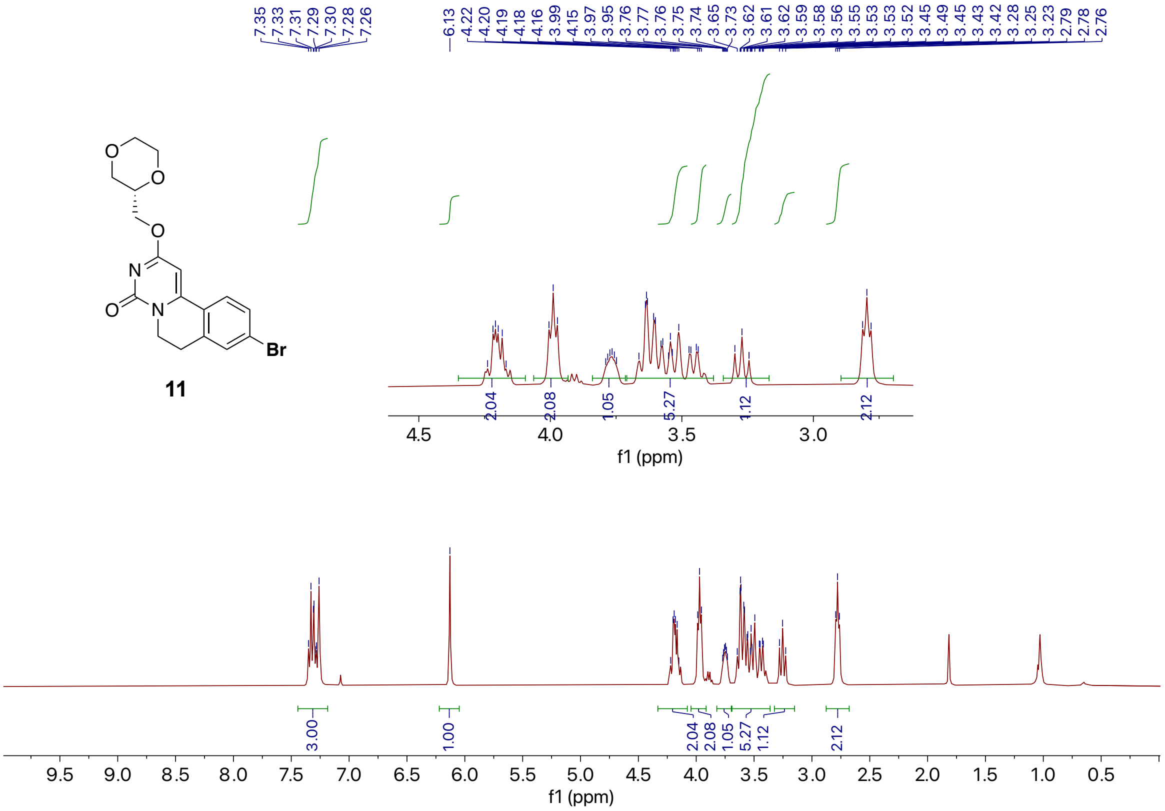

^{13}C NMR (400 MHz, Chloroform-*d*):

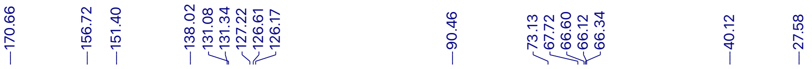

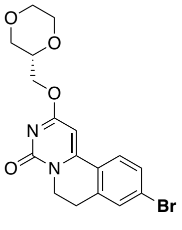

11

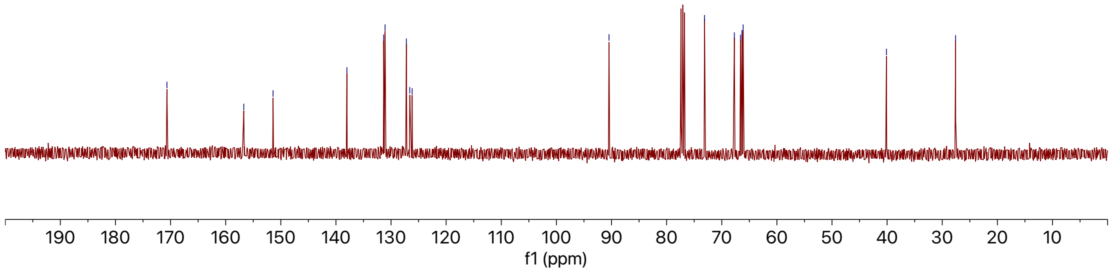

HRMS (ESI positive mode):

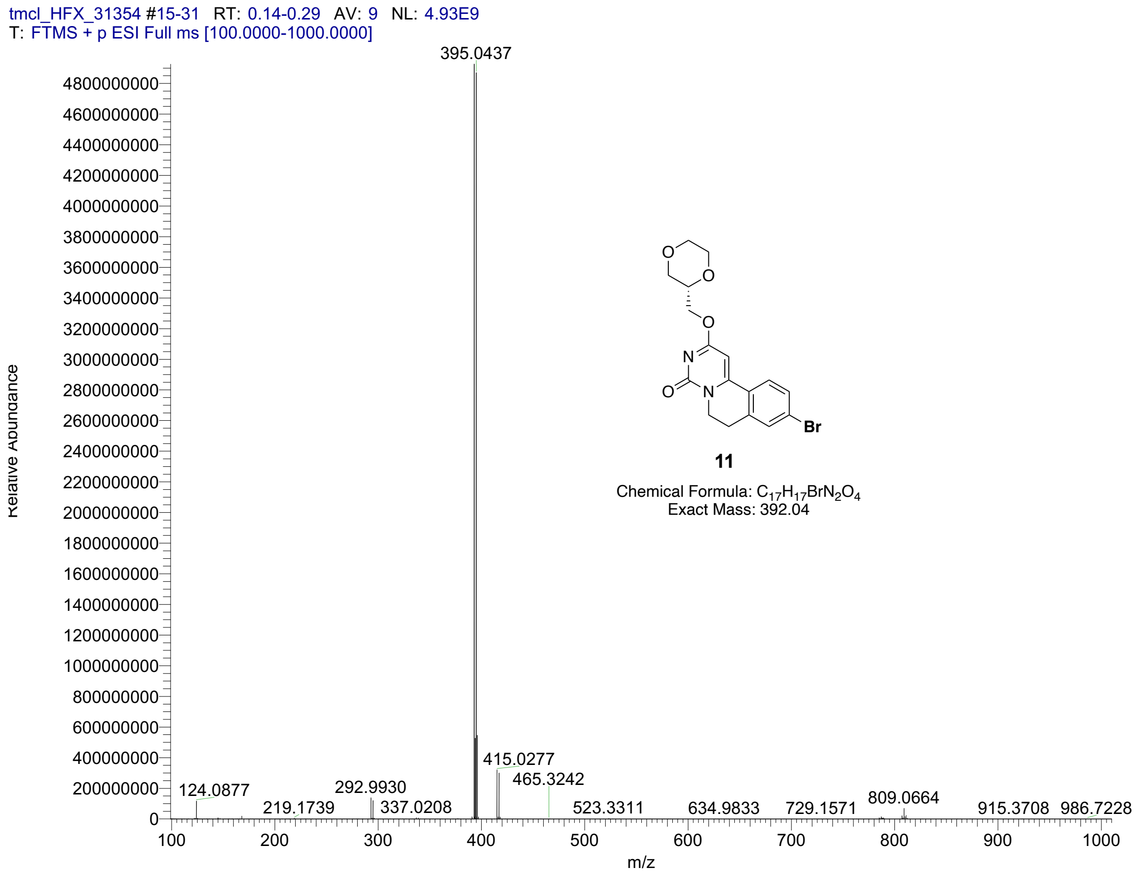

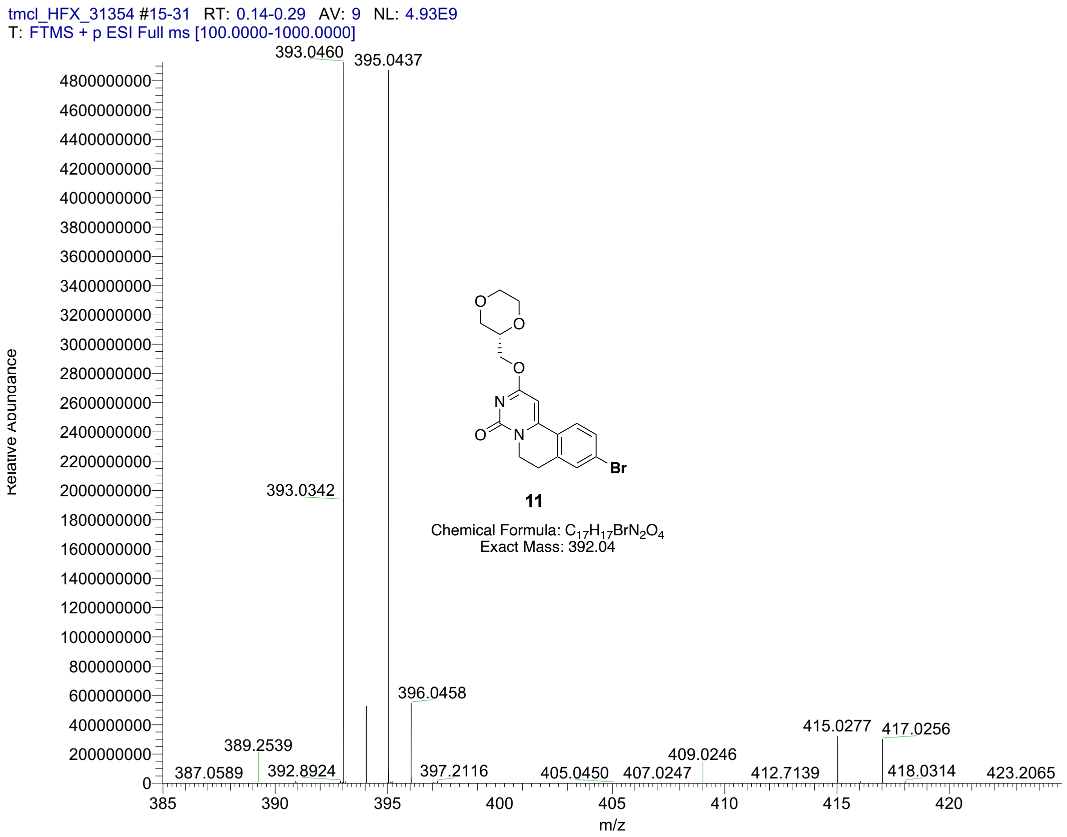

9-bromo-2-[[(2S)-1,4-dioxan-2-yl]methoxy]-6,7-dihydropyrimido[6,1-*a*]isoquinolin-4-one (MGX-110S)

^{1}H NMR (400 MHz, Chloroform-*d*):

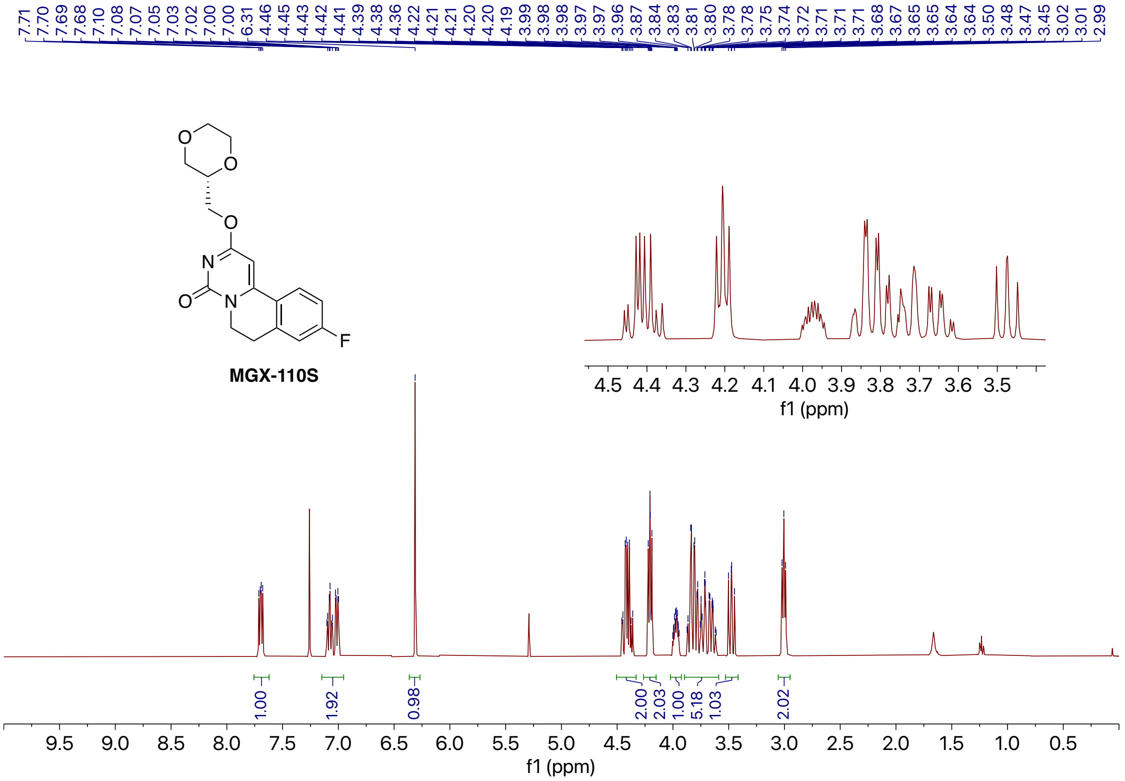

^{13}C NMR (400 MHz, Chloroform-*d*):

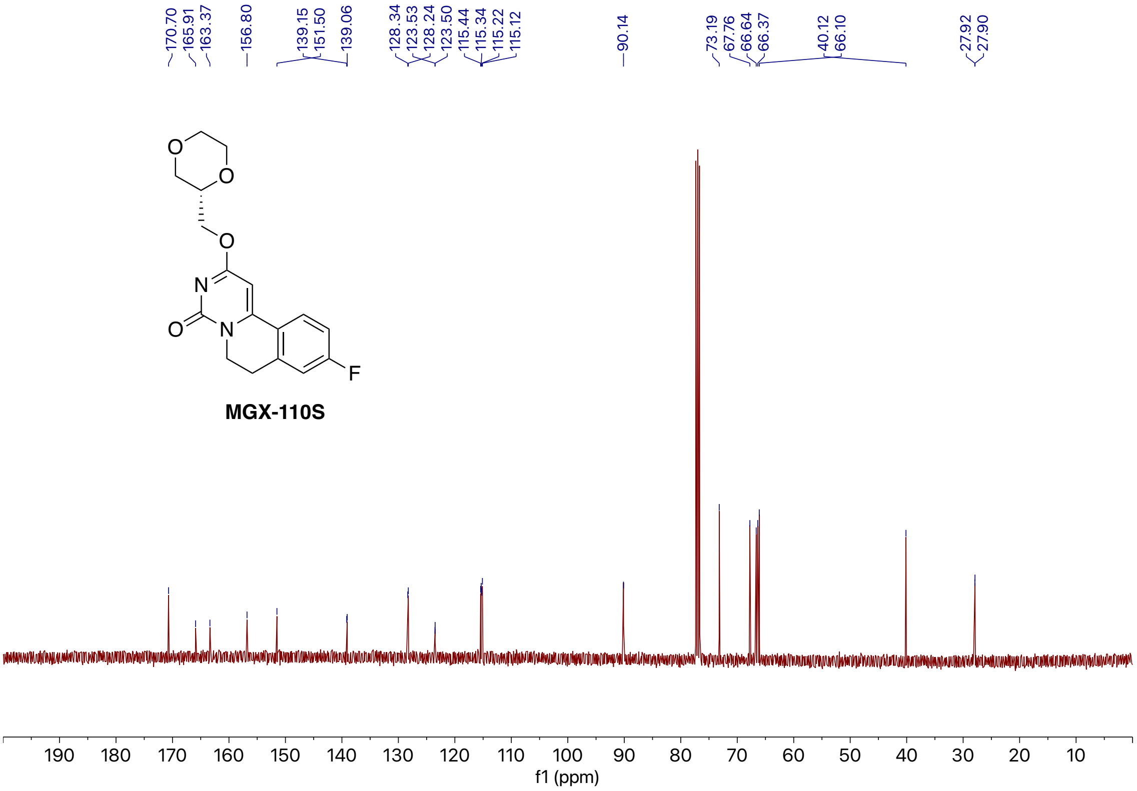

^{19}F NMR (400 MHz, Chloroform-*d*):

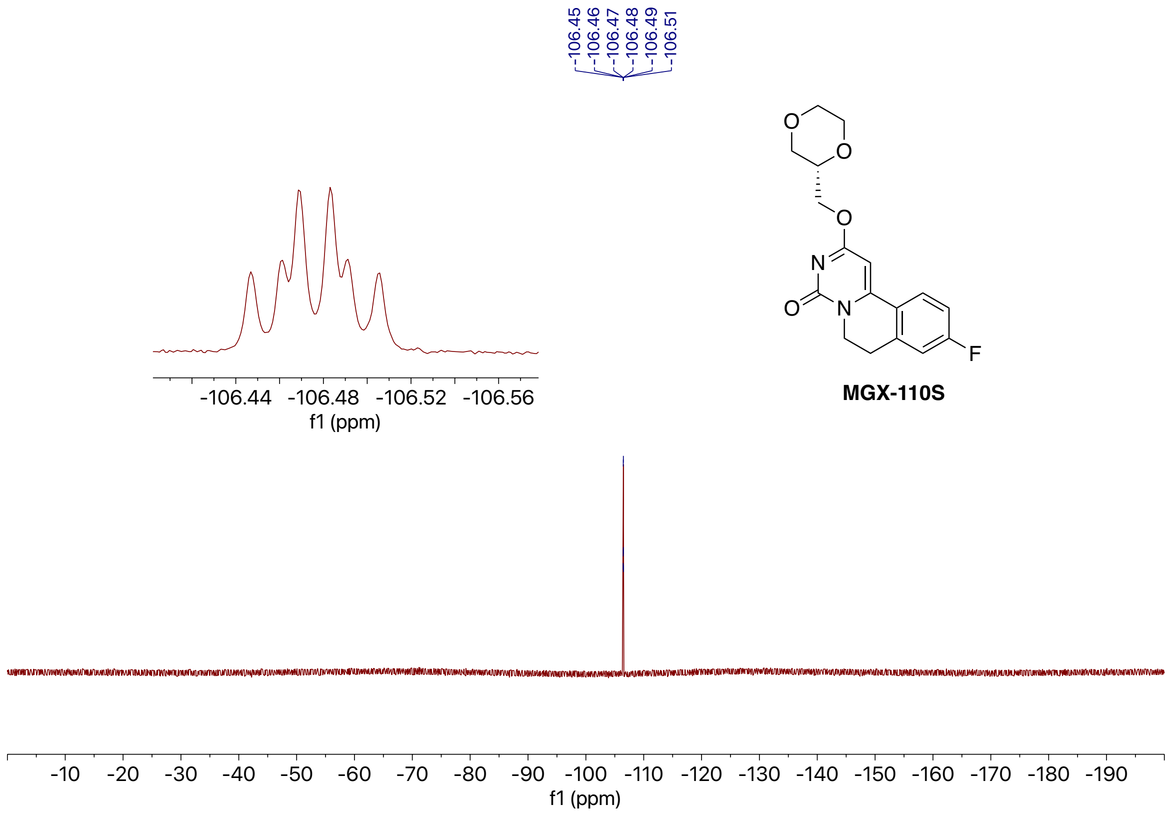

MGX-110S

HRMS (ESI positive mode):

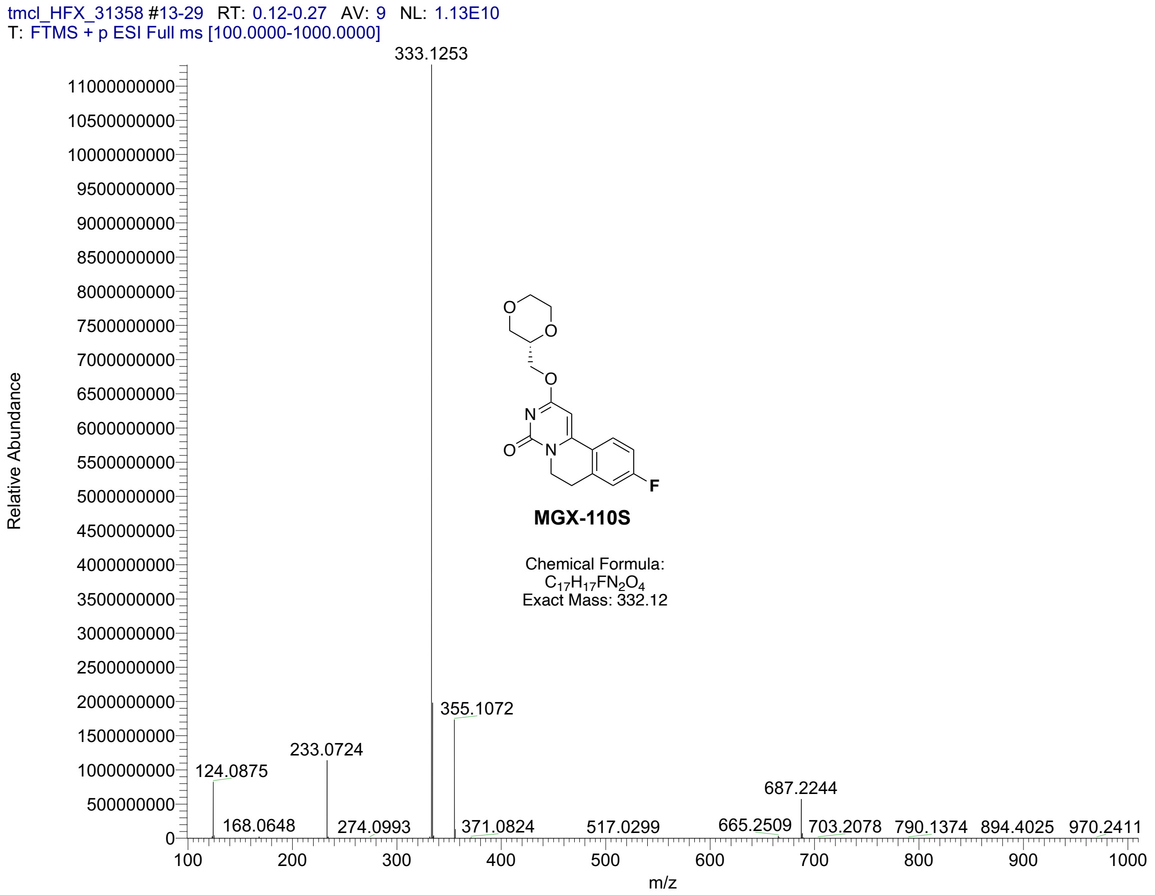

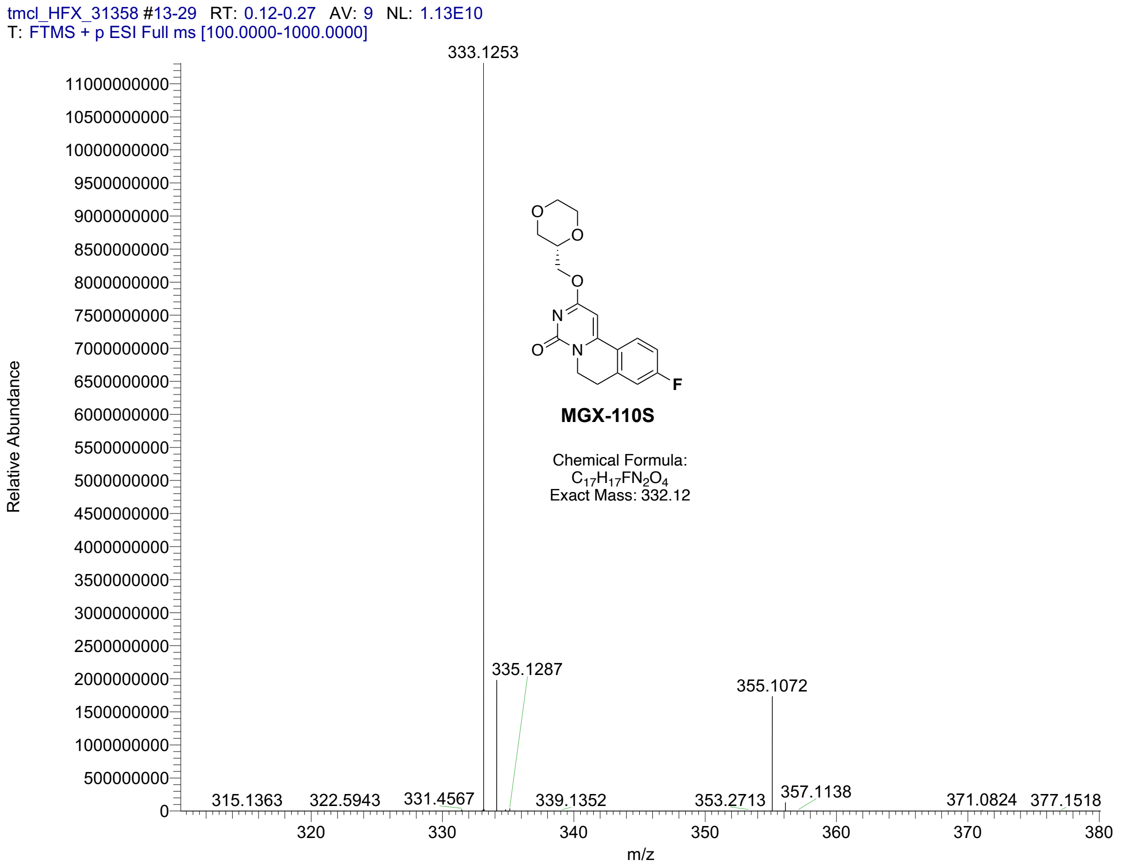

9-bromo-2-[[(2S)-2,3-dihydro-[1,4]dioxino[2,3-*b*]pyridin-2-yl]methoxy]-6,7-dihydropyrimido[6,1-a]isoquinolin-4-one (12)

^{1}H NMR (400 MHz, Chloroform-*d*):

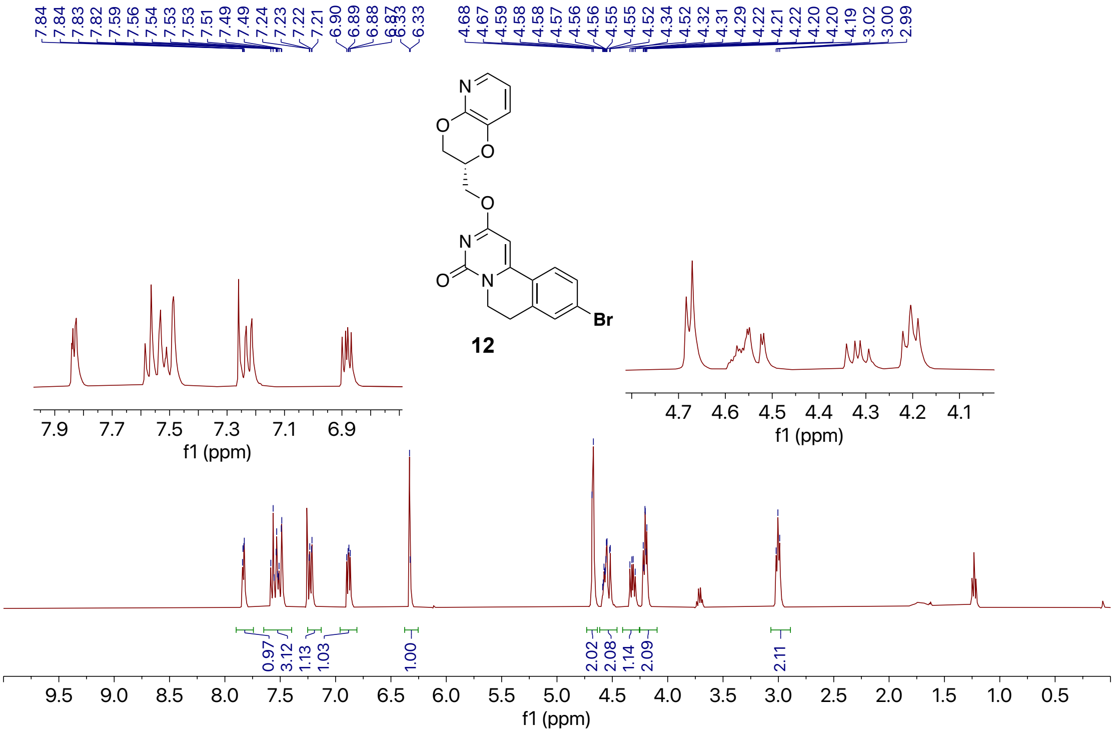

^{13}C NMR (400 MHz, Chloroform-*d*):

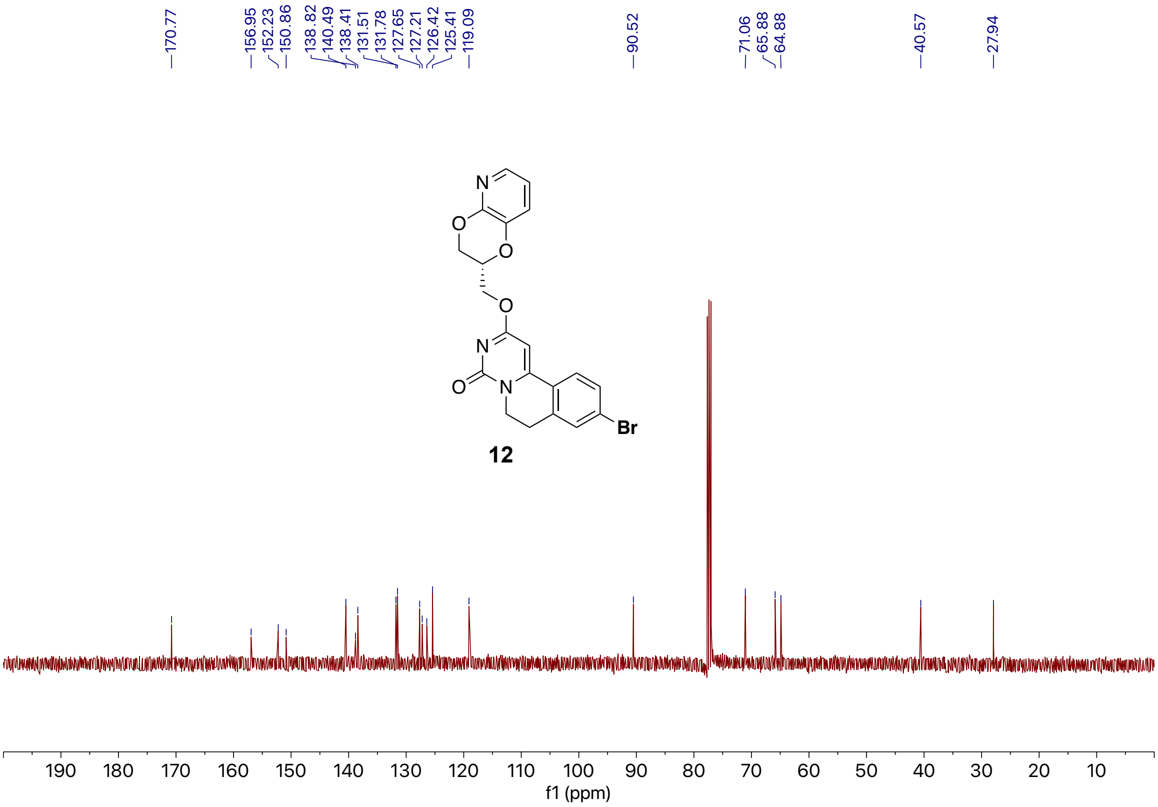

HRMS (ESI positive mode):

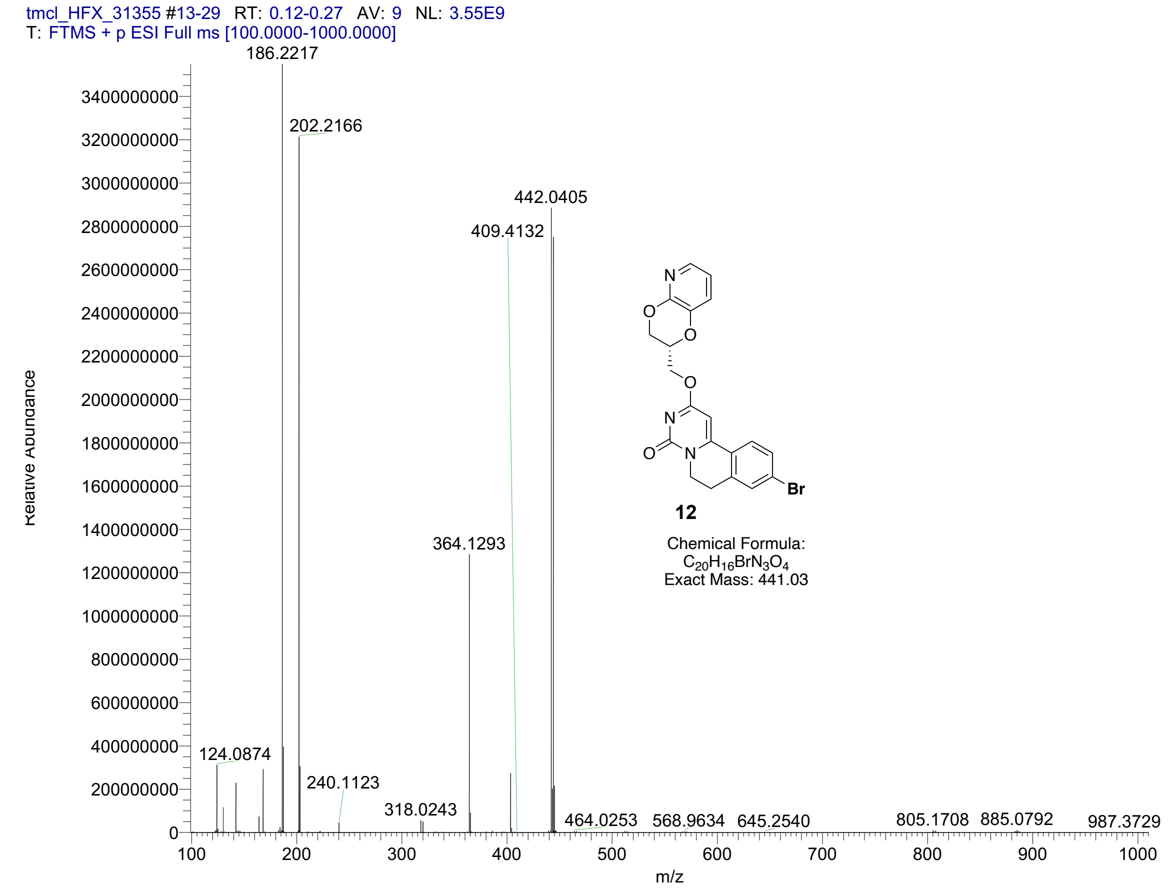

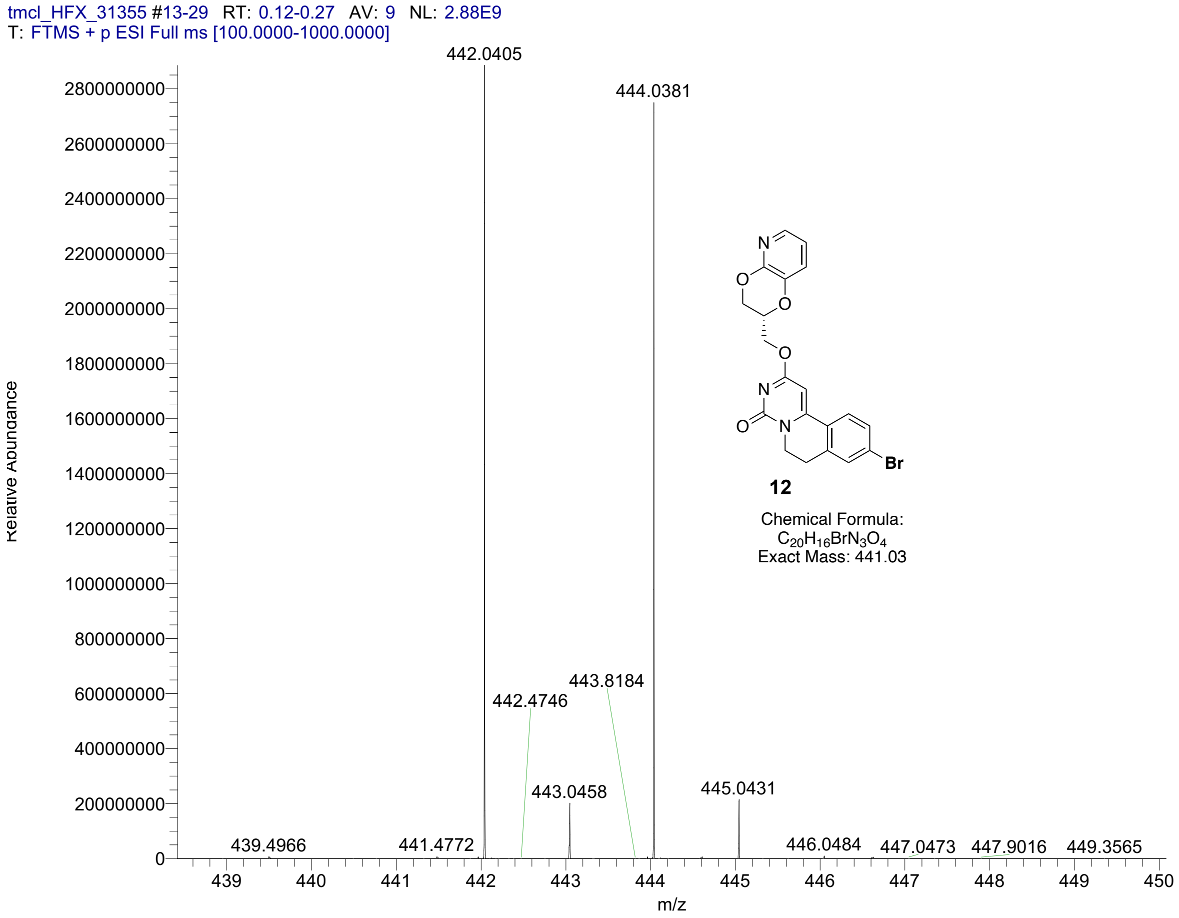

9-halo-2-[[(2S)-2,3-dihydro-[1,4]dioxino[2,3-*b*]pyridin-2-yl]methoxy]-6,7-dihydropyrimido[6,1-a]isoquinolin-4-one (MGX-111S)

^{1}H NMR (400 MHz, Chloroform-*d*):

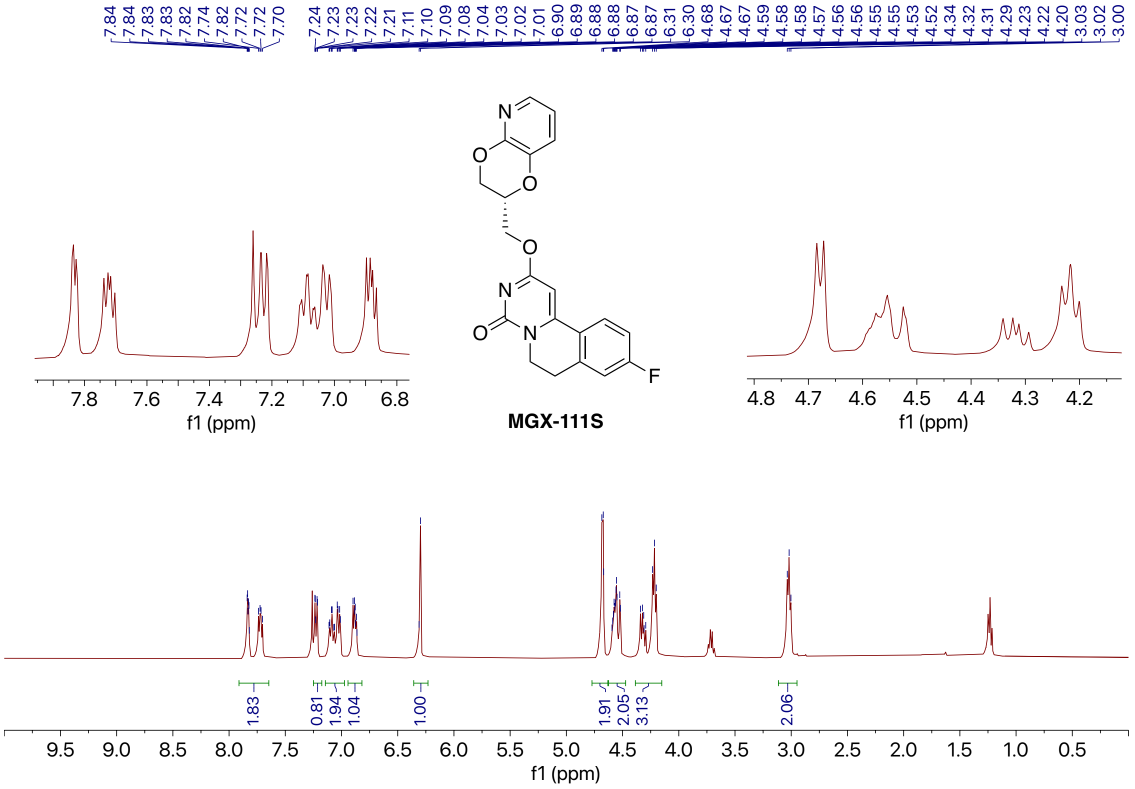

^{13}C NMR (400 MHz, Chloroform-*d*):

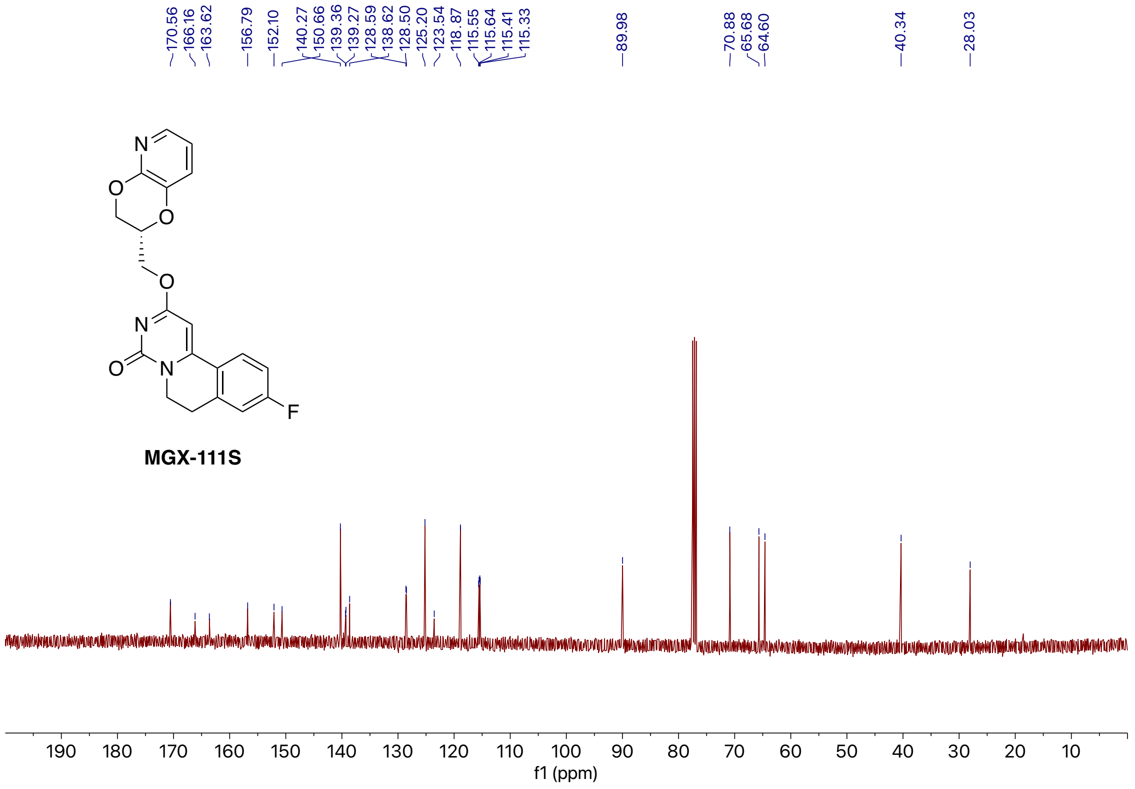

^{19}F NMR (400 MHz, Chloroform-*d*):

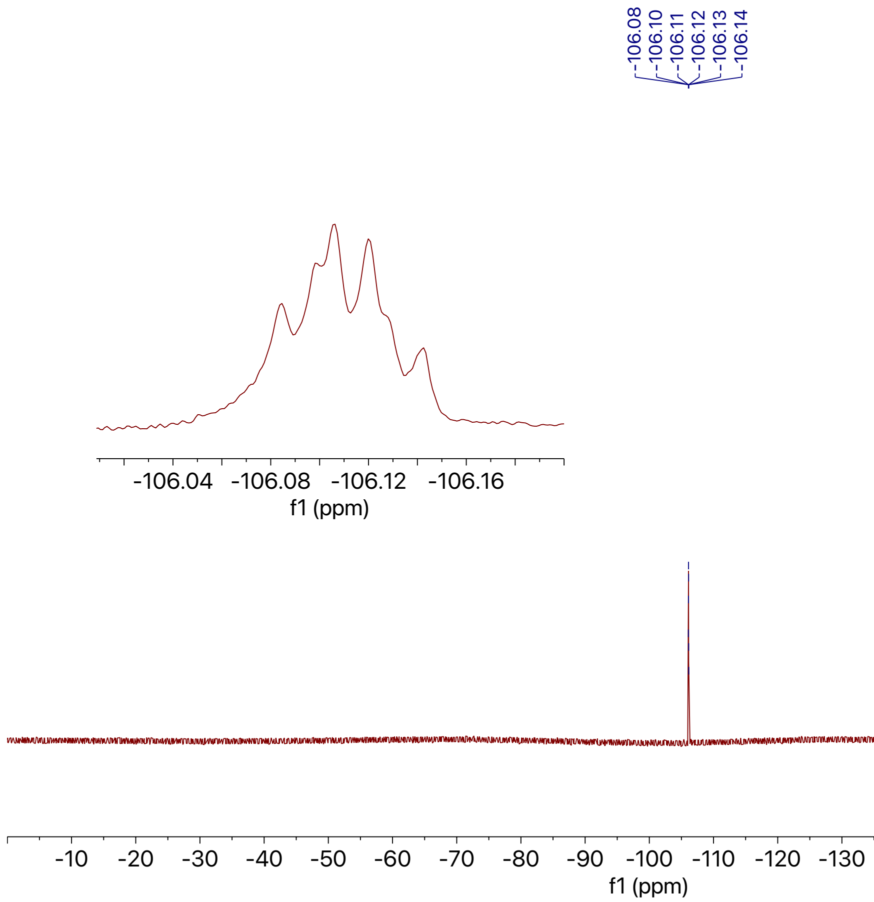

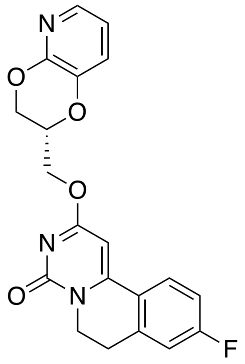

MGX-111S

HRMS (ESI positive mode):

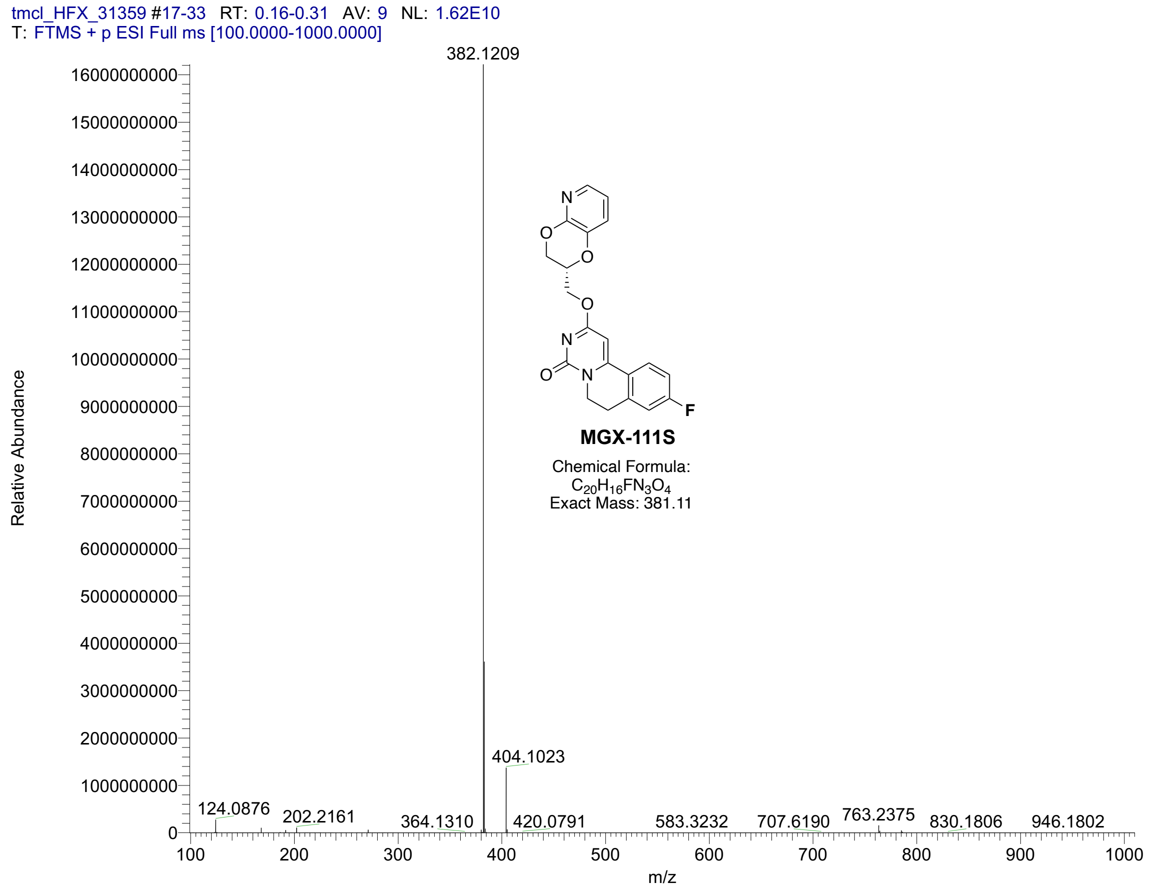

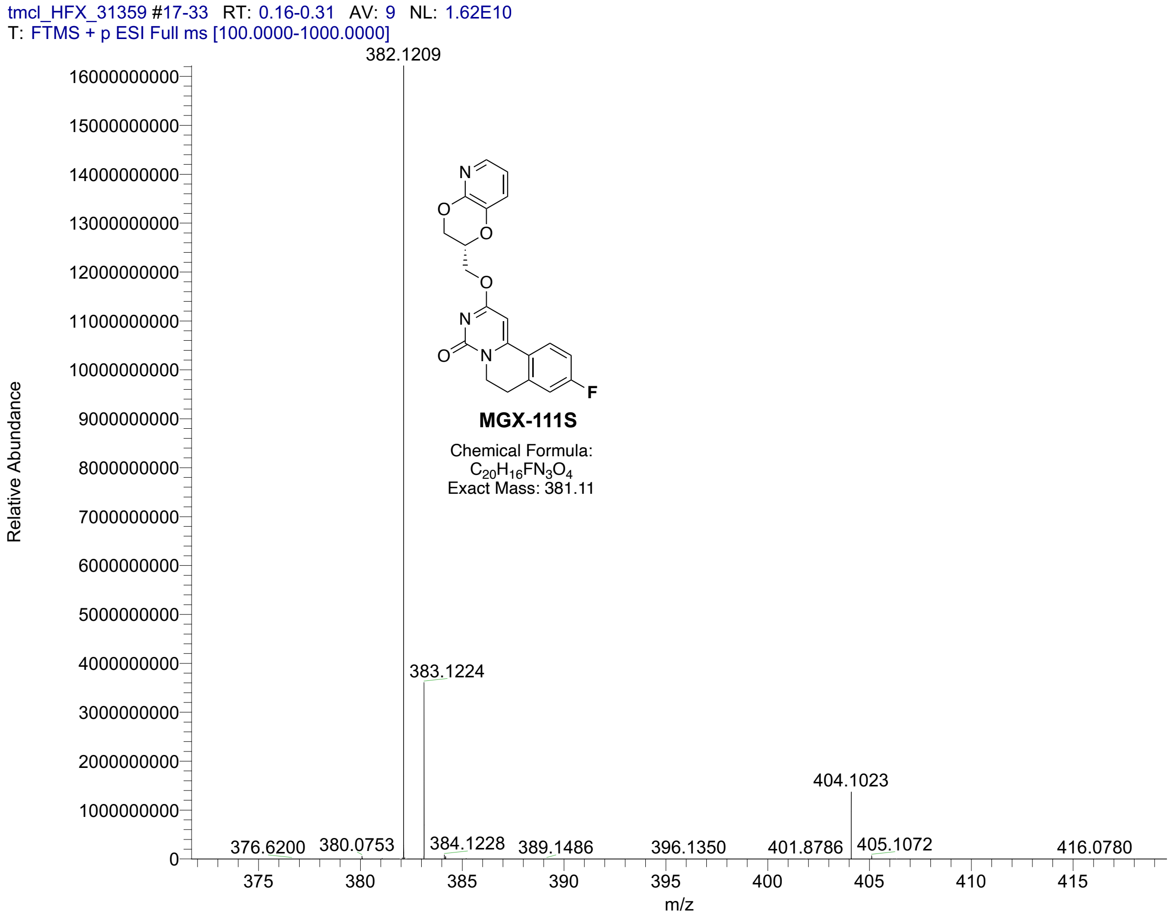

9-*O*-Pinacolatoboro-2-[[(2S)-1,4-dioxan-2-yl]methoxy]-6,7-dihydropyrimido[6,1-a]isoquinolin-4-one (MGX-90S)

^{1}H NMR (400 MHz, Chloroform-*d*):

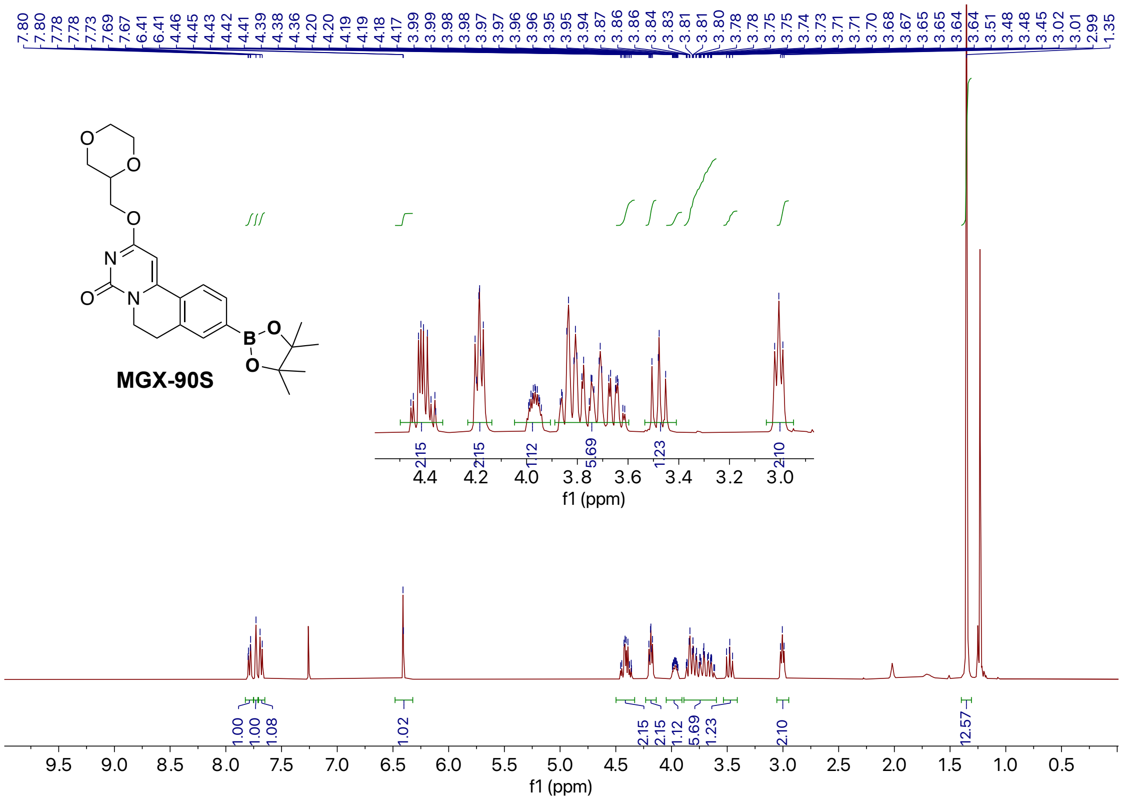

^{13}C NMR (400 MHz, Chloroform-*d*):

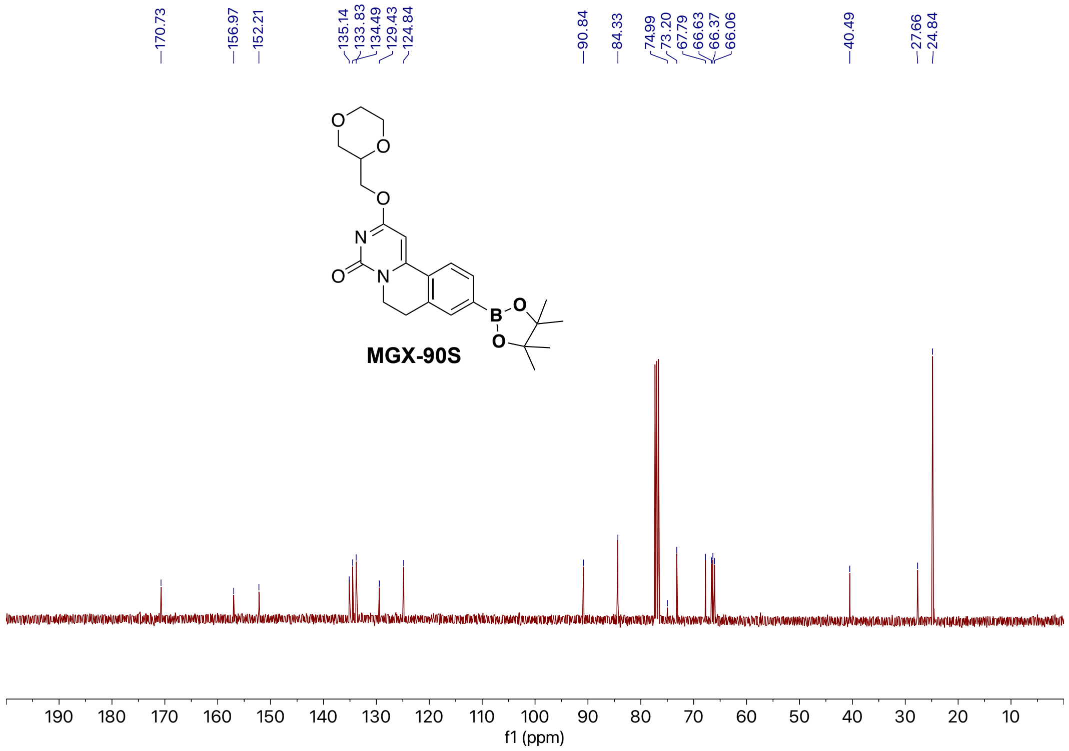

HRMS (ESI positive mode):

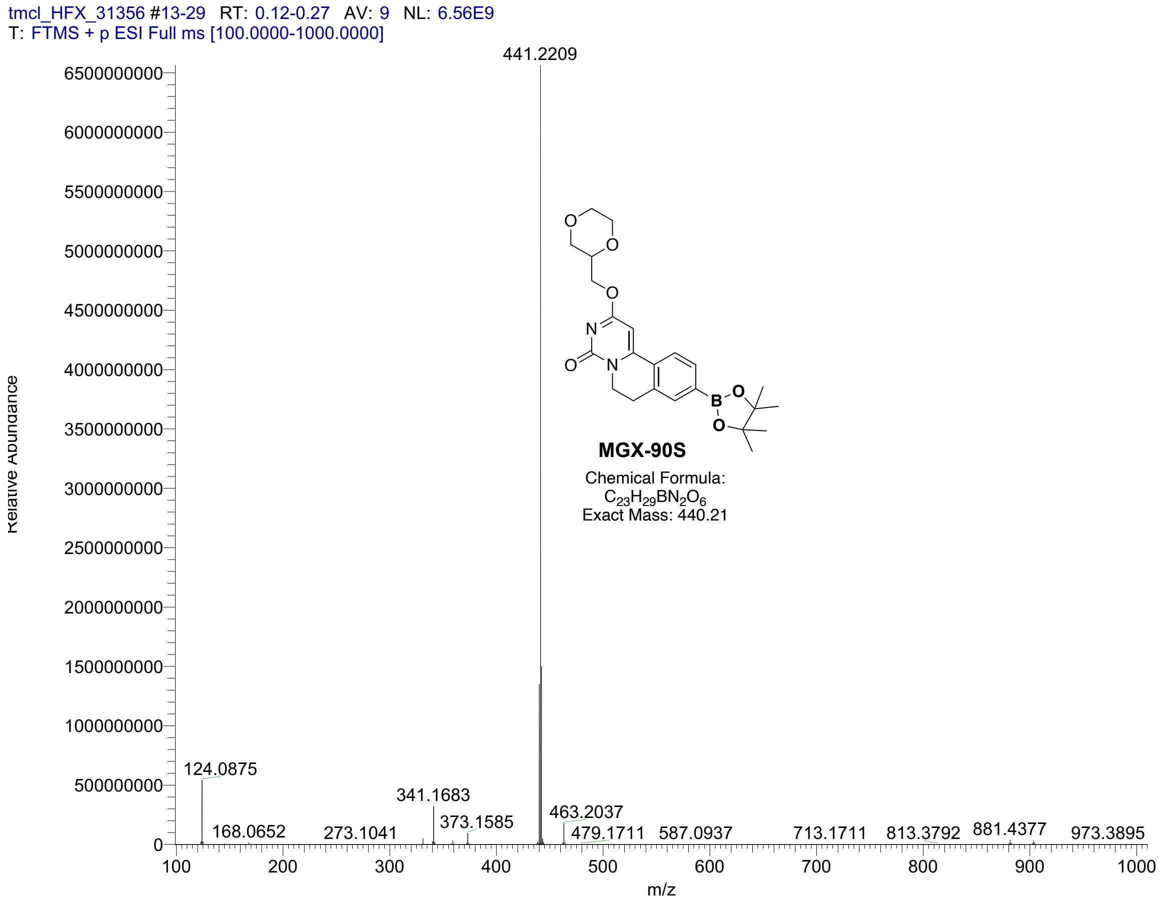

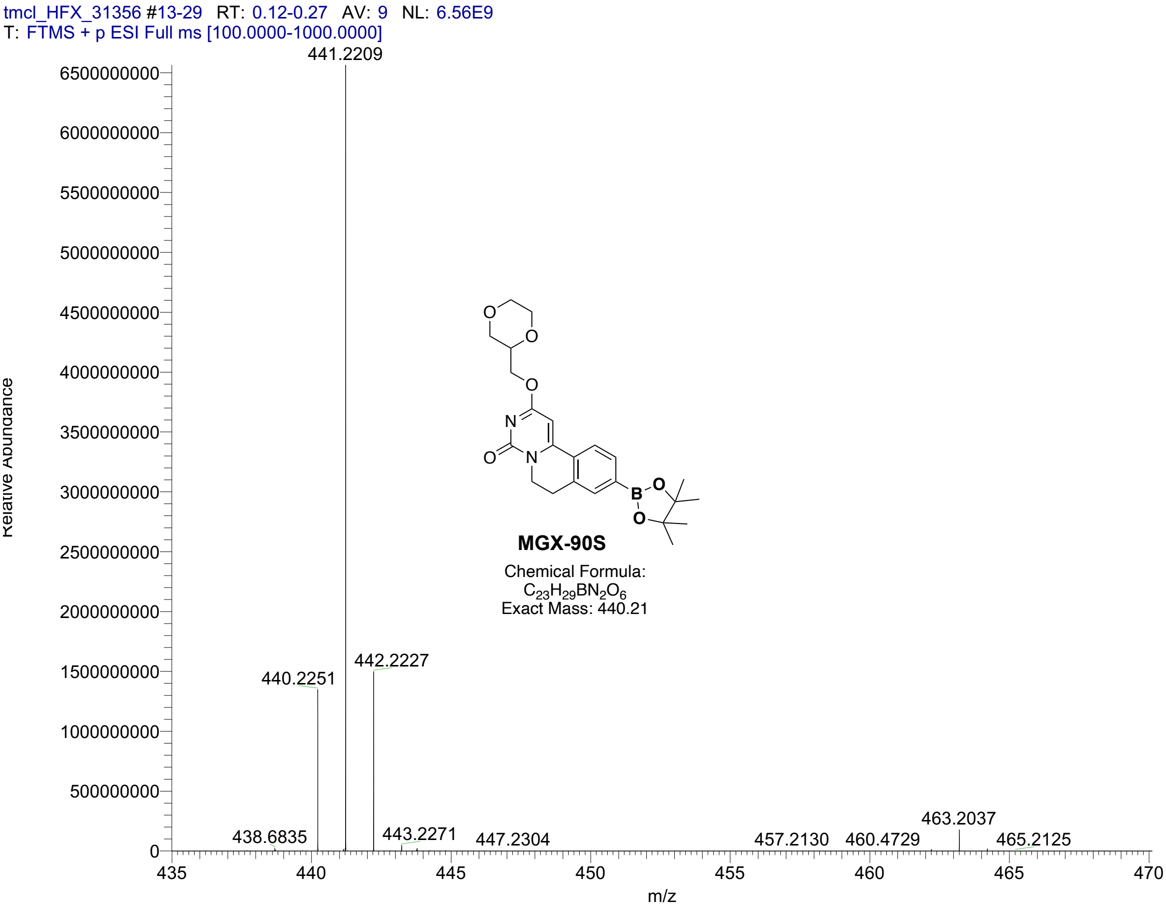

9-*O*-Pinacolatoboro-2-[[(2S)-2,3-dihydro-[1,4]dioxino[2,3-*b*]pyridin-2-yl]methoxy]-6,7-dihydropyrimido[6,1-a]isoquinolin-4-one (MGX-100S)

^{1}H NMR (400 MHz, Chloroform-*d*):

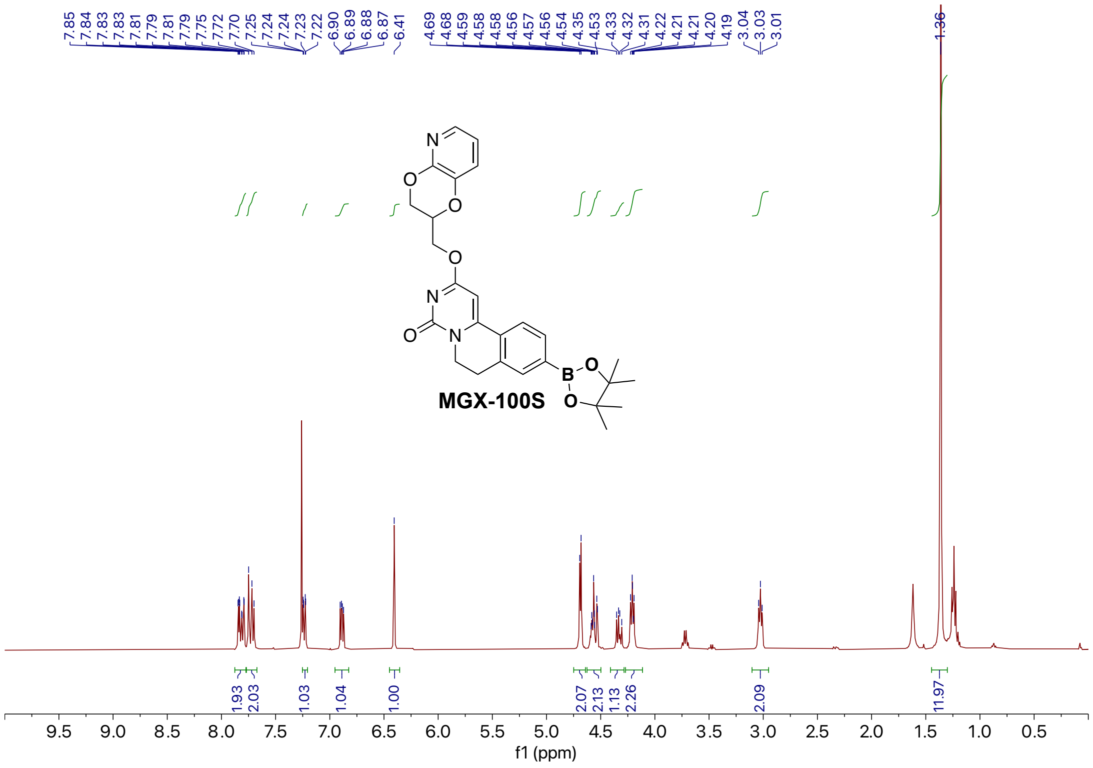

^{13}C NMR (400 MHz, Chloroform-*d*):

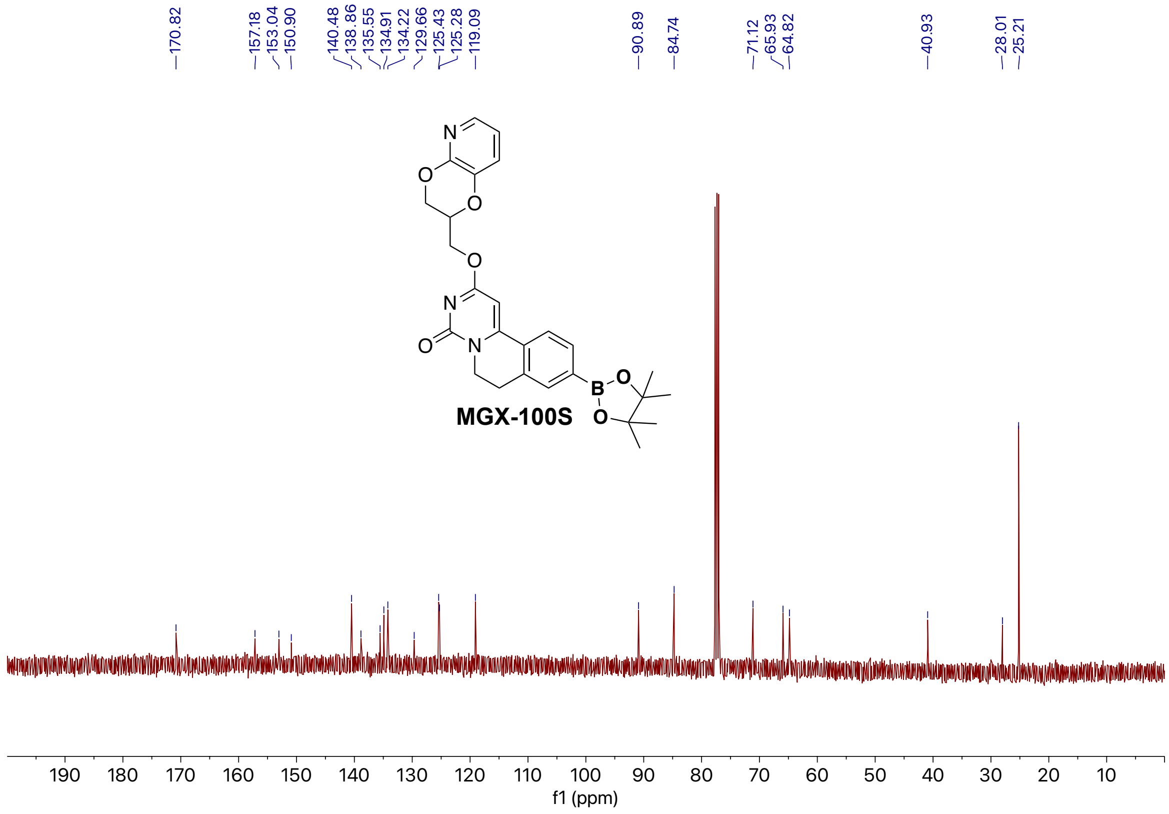

HRMS (ESI positive mode):

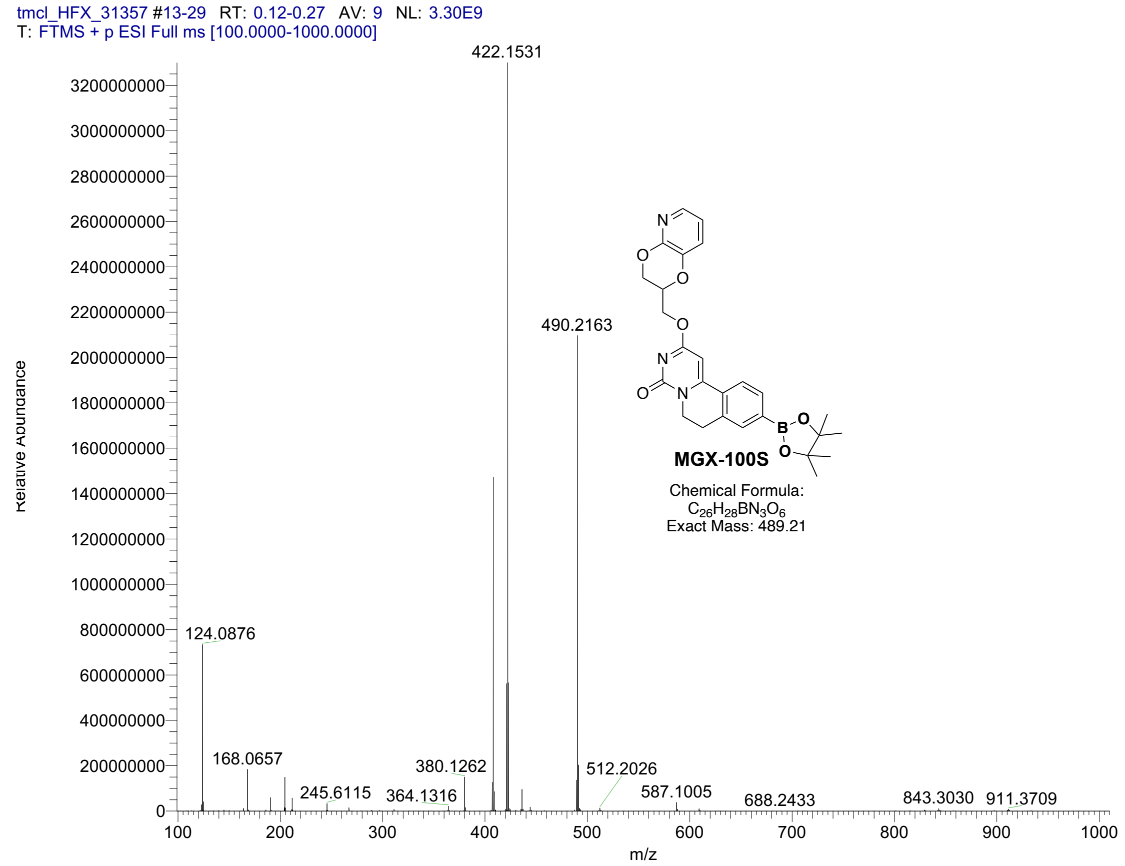

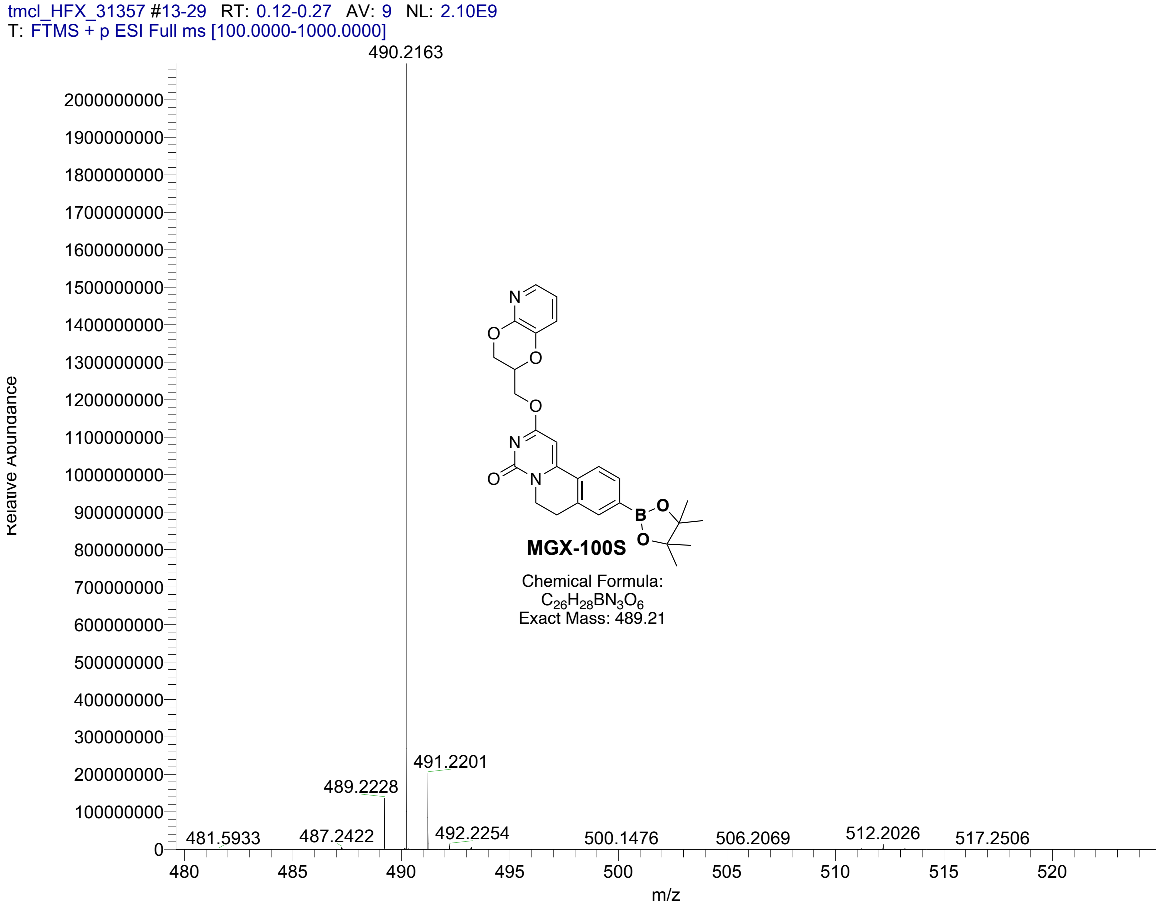

(*S*)-(2-((1,4-dioxan-2-yl)methoxy)-4-oxo-6,7-dihydro-4H-pyrimido[6,1-a]isoquinolin-9-yl)boronic acid (MGX-85S)

^{1}H NMR (400 MHz, DMSO-d_6):

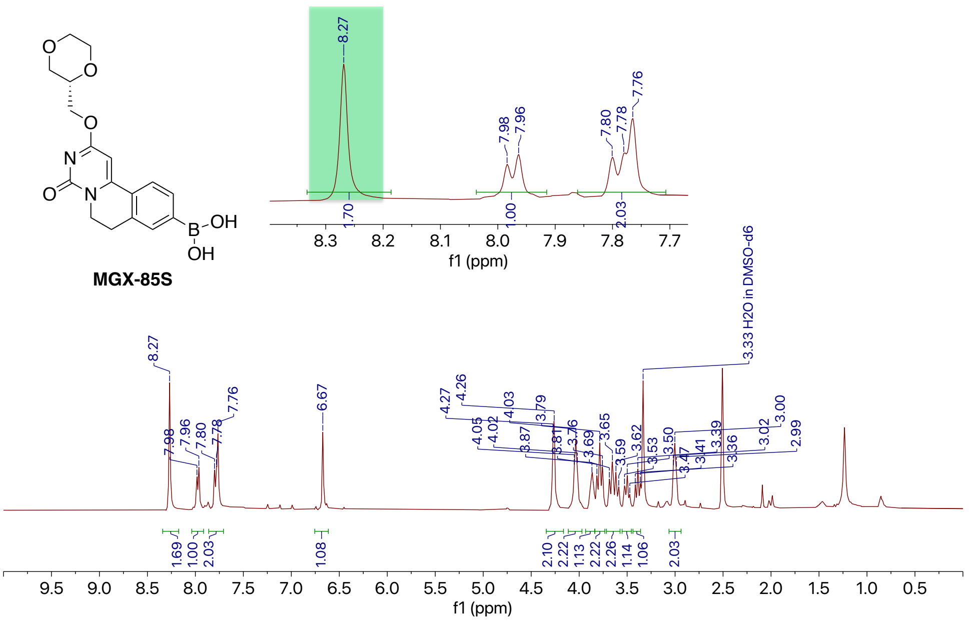

^{1}H NMR (400 MHz, DMSO-d_6 + D_2O)

D_2O/$B(OH)_2$ exchange

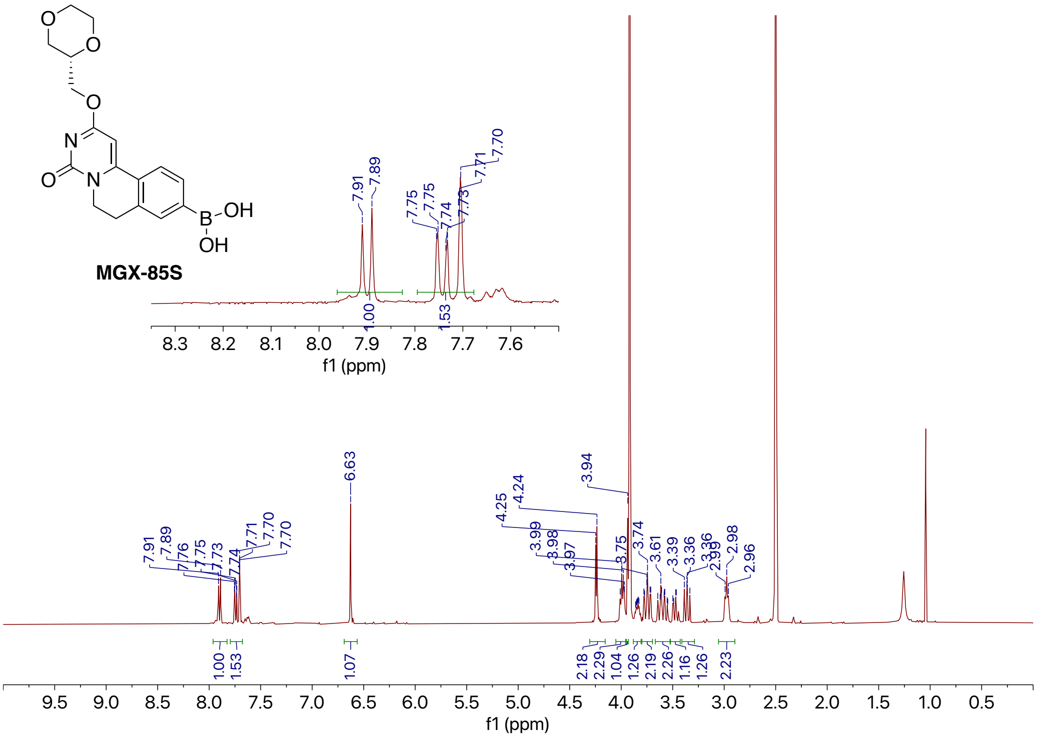

^{13}C NMR (400 MHz, DMSO-d_6):

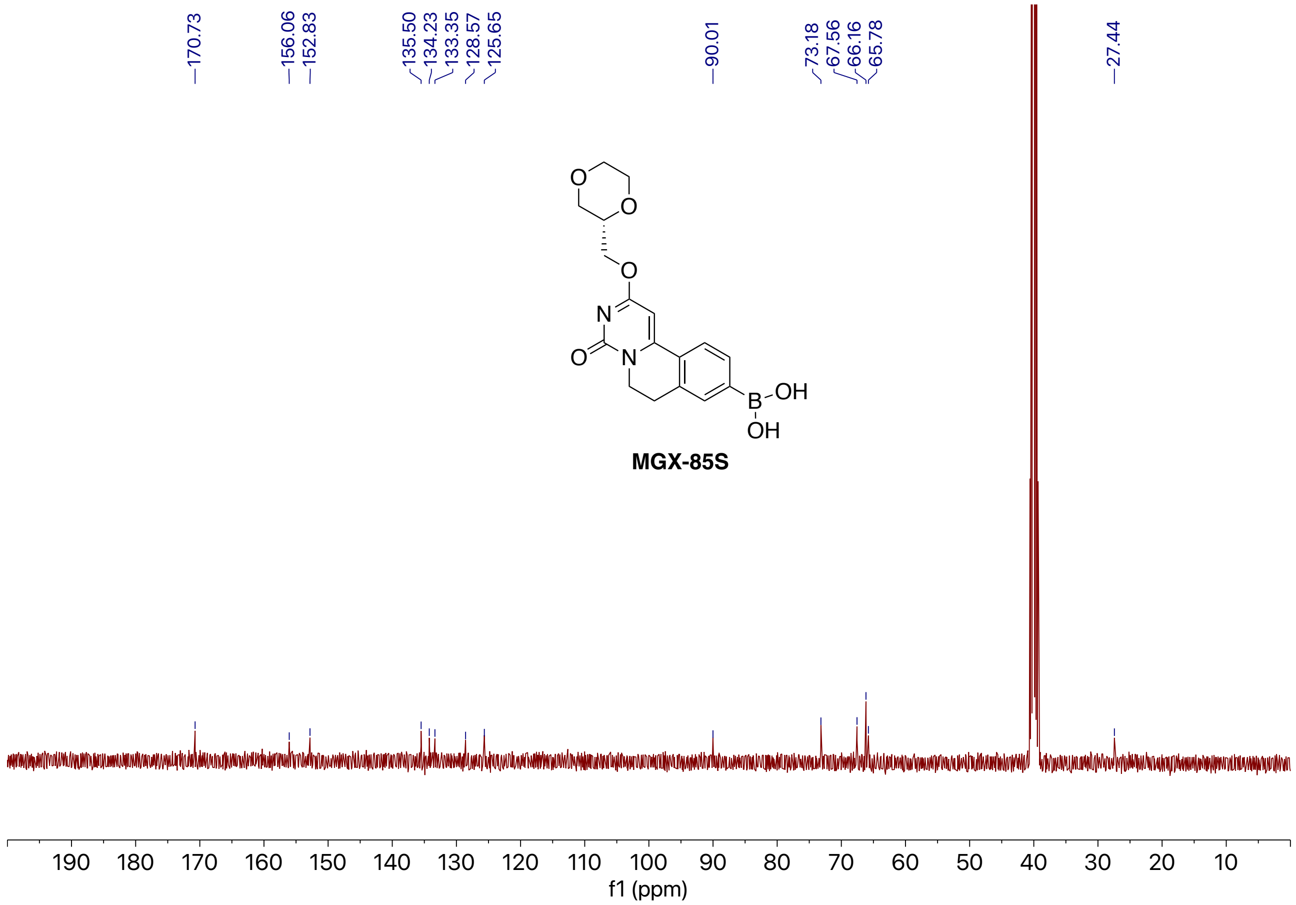

HRMS (ESI positive mode)

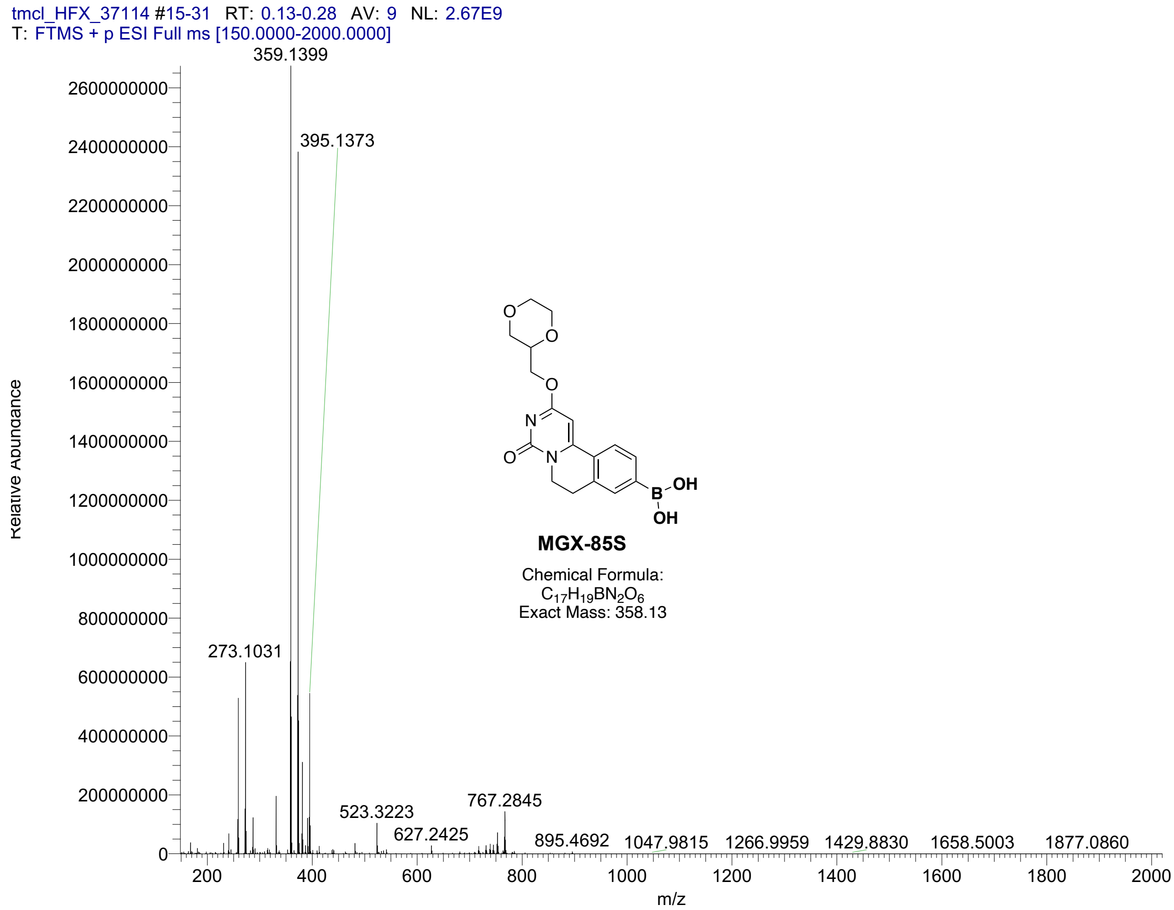

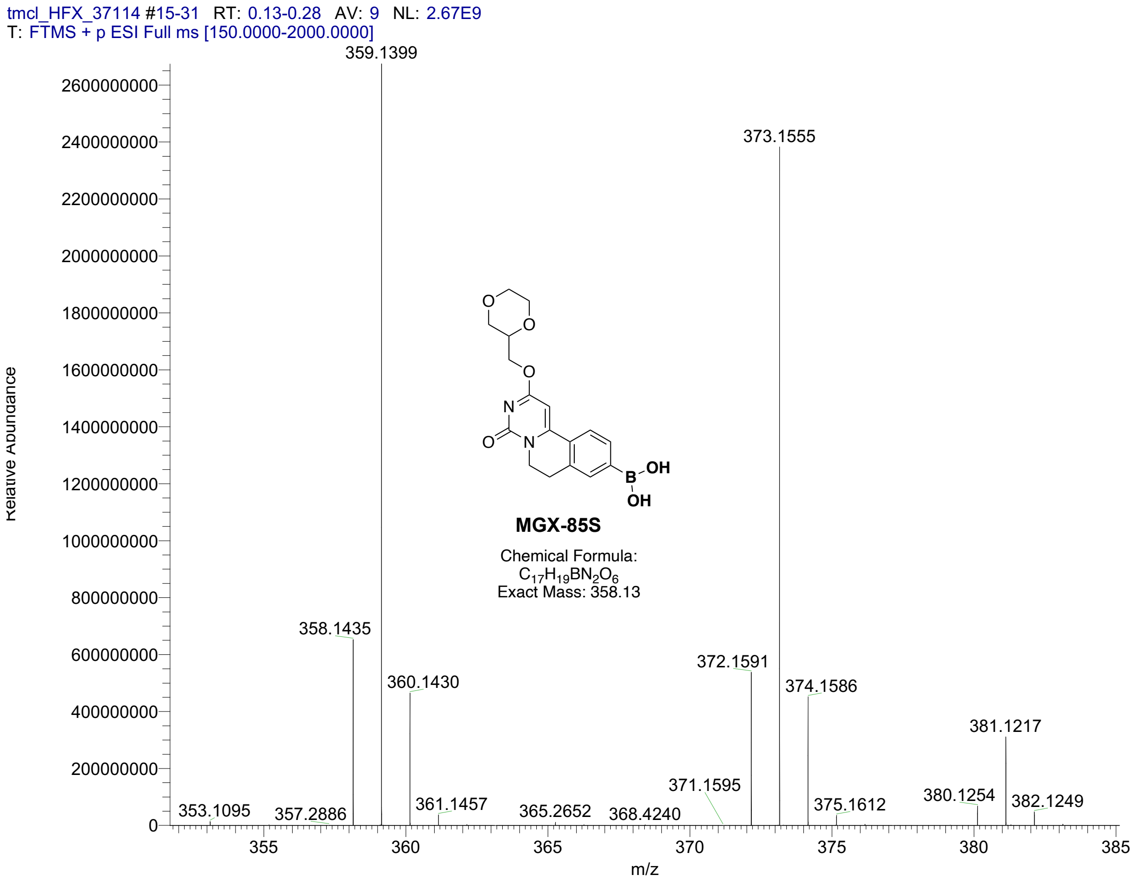

1-(3-bromophenethyl)pyrimidine-2,4,6(1*H*,3*H*,5*H*)-trione (S3)

^{1}H NMR (400 MHz, $CDCl_3$):

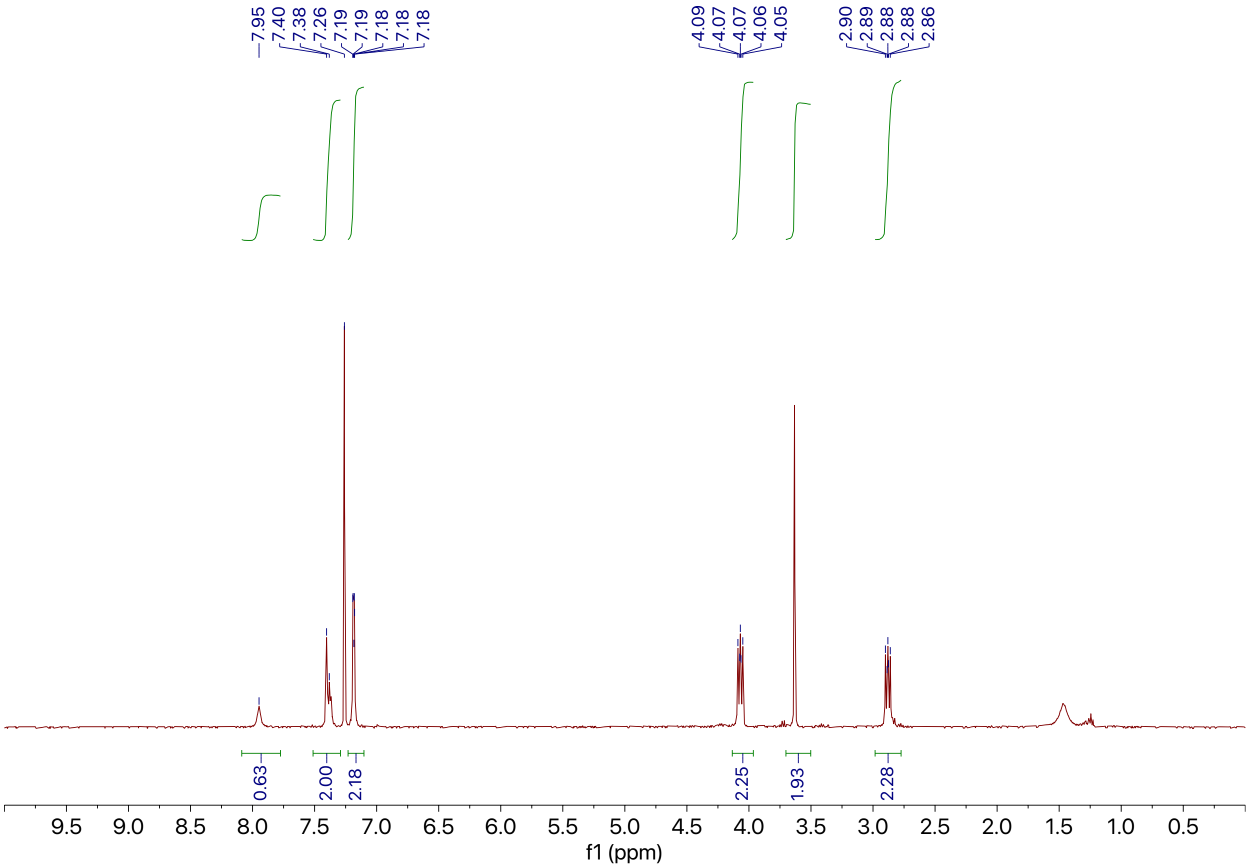

HRMS (ESI positive mode):

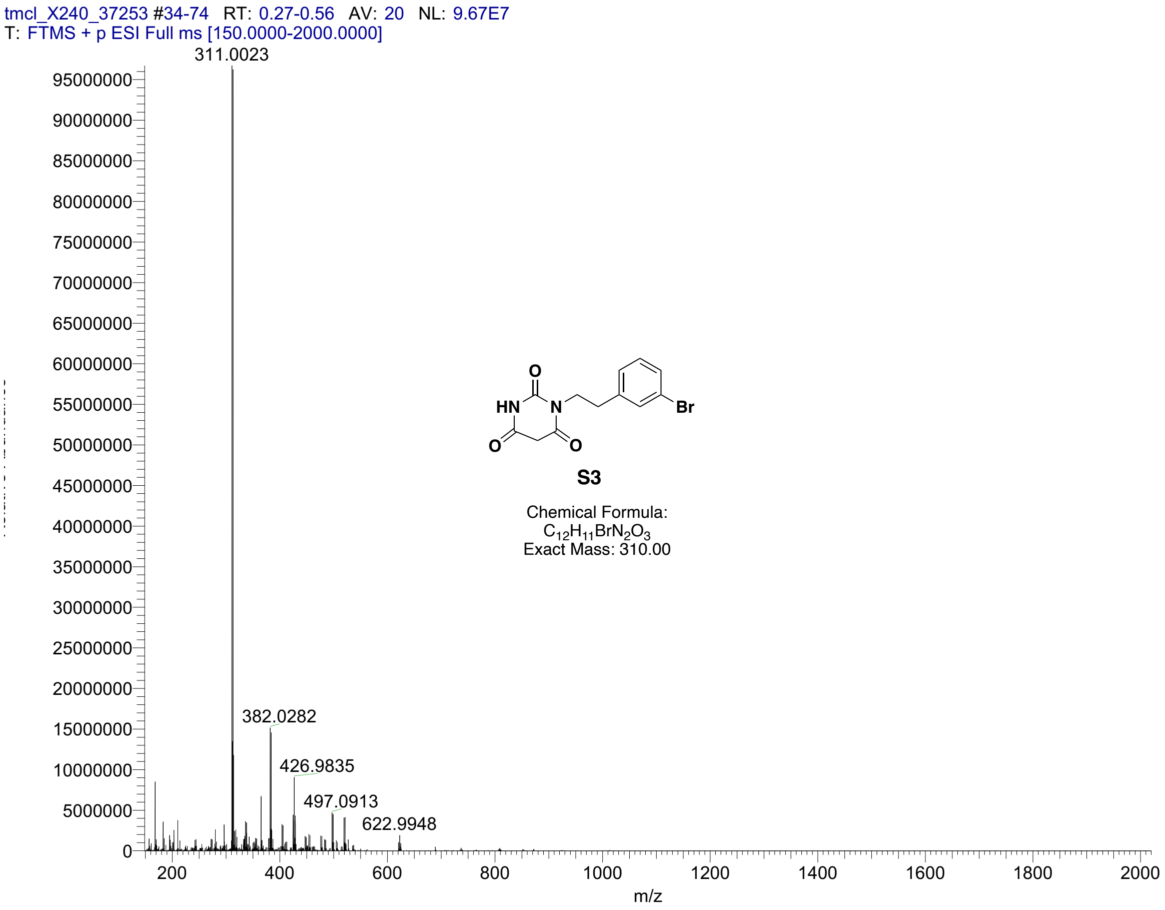

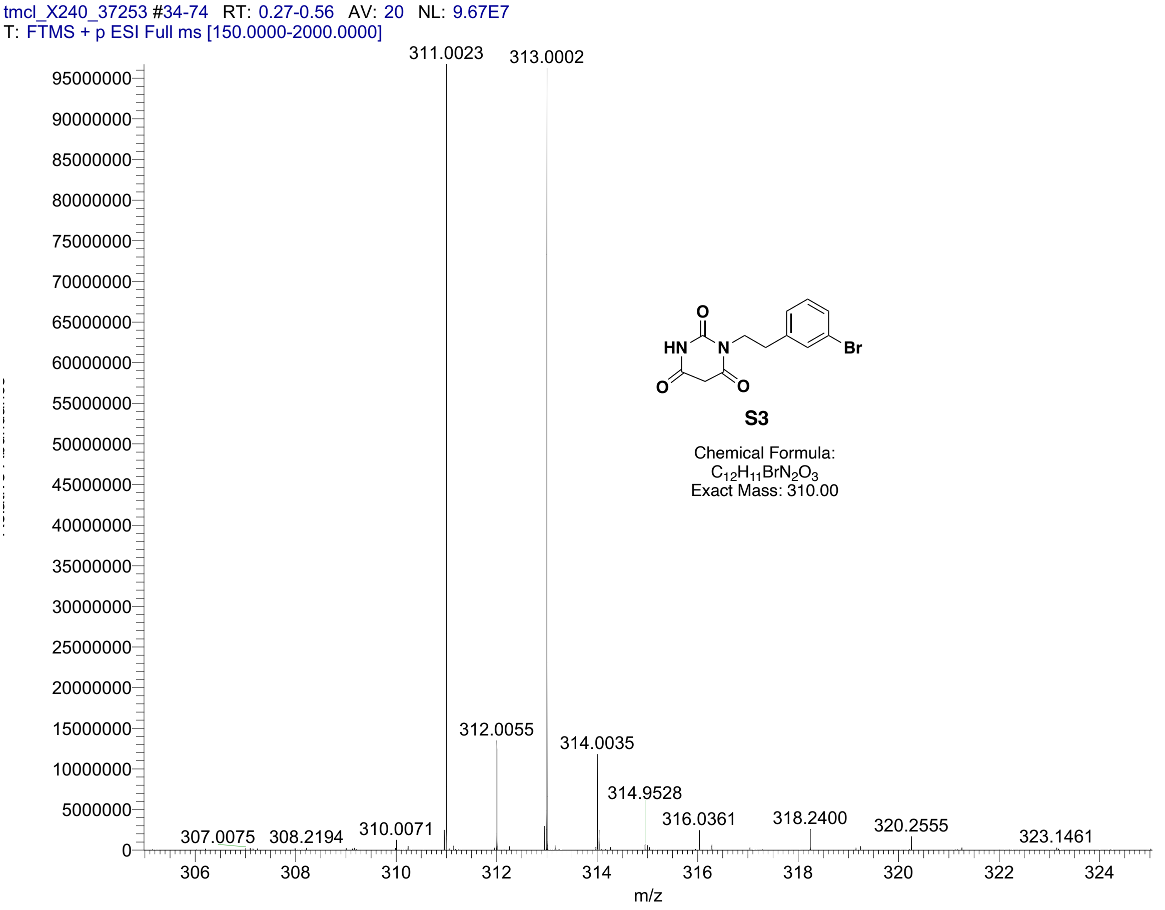

1-(3-bromophenethyl)-6-chloropyrimidine-2,4(1H,3H)-dione (S4)

^{1}H NMR (400 MHz, $CDCl_3$):

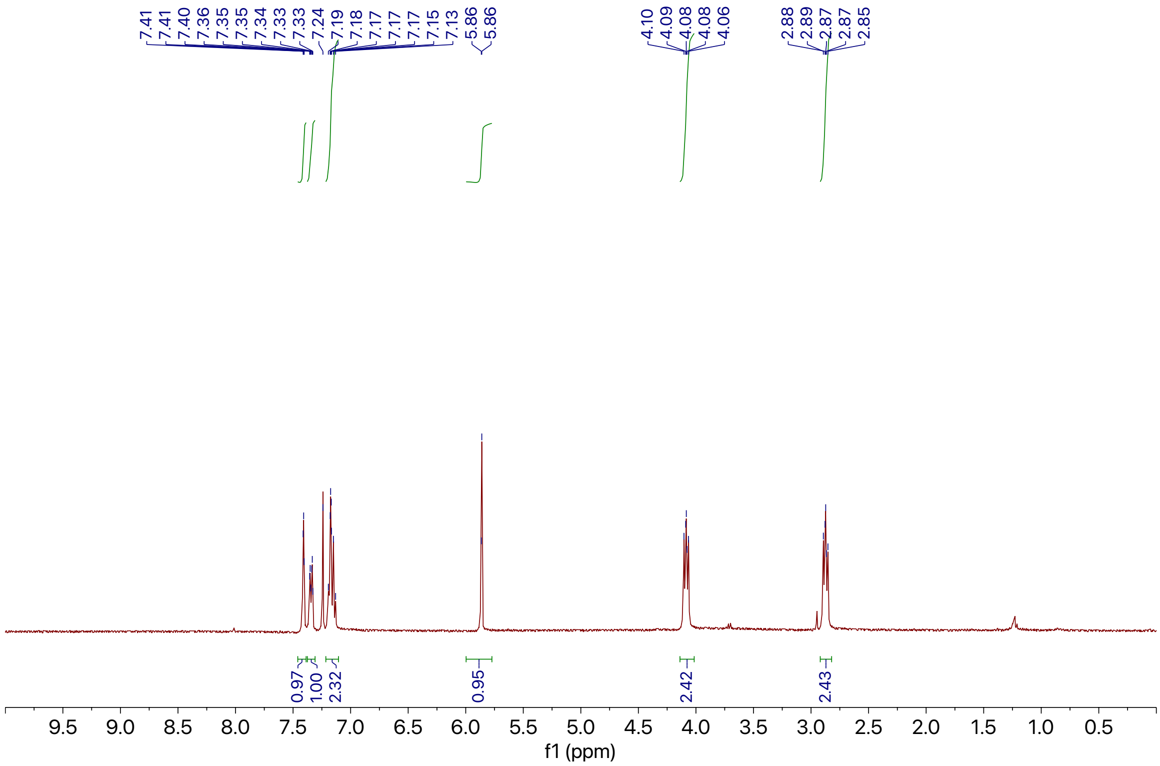

^{13}C NMR (400 MHz, $CDCl_3$):

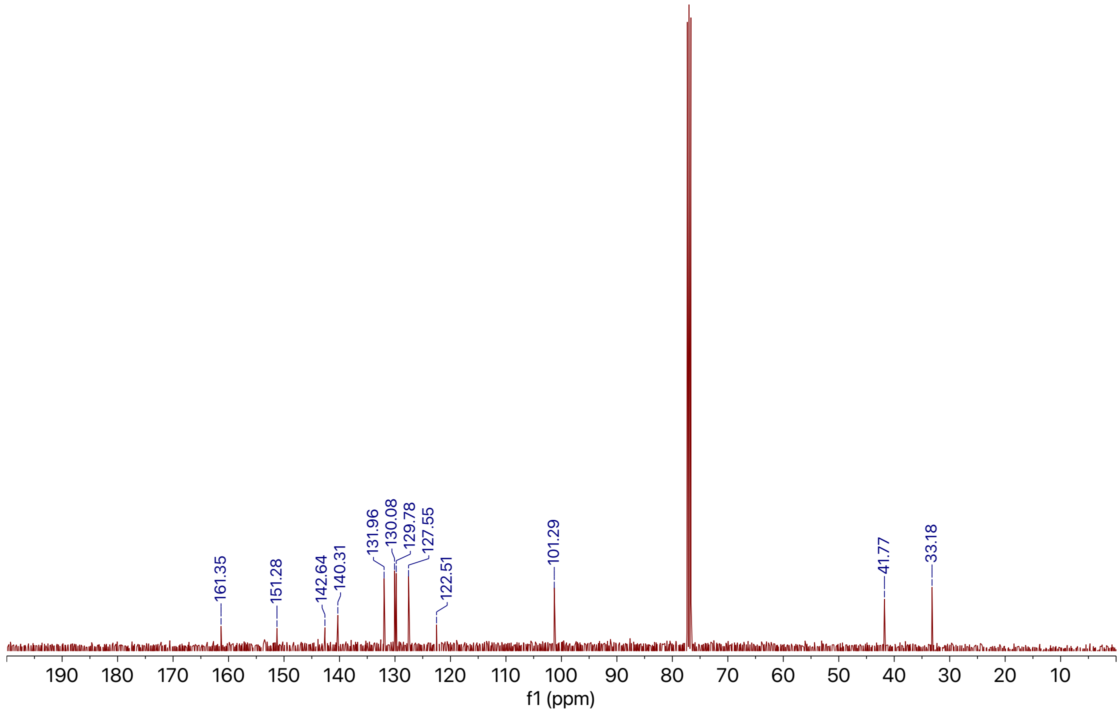

HRMS (ESI negative mode):

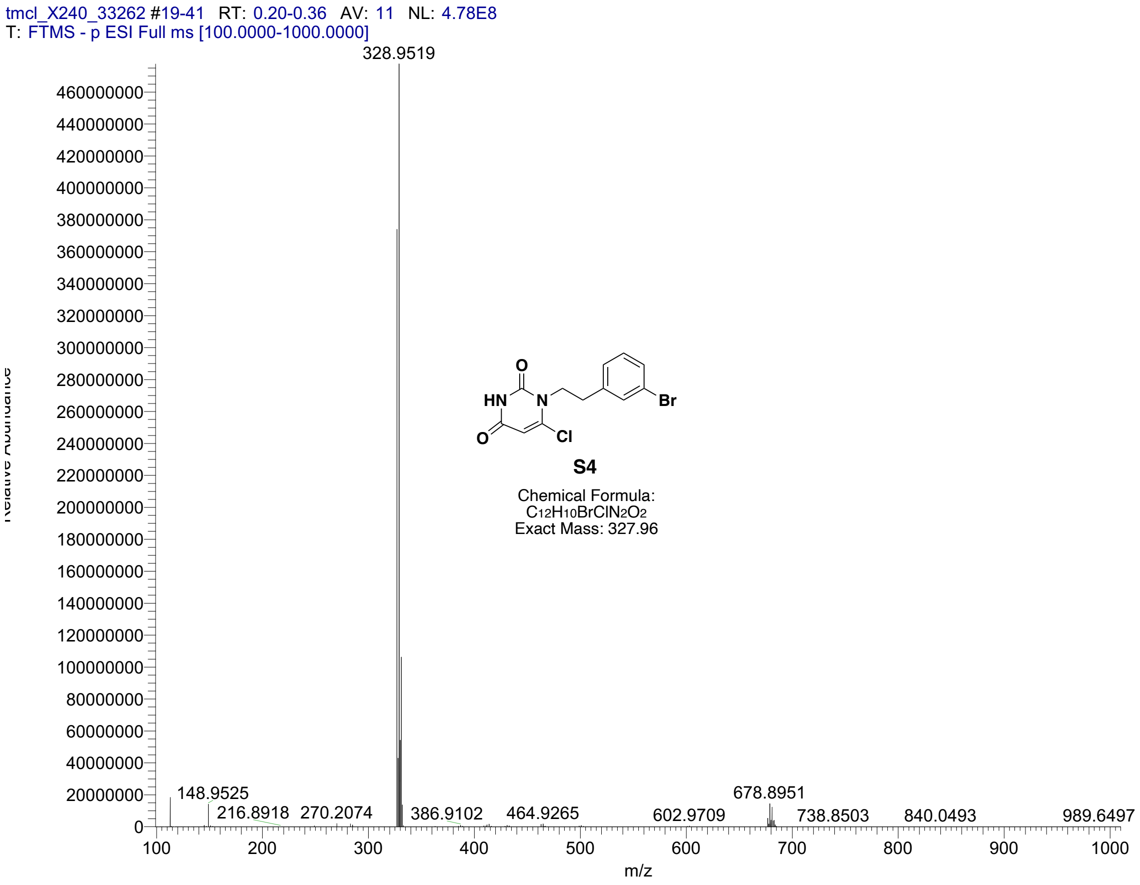

Supplement: Supplementary file 1 — Appendix 01 (PDF) [file pnas.2536372123.sapp.pdf]
